# iAMPCN: a deep-learning approach for identifying antimicrobial peptides and their functional activities

**DOI:** 10.1093/bib/bbad240

**Published:** 2023-06-27

**Authors:** Jing Xu, Fuyi Li, Chen Li, Xudong Guo, Cornelia Landersdorfer, Hsin-Hui Shen, Anton Y Peleg, Jian Li, Seiya Imoto, Jianhua Yao, Tatsuya Akutsu, Jiangning Song

**Affiliations:** Monash Biomedicine Discovery Institute and Department of Biochemistry and Molecular Biology, Monash University, Melbourne, VIC 3800, Australia; Monash Data Futures Institute, Monash University, Melbourne, VIC 3800, Australia; Monash Biomedicine Discovery Institute and Department of Biochemistry and Molecular Biology, Monash University, Melbourne, VIC 3800, Australia; College of Information Engineering, Northwest A&F University, Shaanxi 712100, China; The Peter Doherty Institute for Infection and Immunity, The University of Melbourne, Melbourne, VIC 3800, Australia; Monash Biomedicine Discovery Institute and Department of Biochemistry and Molecular Biology, Monash University, Melbourne, VIC 3800, Australia; Monash Data Futures Institute, Monash University, Melbourne, VIC 3800, Australia; College of Information Engineering, Northwest A&F University, Shaanxi 712100, China; Monash Institute of Pharmaceutical Sciences, Monash University, Melbourne, VIC 3800, Australia; Monash Biomedicine Discovery Institute and Department of Biochemistry and Molecular Biology, Monash University, Melbourne, VIC 3800, Australia; Department of Materials Science and Engineering, Faculty of Engineering, Monash University, Clayton, VIC, 3800, Australia; Monash Biomedicine Discovery Institute and Department of Biochemistry and Molecular Biology, Monash University, Melbourne, VIC 3800, Australia; Department of Infectious Diseases, Alfred Hospital, Alfred Health, Melbourne, Victoria, Australia; Monash Biomedicine Discovery Institute and Department of Microbiology, Monash University, Melbourne, VIC 3800, Australia; Division of Health Medical Intelligence, Human Genome Center, Institute of Medical Science, The University of Tokyo, Minato-ku, Tokyo, Japan; Collaborative Research Institute for Innovative Microbiology, The University of Tokyo, Bunkyo-ku, Tokyo, Japan; Tencent AI Lab, Tencent, Shenzhen, China; Bioinformatics Center, Institute for Chemical Research, Kyoto University, Uji 611-0011, Japan; Monash Biomedicine Discovery Institute and Department of Biochemistry and Molecular Biology, Monash University, Melbourne, VIC 3800, Australia; Monash Data Futures Institute, Monash University, Melbourne, VIC 3800, Australia; Bioinformatics Center, Institute for Chemical Research, Kyoto University, Uji 611-0011, Japan

**Keywords:** antimicrobial peptides, bioinformatics, sequence analysis, machine learning, deep learning, functional activities

## Abstract

Antimicrobial peptides (AMPs) are short peptides that play crucial roles in diverse biological processes and have various functional activities against target organisms. Due to the abuse of chemical antibiotics and microbial pathogens’ increasing resistance to antibiotics, AMPs have the potential to be alternatives to antibiotics. As such, the identification of AMPs has become a widely discussed topic. A variety of computational approaches have been developed to identify AMPs based on machine learning algorithms. However, most of them are not capable of predicting the functional activities of AMPs, and those predictors that can specify activities only focus on a few of them. In this study, we first surveyed 10 predictors that can identify AMPs and their functional activities in terms of the features they employed and the algorithms they utilized. Then, we constructed comprehensive AMP datasets and proposed a new deep learning-based framework, iAMPCN (identification of AMPs based on CNNs), to identify AMPs and their related 22 functional activities. Our experiments demonstrate that iAMPCN significantly improved the prediction performance of AMPs and their corresponding functional activities based on four types of sequence features. Benchmarking experiments on the independent test datasets showed that iAMPCN outperformed a number of state-of-the-art approaches for predicting AMPs and their functional activities. Furthermore, we analyzed the amino acid preferences of different AMP activities and evaluated the model on datasets of varying sequence redundancy thresholds. To facilitate the community-wide identification of AMPs and their corresponding functional types, we have made the source codes of iAMPCN publicly available at https://github.com/joy50706/iAMPCN/tree/master. We anticipate that iAMPCN can be explored as a valuable tool for identifying potential AMPs with specific functional activities for further experimental validation.

## INTRODUCTION

Due to the microbial pathogens’ increasing resistance to chemical antibiotics, it is urgent to develop novel infectious therapeutics [1, 2]. Over the past decade, there have been several developments in utilizing antimicrobial peptides (AMPs) as potential alternatives to treat infections since most natural AMPs are particular polypeptide substances in living organisms and are critical components of the innate immune system which protects the host against invading pathogens [3]. AMPs are generally small-molecule polypeptides and have diverse functional activities against target organisms such as bacteria, yeasts, fungi, viruses and cancer cells. Compared with traditional chemical antibiotics, AMPs have higher antibacterial activities, broader antibacterial spectrums and fewer possibilities resulting in target strains’ resistance mutation [4]. Therefore, AMPs have a wide range of application prospects in the pharmaceutical industry and have become a hotspot in biomedical research [4].

Based on existing research data about AMPs, many efforts have been made to construct various databases containing experimentally validated AMPs. To date, a number of databases have been developed to provide comprehensive experimentally verified annotations of AMPs. For example, the antimicrobial peptide database (APD3) [5] includes various AMPs with different functional activities such as antibacterial, antifungal, antiviral and anticancer. It also provides a user-friendly web page for peptide classification, search and prediction. dbAMP [6, 7] is another comprehensive AMP database specifically focusing on AMPs’ functional and physicochemical characteristics in high-throughput transcriptome and proteome data. It also provides relevant annotations of AMP–protein interactions and targeting species of AMPs. DRAMP [8–10] offers access to minimum inhibitory concentration values and structure information of AMPs, and LAMP [11, 12] crosslinks existing AMP databases into account and provides the related information. In addition to comprehensive databases, some disease-specific databases integrate AMPs with specific functional activities, such as AntiTbPdb Field [13], a database for antitubercular (anti-TB) peptides. With the continuous expansion and development of AMP databases, identifying AMPs and their functional types accurately by computational methods has become increasingly important for AMP research, due to the time-consuming, expensive and laborious wet-laboratory experiments.

In the last decade, a plethora of computational predictors have been developed for identifying AMPs [13–16]; a few attempts have also been made to review, benchmark and evaluate these approaches [17, 18]. However, the majority of these predictors only focus on identifying AMPs. As such, they cannot be used to predict the functional activities of particular interest to biomedical researchers. Several predictors have been proposed to predict AMPs with one specific functional activity, such as DeepAVP for predicting antiviral peptides [19], Deep-AFPpred for predicting antiviral peptides [20], and StaBle-ABPpred [21] and Deep-ABPpred [22] for predicting antibacterial peptides. However, these predictors have limited predictive capability and cannot provide comprehensive functional activity annotations of AMPs. Furthermore, only a few predictors can predict multiple functional activities of AMPs, given the significance of AMP functional activities and the fact that none of the work systematically summarized and evaluated these computational approaches for predicting AMPs and their functional activities. Herein, we reviewed these computational approaches comprehensively, including the involved functional activities, benchmark datasets, machine learning algorithms, feature selection algorithms, and performance evaluation strategies and metrics. Then, we developed a predictive framework named iAMPCN (identification of AMPs and their functional activities based on convolutional neural networks), which is composed of multiple single-class models. We evaluated its ability to identify different kinds of functional activities of AMPs. The performance evaluation results demonstrated that iAMPCN achieved superior performances in identifying AMPs and their functional types compared with available predictive tools. We anticipate that iAMPCN can serve as a prominent tool for identifying potential AMPs and their specific functions that can be experimentally validated.

## MATERIALS AND METHODS

### Existing approaches for predicting AMPs and their functional types

**Figure 1:**
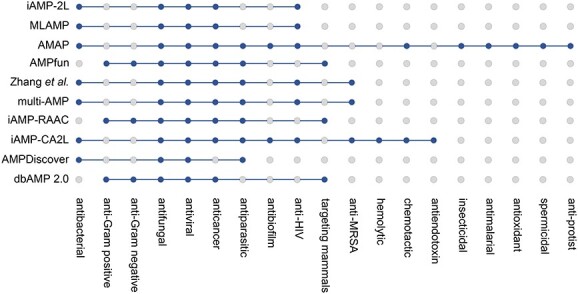
A summary of current computational approaches for predicting AMPs and their functional activities. The blue dots indicate the functional activities that the predictors can predict.

In the present study, we comprehensively reviewed 10 computational predictors for AMPs and their activities regarding the predicted functional activities, data sources, algorithms and performance evaluation strategies in Table 1 and Figure 1. We also provided a summary of the key features used in these approaches in Table 2. We grouped these tools into traditional machine learning (ML)-based and deep learning (DL)-based according to the algorithms they applied.

**Table 1:** A comprehensive summary of the reviewed approaches for AMP prediction

Category	Tool	Year	Prediction of functional activities	Positive data source	CD-HIT cut-off	Algorithm	Sampling strategy	Feature selection	Evaluation strategy	Evaluation metrics	Webserver/software availability
Machine learning based	iAMP-2L	2013	Antibacterial, anticancer, antiviral, antifungal, anti-HIV	APD2	1st: 40%; 2nd: 40%	FKNN	N.A.	N.A.	Jack-knife validation test, independent test	*Acc*, *Sp*, *Sn*, MCC, macro *Pr*, macro *Sn*, macro *Acc*, SA, HL	Yes
	MLAMP	2016	Antibacterial, anticancer, antifungal, anti-HIV, antiviral	APD2	1st: 40%; 2nd: 40%	RF, ECC	SMOTE	N.A.	Jack-knife validation test, independent test	*Acc*, *Sp*, *Sn*, MCC, macro *Pr*, macro *Sn*, macro *Acc*, SA, HL	Yes
	AMAP	2019	Antibacterial, anticancer, antifungal, anti-HIV, antiviral, antibiofilm, antiparasitic, chemotactic, insecticidal, antimalarial, antioxidant, spermicidal, anti-protist	APD3	1st: N.A.; 2nd: N.A.	SVM, XGB, one-vs-rest classifier fusion	N.A.	N.A.	LOCO, 5-fold CV, independent test	AUC, AUPR, MCC	Yes
	AMPfun	2019	Antiparasitic, anticancer, antifungal, antiviral, targeting mammals, targeting Gram-positive bacteria, targeting Gram-positive bacteria	APD3, ADAM, ParaPep, AVPdb, CancerPPD, MLACP, AntiCP, AntiFP, DRAMP	1st: 50%; 2nd: N.A.	DT, RF, SVM	N.A.	SFS	10-fold CV, independent test	*Acc*, *Sp*, *Sn*, MCC, AUC	Yes
	Zhang *et al.*’ s work	2020	Anti-MRSA, antibacterial, antiviral, antifungal, anticancer, anti-HIV, antiparasitic	APD3, APD2	1st: 40%; 2nd: 40%	GBDT, ET, ECC	ADASYN	Lasso	10-fold CV, independent test	*Acc*, *Sp*, *Sn*, MCC, macro *Pr*, macro *Sn*, macro *Acc*, HL, AP	No
	iAMP-RAAC	2021	Antiparasitic, anticancer, antifungal, antiviral, targeting mammals, targeting Gram-positive bacteria, targeting Gram-positive bacteria	APD3, ADAM, ParaPep, AVPdb, CancerPPD, MLACP, AntiCP, AntiFP, DRAMP	1st: 50%; 2nd: N.A.	SVM	N.A.	ANOVA, IFS	10-fold CV，independent test	*Acc*, *Sp*, *Sn*, MCC	Yes
	dbAMP 2.0	2021	Anticancer, antifungal, antiviral, targeting mammals, targeting Gram-positive bacteria, targeting Gram-positive bacteria	dbAMP 2.0, APD2, CAMP	1st: 50%; 2nd: N.A.	GBDT	N.A.	N.A.	5-fold CV	*Sn*, *Sp*, balanced *Acc*	Yes
Deep learning based	iAMP-CA2L	2021	Antibacterial, anticancer, antifungal, anti-HIV, antiviral, antibiofilm, antiparasitic, chemotactic, anti-MRSA, antiendotoxin	APD3, AMPer, ADAM	1st: 90%; 2nd: 90%	DL	N.A.	N.A.	Jack-knife validation, independent test	*Acc*, *Pr*, *Sp*, *Sn*, MCC, F1, SA, AP, coverage, HL, RL, OE, macro *Pr*, macro *Sn*, macro F1, micro *Pr*, micro *Sn*, micro F1	Yes
	Multi-AMP	2021	Anti-MRSA, antibacterial, antiviral, antifungal, anticancer, anti-HIV, antiparasitic	APD3, APD2	1st: 40%; 2nd: 40%	DL	N.A.	N.A.	5-fold CV, independent test	*Acc*, *Sp*, *Sn*, MCC, F1, macro *Sp*, macro *Sn*, macro *Acc*, HL, macro MCC, macro F1	No
	AMPDiscover	2021	Antibacterial, antifungal, antiviral, antiparasitic	starPepDB	1st: 50%; 2nd: N.A.	RF, DL	N.A.	Correlation subset, SE, Relif-F, IG, gain ratio, symmetrical uncertainty	5-fold CV, 10-fold CV, 15-fold CV, 20-fold CV, 25-fold CV, independent test	*Acc*, *Sp*, *Sn*, MCC, AUC	Yes

**Table 2:** Different types of features employed by the reviewed approaches for AMP prediction

Feature type	Feature	Tools
Composition features	Amino acid composition (AAC)	AMAP, AMPfun, dbAMP 2.0
	Dipeptide composition (DPC)	dbAMP 2.0
	N-gram composition found by counting (NCC)	AMPfun
	N-gram composition found by *t*-test (NTC)	AMPfun
	Motifs composition (MC)	AMPfun
Position features	N-gram binary profiling of position found by counting (NCB)	AMPfun
	N-gram binary profiling of position found by *t*-test (NTB)	AMPfun
	Motifs binary profiling of position (MB)	AMPfun
Structural and physicochemical properties	Physicochemical properties	dbAMP 2.0
	Composition/transition/distribution (CTD)	AMPfun
	Pseudo amino acid composition (PseAAC)	iAMP-2L, MLAMP, AMPfun, dbAMP 2.0
	Conjoint triad (CTriad)	AMAP
	Reduced amino acid clusters (RAAC)	iAMP-RAAC
	Protein Descriptors Calculation (ProtDCal)	AMPDiscover
Evolutionary information	Cellular automata images (CAIs)	iAMP-CA2L

#### Machine learning-based approaches

To our best knowledge, iAMP-2L [23] is the first AMP predictor, which utilizes the fuzzy K-nearest neighbor (FKNN) algorithm to identify both AMPs and their functional activities, including antibacterial, anticancer, antiviral, antifungal and anti-human immunodeficiency virus (anti-HIV). The first-level prediction of iAMP-2L is to determine whether a peptide is an AMP, for which the FKNN [24] algorithm was utilized. The second-level prediction of iAMP-2L was designed to characterize the functional activity types of the queryAMP, for which the multi-label fuzzy K-nearest neighbor (ML-FKNN) classifier was applied. Both levels of prediction utilized the pseudo amino acid composition (PseAAC) [25] to represent sequences. In another work, Lin *et al*. proposed MLAMP [26] by mainly focusing on addressing the unbalanced labeled problem in the two-level AMP prediction. ML-SMOTE was developed by modifying the synthetic minority oversampling technique (SMOTE) [27] based on the multi-label classification. In addition, MLAMP converted the PseAAC [25] into new vectors as sequence representations with the grey model [28]. Another approach utilizing oversampling technique was proposed by Zhang *et al.* [29], which employed adaptive synthetic sampling (ADASYN) [30] to handle the data imbalance issue. Both MLAMP and Zhang *et al.*’s work applied the ensemble classifier chain (ECC) [31] to classify the AMP functional activities where the relationship between labels was considered. The only difference is that Zhang *et al.* utilized the gradient boosting decision tree (GBDT) [32] as the classifier for the first stage of prediction and the extra tree (ET) [33] for the second stage. In contrast, both stages of MLAMP applied the random forest (RF) as the classifier. Besides, Zhang *et al.* adopted Lasso [34] for feature selection to further improve the performance. AMAP [35] utilized the support vector machine (SVM) [36] and extreme gradient boosting (XGBoost) [37] as the classifiers, trained with amino acid composition (AAC) [38] and physicochemical properties to identify AMPs. Compared with other predictors, AMAP can identify the most functional types. AMPfun [39] is also based on RF but was built on a more comprehensive training dataset by integrating multiple AMP databases. AMPfun employed abundant sequence-based features, including compositional information, physicochemical descriptions and binary profiles, for model training. It can classify a range of functional activities, including antiparasitic, antiviral, anticancer, antifungal and anti-mammalian cells, anti-Gram positive and anti-Gram negative. iAMP-RAAC [40] is also a two-stage AMP predictor based on SVM and reduced amino acid cluster (RAAC) [41] features. iAMP-RAAC was trained on the same datasets as AMPfun and can predict the same functional activities as AMPfun. In addition, dbAMP 2.0 [6], an AMP database, has a user interface developed based on the GBDT algorithm for identifying AMPs and their functional types.

#### Deep learning-based approaches

With the advances and applications of DL techniques in bioinformatics, several DL-based predictors have been recently developed. Among them, iAMP-CA2L [42] is the first DL-based predictor for AMPs and their functional activities. iAMP-CA2L used the ANTIALIAS [43] technology to extract features from cellular automata images (CAIs) [44] of sequences and convolutional neural networks (CNNs) [45] and long short-term memory (LSTM) [46] to further extract features. Then, two SVMs were trained with these features to distinguish AMPs from non-AMPs and annotate 10 different functional activities, including antibacterial, antiviral, antifungal, antibiofilm, antiparasitic, anti-HIV, anticancer, chemotactic, anti-Methicillin-resistant Staphylococcus aureus (anti-MRSA) and antiendotoxin. Another recent approach, multi-AMP [47], has also been developed based on DL. However, different from iAMP-CA2L, multi-AMP considered the AMP identification as a multi-task problem. Accordingly, the final output layers of multi-AMP are used to address two tasks: AMP identification and their functional activity prediction. The models for these tasks shared identical parameters in the front layers (CNNs) for feature extraction but were structured with different dense layers for the final prediction. Multi-AMP used the position-specific scoring matrix [48] to represent peptide sequences. AMPDiscover [49] is another recently developed webserver, which provides two predictive models for AMPs: RF models with six different feature selection strategies, and the RNNs (recurrent neural networks) [50].

#### Sequence similarity threshold

To remove the redundant sequences from the training datasets to avoid the overfitting issue during training machine learning models, most studies employed CD-HIT [51–53] with a relatively stringent sequence similarity threshold to reduce the sequence redundancy. For example, iAMP-2L [23] and Zhang *et al*.’s work [29] set the sequence identity threshold at 40%. AMPfun [39], dbAMP 2.0 [6] and AMPDiscover [49] set the sequence identity threshold at 50%. However, in the case of functional activity prediction of AMPs, most studies set a relatively loosened threshold or did not remove redundant sequences, which is probably because of the rather limited annotations of functional activities. For example, iAMP-CA2L [42] set the sequence identity threshold at 90%, while AMAP [35], AMPfun [39], dbAMP 2.0 [6] and AMPDiscover [49] did not remove the sequence redundancy.

### Model development

Although several approaches have been proposed to characterize AMPs and their functional activities, they have some drawbacks or limitations. First, the majority of the existing approaches only focused on limited functional activities. Although other approaches can cover more functional types, they were developed based on relatively out-of-date databases with fewer AMPs, resulting in the inaccurate annotation of the functional activities. AMAP can predict several activities that other methods cannot, such as antibiofilm, insecticidal, antimalarial, antioxidant, spermicidal and anti-protist. However, its models for identifying these functional activities were trained with less than 50 positive samples, which was particularly the case that only four positive samples were used to identify the ‘anti-protist’ activity of AMPs. Accordingly, we assume that the predictive results of these models could be less reliable, given the fact that many publicly available AMP databases have been updated recently. Second, different AMP databases may have some differences in AMPs that they include. Nevertheless, most existing AMP predictors were developed based on the data collected from one or only a few databases. As a result, the training datasets on which these predictors were built could be biased and poorly annotated. In view of these shortcomings, it is necessary to curate a comprehensive training dataset by integrating the available databases and developing more accurate predictors for identifying AMPs and their functional activities.

In this study, we introduce a two-stage computational framework, termed identification of AMPs and their functional activities based on Convolutional Neural networks (iAMPCN), to address the aforementioned shortcomings and improve the predictive performance of AMPs and their functional activities. Specifically, iAMPCN employs the one-hot encoding scheme to encode the peptide sequences and a two-stage framework coupled with CNN to train the model and conduct the prediction.

#### Dataset construction

iAMPCN is a two-stage computational framework for identifying AMPs and their functional types. Accordingly, we constructed comprehensive benchmark training and independent test datasets for both tasks. Our detailed procedures are described below.

In the first stage, for the positive dataset, we collected the AMPs with functional activity annotations from several databases, including APD3 [5], dbAMP [6, 7], DRAMP [8–10], dbaasp [54–56], CAMP [57, 58], LAMP [11, 12], ParaPep [59], phytAMP [60], AVPdb [61], CancerPPD [62], AntiTbPdb [63], Hemolytik [64] and milkAMP [65], as well as the training datasets of 24 AMP classifiers, including ADAM [66], iAMP-2L [23], AMPfun [39], iAMP-CA2L [42], MLACP [67], AntiCP 2.0 [68], ACPred [69], ACPred-FL [70], ACPred-Fuse [71], ACP-DL [72], iACP-DRLF [73], mACPpred [74], AntiFP [75], AVPpred [76], AVPIden [77], AntiTbPred [78], AtbPpred [78], BIOFIN [79], BIPEP [80], dPABBs [81], HemoPI [82], HLPpred-Fuse [83], iAntiTB [84], AnOxPePred [85] and HAPPENN [86]. Then, we eliminated those sequences with non-standard residues such as ‘B’, ‘J’, ‘O’, ‘U’, ‘X’ or ‘Z’. Finally, 49 115 experimentally validated AMP sequences were extracted from these resources. We performed the following procedures to generate the negative dataset: (i) peptide sequences were downloaded from UniProt [87–90] (http://www.uniprot.org), and all entries containing the keyword ‘antimicrobial’ and related keywords (e.g. ‘antibacterial’, ‘antifungal’, ‘anticancer’, ‘antiviral’, ‘antiparasitic’, ‘anticancer’, ‘antibiotic’, ‘antibiofilm’ or ‘effector’) were removed; (ii) the sequences with the length greater than 200 or less than 10 amino acid residues and with non-standard residues ‘B’, ‘J’, ‘O’, ‘U’, ‘X’ or ‘Z’ were discarded; (iii) the CD-HIT program [51–53] was applied to remove the sequences that had 40% pairwise sequence identity to those in the positive dataset. Finally, 195 525 negative samples were obtained.

Here, we applied the same strategy as AMPfun [39] to construct the training and independent test datasets for each functional type. We collected positive and negative samples from 49 115 experimentally validated AMP sequences for each functional activity. The authors of AMPfun [39] and iAMP-CA2L [42] also constructed a relatively large AMP dataset from several different databases. As mentioned in AMPfun [39], a peptide with a specific functional activity would be used as a positive sample of this activity; otherwise, it would be treated as a negative sample. We integrated the positive and negative datasets for 22 different functional types in the same way. It is known that the dataset containing instances with missing labels is an unavoidable problem. Even though all AMP sequences are obtained from only one database, it is very likely that these sequences need to be completely annotated since different AMP databases have been updated several times. In addition, there are a large number of overlapping AMPs between different AMP databases, and the linking antimicrobial peptides (LAMP) [11, 12] database was constructed to reflect the crosslink between several AMP databases [11, 12]. Therefore, combining peptides from multiple databases can make the activity annotations more complete. Irrespective of the combinations or not, instances with missing labels must exist. Because of the large number of overlapping AMPs, combining peptides from multiple datasets will be better than using peptides from only one dataset.

A detailed summary of positive and negative datasets for the first and second stages is shown in Table 3. For training and evaluating predictive models for AMP and functional activity predictions, we randomly selected 20% samples from positive and negative datasets to construct test datasets. We also used the remaining 80% of the samples as training datasets. We then filtered those sequences used in the training datasets of other predictive tools and split the remaining datasets to construct the independent datasets and training datasets. Detailed information on training, test and independent test datasets is shown in Supplementary Tables S1 and S2.

**Table 3:** The numbers of positive and negative samples for predicting AMP and their functional activities

	Positive samples	Negative samples
AMPs	49 115	195 525
Functional activity		
Antibacterial	16 058	13 551
Antibiofilm	372	29 202
Anticancer	4698	24 876
Anticandidal	669	28 905
Antifungal	5955	23 619
Anti-Gram negative	8537	21 044
Anti-Gram positive	8053	21 539
Anti-HIV	812	28 762
Antimalarial	73	29 501
Anti-MRSA	267	29 307
Antiparasitic	457	29 117
Antiplasmodial	63	29 511
Antiprotozoal	53	29 521
Anti-TB	275	29 299
Antiviral	6495	23 093
Anti-mammalian cells	4345	25 229
Anuran defense	779	28 795
Chemotactic	85	29 489
Cytotoxic	180	29 394
Endotoxin	82	29 492
Hemolytic	2383	27 191
Insecticidal	415	29 159

#### Model construction

**Figure 2:**
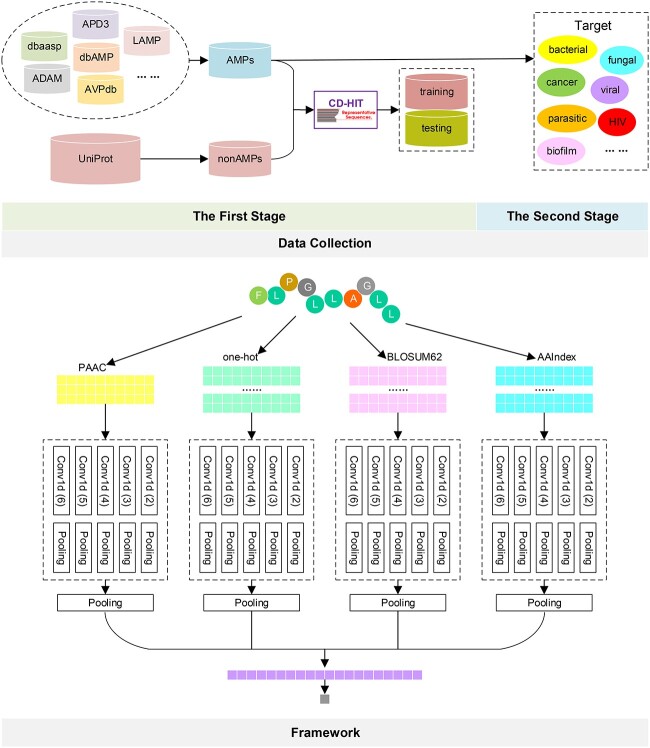
The architecture of iAMPCN. Four kinds of sequence representations were utilized, and different CNN models with different filter lengths ranging from two to six were utilized to extract feature information of varying sequence lengths from each type of encodings.

**Figure 3:**
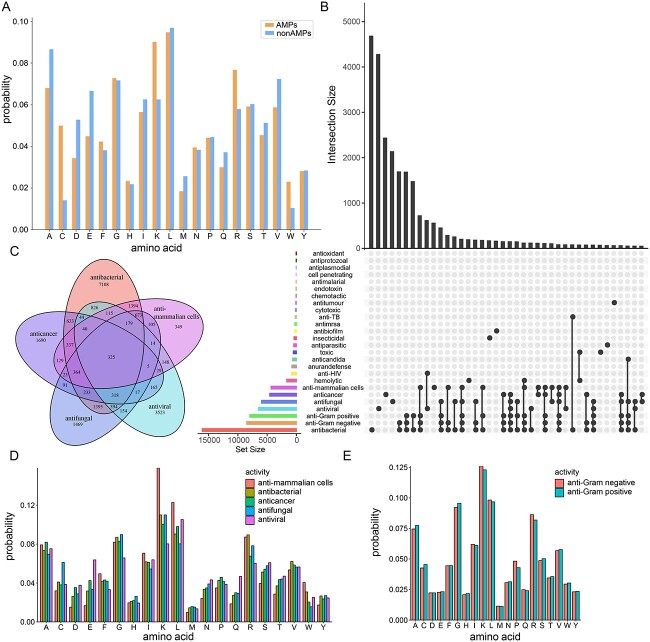
Sequence analysis of AMPs collected for this study. (**A**) Amino acid distributions of AMP sequences versus non-AMP sequences. (**B**) An UpSet plot demonstrating the statistics of AMPs with overlapping and/or unique functional activities. (**C**) A Venn diagram illustrating the distributions of AMPs with five main functional activities, including antibacterial, anticancer, antifungal, antiviral and anti-mammalian cells. (**D**) Amino acid distributions of the AMPs with the five major functional activities. (**E**) Amino acid distributions of anti-Gram-positive and anti-Gram-negative AMPs.

An overview of the architecture of iAMPCN is illustrated in Figures 2 and 3. Here, we utilized four different types of amino acid information to represent peptide sequences, including one-hot encoding, BLOSUM62 encoding, AAIndex [91, 92] encoding and PAAC encoding. When using the one-hot encoding scheme, a peptide sequence is converted into a numerical matrix with the dimension of 200^*^21, where 200 represents the length of a peptide sequence and a 21-dimensional binary vector represents each amino acid type. For example, the one-hot encoding of A is [1,0,0,0,0,0,0,0,0,0,0,0,0,0,0,0,0,0,0,0,0], C is [0,1,0,0,0,0,0,0,0,0,0,0,0,0,0,0,0,0,0,0,0] and the one-hot encoding of the non-standard amino acids is [0,0,0,0,0,0,0,0,0,0,0,0,0,0,0,0,0, 0,0,0,1]. The model with one-hot vector encoding with 21 bits and the model with one-hot vector encoding with 20 bits are the same when training and testing the sequences with usual amino acids (a detailed analysis is provided in the Supplementary Methods). We padded the vectors with ‘0’ for those sequences shorter than 200 and removed parts of sequences for those longer than 200 to obtain fixed dimensional matrices. For BLOSUM62 encoding, a peptide sequence is converted into a numerical matrix with the dimension of 200^*^23, where 200 again represents the length of a peptide sequence and a 23-dimensional vector represents each amino acid type. This encoding reflects the evolutionary information of amino acid residues. For the AAIndex encoding, a peptide sequence is converted into a numerical matrix with the dimension of 200^*^531. Each amino acid type is represented by a 531-dimensional vector that presents various physicochemical and biochemical properties of amino acids. Lastly, for the PAAC encoding, a peptide sequence is converted into a numerical matrix with a dimension of 200^*^3. Each amino acid type is represented by a three-dimensional vector that indicates the original hydrophobicity, hydrophilicity and side-chain masses of amino acid residues. Recently, Otović *et al*. [93] proposed a model based on the recursive neural network (RNN) to predict therapeutic peptides. This model also used physicochemical encodings to represent sequences. Without using the AAIndex [91, 92], it used the ‘AAdata’ from the R package ‘Peptides’ [94]. Each amino acid of ‘AAdata’ has 94 physicochemical properties that reflect hydrophobicity, electronegativity, alpha and turn propensities, etc. Most of these physicochemical properties were calculated using some formulas, such as principal components and Z-scale. Compared with ‘AAdata’, ‘AAIndex’ contains more enriched and detailed physicochemical properties. For example, ‘AAIndex’ includes the ‘short and medium range non-bonded energy per residue’, which are not included in ‘AAdata’. In addition, ‘AAIndex’ also provides other original, informative physicochemical properties.

The one-dimensional CNNs (i.e. Conv1d) with different filter lengths ranging from two to six were utilized to extract feature information of varying sequence lengths from each type of encoding. First, the features extracted by each CNN were normalized and inputted into a pooling layer. Then, the extracted features from the same encoding type were combined and inputted into a pooling layer to obtain the final features. After combining these features, a final dense layer was used to give the final predictive output. The parameters of this framework are described in detail in the Supplementary Methods.

In this study, we utilized the transfer learning strategy—specifically, we applied the model for predicting AMPs and non-AMPs trained at the first stage of AMP prediction, as the pretrained model to initialize all parameters’ weights of the models for predicting functional activities. The learning rates of model training for both the AMP prediction and AMP functional activity prediction were set as the same, i.e. 0.0001. Due to the imbalanced datasets of certain functional activities, the focal loss [95] was applied as follows:


$$ FL\left({p}_t\right)=-{a}_t{\left(1-{p}_t\right)}^{\gamma}\log \left({p}_t\right), $$

where ${a}_t$ controls the weights of the samples belonging to positive and negative classes, and ${p}_t$ is an estimated probability by the model, ${\left(1-{p}_t\right)}^{\gamma }$ is considered to be a modulating factor that controls the effects of easy-classified samples. Here, ${a}_t$ and $\gamma$ were set to 1 and 2, respectively. In addition, the early stopping strategy was applied during the 10-fold stratified cross-validation test to avoid overfitting.

#### Performance evaluation strategies and metrics

Performance evaluation strategies, including the *K*-fold stratified cross-validation test, jack-knife validation test and independent test, are commonly used to evaluate predictors and optimize the parameters of models. In this study, we employed the 10-fold stratified cross-validation test on training data sets for model selection and optimization. In addition, we used the independent test to compare the predictive performance of different tools. To objectively assess and compare the model’s performance with different tools and web servers, six performance metrics were used, including sensitivity, specificity, precision, accuracy, Matthew’s correlation coefficient (MCC) [96] and area under the ROC curve (AUROC or AUC), which are defined as follows:


$$ \left\{\begin{array}{@{}ll} Sensitivity=\frac{TP\ }{TP+ FN}&\ 0\le Sn\le 1\\ Specificity=\frac{TN}{TN+ FP}& \ \ 0\le Sp\le 1\\ Precision=\frac{TP}{TP+ FP}& \ \ 0\le \mathit{\Pr}\le 1\\ Accuracy=\frac{TP+ TN}{TP+ FN+ TN+ FP}& \ 0\le Acc\le 1\\ MCC=\frac{TP\times TN- FP\times FN}{\sqrt{\left( TP+ FP\right)\ \left( TP+ FN\right)\ \left( TN+ FP\right)\ \left( TN+ FN\right)\ }}\ & -1\le MCC\le 1\end{array}\right. $$

where *TP* and *TN* represent the numbers of correctly predicted positive and negative samples, respectively, while *FP* and *FN* represent the numbers of incorrectly predicted positive and negative samples, respectively.

## RESULTS AND DISCUSSION

### Sequence analysis of collected AMPs

The amino acid distributions of AMPs and non-AMPs in the constructed datasets are shown in Figure 3A. Compared with non-AMPs, cysteine (C), lysine (K) and arginine (R) residues appeared to be abundant in AMP sequences. This observation is consistent with previous studies, which show that most active AMP sequences are abundant in hydrophilic or positively charged amino acids [3, 97]. An overview of the extracted functional activities of the AMP sequences is shown in Figure 3B–C. We compared the amino acid distributions of AMPs in terms of five main functional activities (Figure 3D). We found that the AMPs with the activity of targeting mammalian cells have more lysine (K) and leucine (L) residues, and the AMPs with antifungal activity have more cysteine residues. We also compared the amino acid distributions of AMPs with anti-Gram-negative and anti-Gram-positive activity, given that most AMPs with anti-Gram-negative activity can also usually perform the anti-Gram-positive activity (Figure 3B). We found that the amino acids of these two types of AMPs have similar compositions (Figure 3E). The detailed amino acid distributions of the other activities are shown in Supplementary Figure S1. In addition, we compared the length distributions of AMPs and non-AMPs (Supplementary Figure 2), as well as the length distributions of positive samples and negative samples of different functional activity datasets (Supplementary Figure 3). As a result, we noticed no significant length differences between the AMPs with various activities.

### Predictive performance for AMP identification

**Figure 4:**
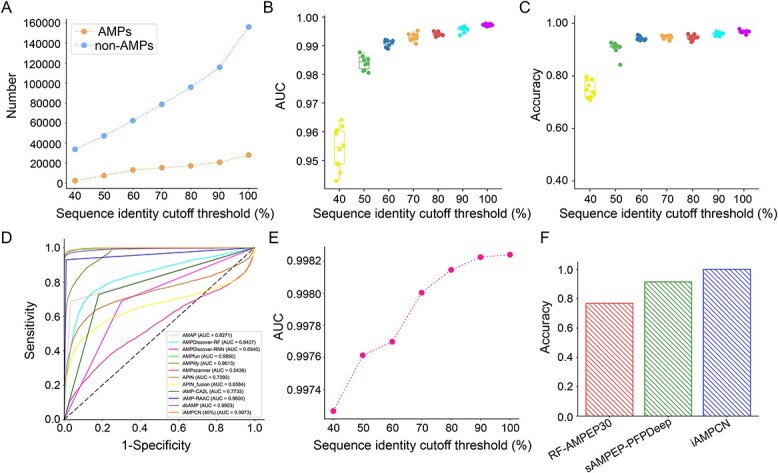
Performances of AMP prediction. (**A**) The numbers of AMPs and non-AMPs in the training datasets with different sequence identity cut-off thresholds. (**B**–**C**) AUCs and accuracy values of iAMPCN built on the training datasets with different sequence identity cut-off thresholds based on the 10-fold stratified cross-validation test. (**D**) ROC curves and the corresponding AUC values of various modes using the independent test dataset. (**E**) AUCs of iAMPCN built on the training datasets with different identity thresholds via the independent test dataset. (**F**) The accuracy values of identifying short AMPs (5–30 AAs) on the independent test dataset.

**Figure 5:**
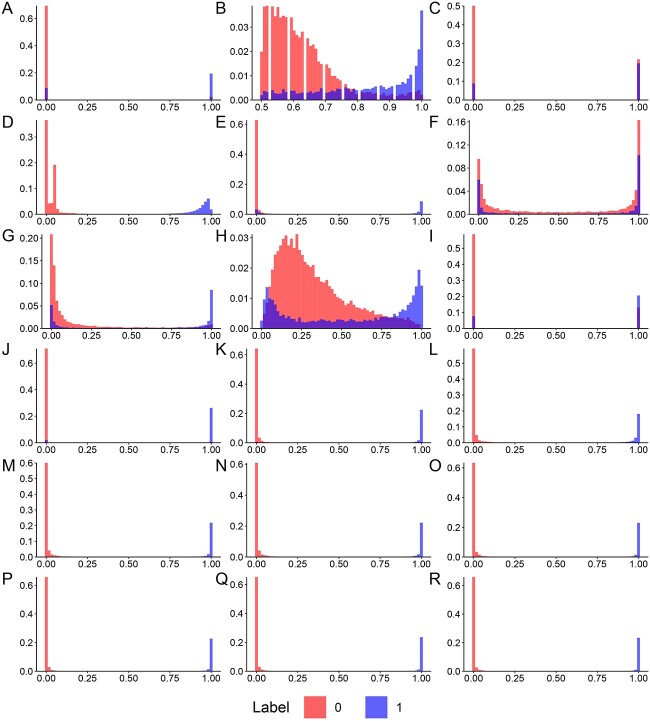
Predictive probability distributions of different computational approaches based on the independent test dataset. These approaches include (**A**) AMAP, (**B**) AMPDiscover-RF, (**C**) AMPDiscover-RNN, (**D**) AMPfun, (**E**) AMPlify, (**F**) AMPscanner, (**G**) APIN, (**H**) APIN-fusion, (**I**) iAMP-CA2L, (**J**) iAMP-RAAC, (**K**) dbAMP, (**L**) iAMPCN (CD-HIT 40%), (**M**) iAMPCN (CD-HIT 50%), (**N**) iAMPCN (CD-HIT 60%), (**O**) iAMPCN (CD-HIT 70%), (**P**) iAMPCN (CD-HIT 80%), (**Q**) iAMPCN (CD-HIT 90%) and (**R**) iAMPCN (CD-HIT 100%).

Most studies usually utilized sequence cluster programs, such as CD-HIT, to remove highly similar sequences from the training dataset to reduce homology bias and redundancy. However, some studies indicated that this step would affect the predictive performance of the trained model. Therefore, we first evaluated the predictive performance of the models using training datasets with different sequence identity cut-offs ranging from 40 to 100%. Figure 4A shows the numbers of positive and negative samples of the training datasets with varying thresholds of identity, and the performance evaluation results based on 10-fold stratified cross-validation tests on these training datasets are shown in Figure 4B–C and Table 4. The performance comparison results indicate that the AUC and accuracy of the model trained on a dataset pre-processed with a higher identity cut-off are better than that with a lower identity cut-off value. In addition, we compared our model with an identity cut-off of 40% with several available webserver/tools, including AMAP [35], AMPDiscover [49], AMPfun [39], AMPlify [98], AMPscanner [15], APIN [16], iAMP-CA2L [42], iAMP-RAAC [40] and dbAMP [6], using the independent test dataset. Furthermore, we compared the performance of our model trained by training datasets with different identity cut-off thresholds ranging from 40 to 100% on the independent test dataset and provided the results in Figure 4D–E. iAMPCN trained on the dataset with an identity cut-off of 40% achieved the AUC value of 0.9973, outperforming other approaches. Along with the identity thresholds increasing, the AUC values increased slightly. We also evaluated the predictive probability distributions and performance metrics of different computational approaches on the independent validation dataset and demonstrated the results in Figure 5 and Table 5. These distributions also suggest that the iAMPCN has much better prediction performance. Moreover, we selected those AMPs with lengths ranging from 5 to 30 amino acids from the independent test dataset to construct a new test dataset to further evaluate the performance of iAMPCN in identifying short AMPs. The performance comparison results in terms of the accuracy of iAMPCN and two state-of-the-art approaches for predicting short AMPs, RF-AMPEP30 [99] and sAMPEP-PFPDeep [100], are provided in Figure 4F. All these performance evaluation results demonstrated that iAMPCN achieved a highly competitive predictive performance and outperformed state-of-the-art predictors for AMP identification.

**Table 4:** Performances of 10-fold cross-validation test based on the AMP training dataset

Sequence identity	Sensitivity	Specificity	Accuracy	MCC	AUC
40%	0.5065 ($\pm$0.0648)	0.9929 ($\pm$0.0032)	0.7497 ($\pm$0.0309)	0.6312 ($\pm$0.0276)	0.9541 ($\pm$0.0070)
50%	0.8265 ($\pm$0.0493)	0.9874 ($\pm$0.0048)	0.9070 ($\pm$0.0227)	0.8492 ($\pm$0.0191)	0.9841 ($\pm$0.0023)
60%	0.8960 ($\pm$0.0145)	0.9890 ($\pm$0.0031)	0.9425 ($\pm$0.0065)	0.9040 ($\pm$0.0085)	0.9910 ($\pm$0.0011)
70%	0.9034 ($\pm$0.0158)	0.9914 ($\pm$0.0026)	0.9474 ($\pm$0.0068)	0.9147 ($\pm$0.0053)	0.9930 ($\pm$0.0012)
80%	0.8972 ($\pm$0.0207)	0.9942 ($\pm$0.0022)	0.9457 ($\pm$0.0094)	0.9187 ($\pm$0.0071)	0.9941 ($\pm$0.0006)
90%	0.9270 ($\pm$0.0122)	0.9942 ($\pm$0.0011)	0.9606 ($\pm$0.0060)	0.9370 ($\pm$0.0075)	0.9958 ($\pm$0.0009)
100%	0.9409 ($\pm$0.0124)	0.9961 ($\pm$0.0021)	0.9685 ($\pm$0.0053)	0.9518 ($\pm$0.0032)	0.9972 ($\pm$0.0003)

**Table 5:** Performances of different predictors based on the AMP independent test dataset

Tool	Precision	Sensitivity	Specificity	Accuracy	F1	MCC	AUC
AMAP	0.8908	0.6875	0.9668	0.8271	0.776	0.7134	0.8271
AMPDiscover-RF	0.3671	0.6882	0.5326	0.6104	0.4788	0.1992	0.8407
AMPDiscover-RNN	0.4740	0.6894	0.6986	0.6940	0.5617	0.3551	0.6940
AMPfun	0.9784	0.9687	0.9916	0.9801	0.9735	0.9632	0.9950
AMPlify	0.9611	0.6162	0.9902	0.8032	0.7510	0.7089	0.9613
AMPscanner	0.3045	0.5885	0.4707	0.5296	0.4014	0.0535	0.5436
APIN	0.6618	0.5868	0.8819	0.7343	0.6220	0.4870	0.7393
APIN_fusion	0.5229	0.5585	0.7993	0.6789	0.5401	0.3509	0.6584
iAMP-CA2L	0.6123	0.7283	0.8184	0.7733	0.6653	0.5211	0.7733
iAMP-RAAC	0.9736	0.9299	0.9901	0.9600	0.9513	0.9332	0.9600
dbAMP	0.9835	0.9473	0.9937	0.9705	0.9650	0.9519	0.9923
iAMPCN (40%)	0.9809	1.0000	0.9923	0.9962	0.9904	0.9866	0.9973
iAMPCN (50%)	0.9869	1.0000	0.9948	0.9974	0.9934	0.9908	0.9976
iAMPCN (60%)	0.9892	1.0000	0.9957	0.9978	0.9946	0.9924	0.9977
iAMPCN (70%)	0.9922	1.0000	0.9969	0.9985	0.9961	0.9946	0.9980
iAMPCN (80%)	0.9957	1.0000	0.9983	0.9992	0.9979	0.9970	0.9981
iAMPCN (90%)	0.9943	1.0000	0.9978	0.9989	0.9972	0.9960	0.9982
iAMPCN (100%)	0.9969	1.0000	0.9988	0.9994	0.9985	0.9979	0.9982

### Performance on identifying functional activities of AMPs

To preliminarily assess the performance of iAMPCN using the extracted features, we evaluated the predictive performances of identifying functional activities based on three existing datasets constructed by previous studies [23, 39, 42] and provided the performance comparison results in Supplementary Tables S3–S5. The results indicate that the models built on the combination of four types of sequence features usually outperformed those models based on one type of sequence representation. Furthermore, on the iAMP-2L benchmark dataset, iAMPCN achieved the best performance in terms of all performance measures except hamming loss, which might be caused by the small dataset. Besides, iAMPCN performed best on the iAMP-CA2L benchmark dataset in terms of accuracy. These results indicate that iAMPCN is a competitive predictor for AMPs and their functional activities.

To explore the effects of different sequence identity thresholds in predicting functional activities, we also evaluated the predictive models trained on the benchmark datasets with different CD-HIT thresholds ranging from 40 to 100% (Supplementary Tables S6–S27, Supplementary Figure S4). For most functional activities, the higher the threshold of sequence identity, the higher the accuracy and robustness of the models achieved. Therefore, we utilized the trained models based on the training datasets with a CD-HIT threshold of 100% for the rest of the analysis.

#### Performance comparison of AMP function prediction on the independent test datasets

**Figure 6:**
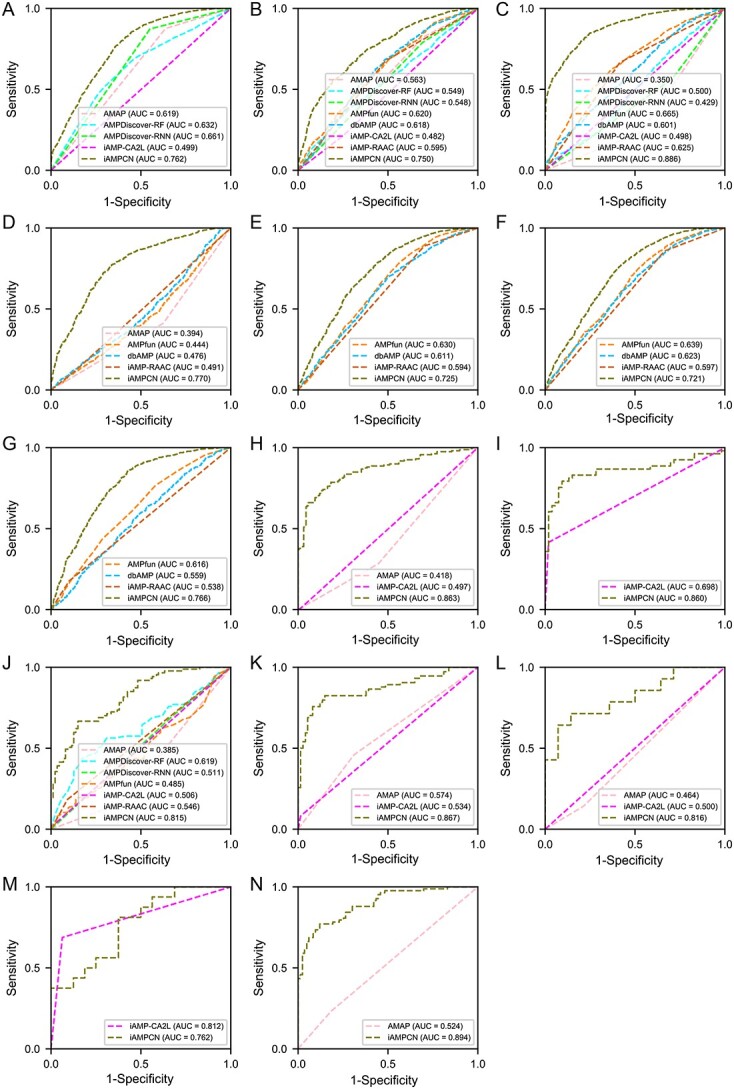
ROC curves and the corresponding AUC values of different tools for predicting 14 AMP functional activities on independent test datasets. These functional activities are (**A**) antibacterial, (**B**) antifungal, (**C**) antiviral, (**D**) anticancer, (**E**) anti-Gram positive, (**F**) anti-Gram negative, (**G**) anti-mammalian cells, (**H**) anti-HIV, (**I**) anti-MRSA, (**J**) antiparasitic, (**K**) antibiofilm, (**L**) chemotactic, (**M**) endotoxin and (**N**) insecticidal.

We first compared the performance of iAMPCN with that of several state-of-the-art approaches for predicting AMP functional types, including AMAP [35], iAMP-CA2L [42], AMPfun [39], iAMP-RAAC [40] and AMPDiscover [49], based on both balanced and imbalanced independent test datasets, respectively. A statistical summary of the balanced and imbalanced independent test datasets is provided in Supplementary Table S2. Figure 6 shows the ROC curves of the compared methods. Other performance evaluation metrics of these methods on the balanced independent test datasets are provided in Table 6. The corresponding pairwise comparisons (based on the Wilcoxon signed-rank test) of the accuracy, F1, MCC and AUC for measuring the performance differences between these methods are provided in Table 7. The ROC curves and other performance evaluation metrics on the imbalanced independent test datasets are shown in Supplementary Figure S5 and Table S28, respectively. Altogether, we conclude that except for ‘endotoxin activity’, iAMPCN outperforms other compared methods for predicting all the functional activities with the highest accuracy and AUC values. As can be seen from Table 6, several methods including AMAP, AMPDiscover-RF, AMPDiscover-RNN, AMPfun, dbAMP and iAMP-RAAC achieved low precision and specificity values, which indicates that the numbers of their predicted false positive (FP) samples were much higher than those of the predicted true negative (TN) samples since the number of tested negative samples was fixed (Supplementary Table S2). In addition, iAMP-CA2L also achieved low specificity values, which means that the numbers of its predicted false negative (FN) samples were higher than those of the predicted true positive (TP) samples since the number of tested positive samples was fixed (Supplementary Table S2). Higher FN or FP values often result in lower MCC values. When both FN and FP values were very high, the MCC value would tend to be very low, which is the case for iAMP-CA2L for predicting the antibacterial activity (Table 6). Compared with these methods, iAMPCN achieved lower FN or FP values for predicting most functional activities.

**Table 6:** Performances of different computational approaches for predicting AMP functional activities based on balanced independent test datasets

Activity	Method	Precision	Sensitivity	Specificity	Accuracy	F1	MCC	AUC
Antibacterial	AMAP	0.5788	0.8750	0.3633	0.6192	0.6967	0.2774	0.6192
	AMPDiscover-RF	0.6162	0.8803	0.4518	0.6660	0.7250	0.3675	0.6322
	AMPDiscover-RNN	0.6132	0.8727	0.4495	0.6611	0.7203	0.3557	0.6611
	iAMP-CA2L	0.4990	0.6826	0.3148	0.4987	0.5766	-0.0028	0.4987
	iAMPCN	0.6709	0.7914	0.6118	0.7016	0.7262	0.4099	0.7621
Antifungal	AMAP	0.5354	0.8228	0.2860	0.5544	0.6487	0.1289	0.5544
	AMPDiscover-RF	0.5385	0.7851	0.3272	0.5561	0.6388	0.1263	0.5398
	AMPDiscover-RNN	0.5316	0.7895	0.3044	0.5469	0.6354	0.1073	0.5469
	AMPfun	0.5901	0.5746	0.6009	0.5877	0.5822	0.1755	0.6256
	dbAMP	0.5524	0.8044	0.3482	0.5763	0.6550	0.1715	0.6082
	iAMP-CA2L	0.5000	0.2140	0.7860	0.5000	0.2998	0.0000	0.5000
	iAMP-RAAC	0.5850	0.6763	0.5202	0.5982	0.6273	0.1989	0.5982
	iAMPCN	0.7347	0.5368	0.8061	0.6715	0.6204	0.3561	0.7483
Antiviral	AMAP	0.3042	0.2336	0.4656	0.3496	0.2643	-0.3092	0.3496
	AMPDiscover-RF	0.4283	0.5432	0.2749	0.4091	0.4790	-0.1888	0.4999
	AMPDiscover-RNN	0.4447	0.5683	0.2902	0.4293	0.4989	-0.1473	0.4293
	AMPfun	0.5862	0.7502	0.4705	0.6103	0.6582	0.2299	0.6653
	dbAMP	0.5392	0.8618	0.2635	0.5627	0.6633	0.1564	0.6006
	iAMP-CA2L	0.4615	0.0243	0.9717	0.4980	0.0461	-0.0126	0.4980
	iAMP-RAAC	0.6156	0.6653	0.5845	0.6249	0.6395	0.2506	0.6249
	iAMPCN	0.8248	0.7308	0.8448	0.7878	0.7750	0.5794	0.8865
Anticancer	AMAP	0.3973	0.4118	0.3753	0.3935	0.4044	-0.2130	0.3935
	AMPfun	0.4450	0.4172	0.4796	0.4484	0.4306	-0.1034	0.4444
	dbAMP	0.4828	0.6344	0.3204	0.4774	0.5483	-0.0476	0.4759
	iAMP-RAAC	0.4658	0.1172	0.8656	0.4914	0.1873	-0.0259	0.4914
	iAMPCN	0.6830	0.7667	0.6441	0.7054	0.7224	0.4139	0.7698
Anti-Gram positive	AMPfun	0.5607	0.8865	0.3054	0.5960	0.6869	0.2359	0.6304
	dbAMP	0.5513	0.8265	0.3272	0.5768	0.6614	0.1774	0.6107
	iAMP-RAAC	0.5590	0.8879	0.2995	0.5937	0.6860	0.2317	0.5937
	iAMPCN	0.6372	0.7995	0.5449	0.6722	0.7092	0.3561	0.7246
Anti-Gram negative	AMPfun	0.5683	0.8868	0.3262	0.6065	0.6927	0.2572	0.6390
	dbAMP	0.5603	0.8515	0.3317	0.5916	0.6758	0.2144	0.6232
	iAMP-RAAC	0.5635	0.8582	0.3354	0.5968	0.6803	0.2271	0.5968
	iAMPCN	0.6335	0.8058	0.5338	0.6698	0.7093	0.3529	0.7212
Anti-mammalian cells	AMPfun	0.6024	0.3574	0.7641	0.5608	0.4486	0.1330	0.6163
	dbAMP	0.5374	0.6486	0.4416	0.5451	0.5878	0.0923	0.5585
	iAMP-RAAC	0.6364	0.1769	0.8989	0.5379	0.2768	0.1096	0.5379
	iAMPCN	0.6748	0.7942	0.6173	0.7058	0.7297	0.4181	0.7665
Anti-HIV	AMAP	0.3879	0.2830	0.5535	0.4182	0.3273	-0.1699	0.4182
	iAMP-CA2L	0.3333	0.0063	0.9874	0.4969	0.0123	-0.0325	0.4969
	iAMPCN	0.8489	0.7421	0.8679	0.8050	0.7919	0.6149	0.8633
Anti-MRSA	iAMP-CA2L	0.9565	0.4151	0.9811	0.6981	0.5789	0.4806	0.6981
	iAMPCN	0.8600	0.8113	0.8679	0.8396	0.8350	0.6803	0.8597
Antiparasitic	AMAP	0.2692	0.1609	0.5632	0.3621	0.2014	-0.3013	0.3621
	AMPDiscover-RF	0.5000	0.7241	0.2759	0.5000	0.5915	0.0000	0.6092
	AMPDiscover-RNN	0.4841	0.7011	0.2529	0.4770	0.5728	-0.0514	0.4770
	AMPfun	0.4750	0.4368	0.5172	0.4770	0.4551	-0.0461	0.4970
	iAMP-CA2L	0.4000	0.0230	0.9655	0.4943	0.0435	-0.0344	0.4943
	iAMP-RAAC	0.5517	0.1839	0.8506	0.5172	0.2759	0.0463	0.5172
	iAMPCN	0.7534	0.6322	0.7931	0.7126	0.6875	0.4309	0.7861
Antibiofilm	AMAP	0.5965	0.4595	0.6892	0.5743	0.5191	0.1527	0.5743
	iAMP-CA2L	0.8571	0.0811	0.9865	0.5338	0.1481	0.1592	0.5338
	iAMPCN	0.8243	0.8243	0.8243	0.8243	0.8243	0.6486	0.8667
Chemotactic	AMAP	0.4000	0.1429	0.7857	0.4643	0.2105	-0.0933	0.4643
	iAMP-CA2L	0.0000	0.0000	1.0000	0.5000	0.0000	0.0000	0.5000
	iAMPCN	1.0000	0.5000	1.0000	0.7500	0.6667	0.5774	0.8163
Endotoxin	iAMP-CA2L	0.9167	0.6875	0.9375	0.8125	0.7857	0.6455	0.8125
	iAMPCN	0.7059	0.7500	0.6875	0.7188	0.7273	0.4384	0.7617
Insecticidal	AMAP	0.5588	0.2289	0.8193	0.5241	0.3248	0.0597	0.5241
	iAMPCN	0.8514	0.7590	0.8675	0.8133	0.8025	0.6302	0.8936

**Table 7:** The Wilcoxon signed-rank test for measuring the statistical significance of the performance between different approaches in terms of accuracy, F1, MCC and AUC

Accuracy							
Wilcoxon *P*-value	AMAP	AMPDiscover-RF	AMPDiscover-RNN	AMPfun	iAMP-CA2L	iAMP-RAAC	dbAMP
AMPDiscover-RF	–						
AMPDiscover-RNN	–	–					
AMPfun	–	–	–				
iAMP-CA2L	0.008362	–	–	–			
iAMP-RAAC	–	–	–	0.006714	–		
dbAMP	–	–	–	0.030151	–	0.035339	
iAMPCN	6.10E−05	–	–	6.10E−05	0.000122	6.10E−05	6.10E−05
F1							
Wilcoxon *P*-value	AMAP	AMPDiscover-RF	AMPDiscover-RNN	AMPfun	iAMP-CA2L	iAMP-RAAC	dbAMP
AMPDiscover-RF	–						
AMPDiscover-RNN	–	–					
AMPfun	–	–	–				
iAMP-CA2L	0.187622	–	–	–			
iAMP-RAAC	–	–	–	0.106995	–		
dbAMP	–	–	–	0.002014	–	0.00061	
iAMPCN	0.000122	–	–	6.10E−05	0.000122	0.000122	0.000305
MCC							
Wilcoxon *P*-value	AMAP	AMPDiscover-RF	AMPDiscover-RNN	AMPfun	iAMP-CA2L	iAMP-RAAC	dbAMP
AMPDiscover-RF	–						
AMPDiscover-RNN	–	–					
AMPfun	–	–	–				
iAMP-CA2L	0.008362	–	–	–			
iAMP-RAAC	–	–	–	0.008362	–		
dbAMP	–	–	–	0.035339	–	0.047913	
iAMPCN	6.10E−05	6.10E−05	6.10E−05	6.10E−05	0.000183	6.10E−05	6.10E−05
AUC							
Wilcoxon *P*-value	AMAP	AMPDiscover-RF	AMPDiscover-RNN	AMPfun	iAMP-CA2L	iAMP-RAAC	dbAMP
AMPDiscover-RF	–						
AMPDiscover-RNN	–	–					
AMPfun	–	–	–				
iAMP-CA2L	0.008362	–	–	–			
iAMP-RAAC	–	–	–	0.072998	–		
dbAMP	–	–	–	0.035339	–	0.00116	
iAMPCN	6.10E−05	–	–	6.10E−05	0.000122	6.10E−05	6.10E−05

‘–’ means that the number of functional activities that both tools can predict is less than 5.

Nevertheless, it is challenging to improve the performance of iAMPCN in predicting some specific functional activities, including antibacterial, antifungal, anticancer, antiparasitic, endotoxin and anti-mammalian cell activities, as indicated by the low MCC values in Table 6. Previous studies using traditional machine-learning algorithms also demonstrated a similar challenge (as illustrated using the MCC values) in predicting such functional activities based on the constructed datasets of these studies [39, 40]. As such, we would like to argue that predicting these functional activities is difficult, regardless of machine-learning models, feature engineering strategies applied or the training data. Here, we explain the possible reasons: First, the predictions for those functional activities with low MCC values may be related to the killing targets and the mechanism of action. The killing targets of the AMPs with some functional activities are not cells, such as antiviral AMPs, but the targets of the AMPs with antibacterial, antifungal, anticancer, antiparasitic, endotoxin and anti-mammalian cell activities are typically cells, and as such, in order to kill the cells, the cell membrane structure needs to be destroyed. To achieve this, the AMPs tend to possess similar physicochemical properties (e.g. positively charged), structures (e.g. α-helical) and mechanisms of action [101, 102]. Due to their similar physicochemical properties, the AMPs with such functional activities may have high sequence similarities [101, 102]. Our analysis has indeed demonstrated that the amino acid distributions of the positive and negative data of the antibacterial, antifungal, anticancer, antiparasitic, endotoxin and anti-mammalian cell activities are similar (Supplementary Figure 1), thereby making it difficult to distinguish specific functional activities from each other. Second, the dataset imbalance of some activities could also negatively affect the predictive performance. Although we tried several strategies to reduce the impact (e.g. focal loss) and improve the TP and TF values, such improvement was limited. Therefore, some advanced strategies to better handle the imbalanced data need to be developed in the future work. Third, we also utilized the PHOENIX (https://phoenix.arize.com/) tool to explore the embedding performance of representing each peptide sequence. In particular, the embeddings were extracted from the nodes preceding the final dense layer. The training and test datasets of specific functional activities (e.g. antibacterial, antifungal, anticancer and anti-mammalian cells) were utilized as the inputs to the PHOENIX tool. As a result, we observed that some clusters of specific functional activities only contained the points representing the peptides from the test datasets, indicating that the representations of the training data could not provide sufficient information for distinguishing these peptide sequences (Supplementary Figures 6–9). Furthermore, we tried the *t*-distributed stochastic neighbor embedding (*t*-SNE) [103] algorithm to visualize the embedding representations of the test data. For example, although the peptides with antibacterial activities could be roughly clustered into two groups, some peptides still could not be clearly clustered (Supplementary Figure 10). Taken together, the experiments indicate that (i) the sequences between the positive and negative datasets have similarities to a certain extent, (ii) the samples from the training datasets cannot provide enough information for distinguishing the samples of test datasets and (iii) the sequence embedding module (i.e. CNNs and peptide encodings) of iAMPCN needs to be optimized in order to improve the peptide embedding representations in the future work. Lastly, the peptide structure also influences its functional activity. For example, peptides with an α-helical structure are more likely to have antimicrobial activities [101, 102]. In the present study, we only incorporated the physicochemical properties of peptides to encode the sequences to improve the predictive performance, but did not explicitly explore the structural information of peptides. In the future work, we will explore effective strategies to integrate their structural information to further improve the predictive performance of those specific functional activities with low MCC values.

#### Adaptability and stability analysis

To further assess the adaptability in AMP identification, we compared the predictive performance of iAMPCN with that of other existing predictors. We employed the datasets on which these existing predictors were trained and evaluated to evaluate the performance. These predictors include García-Jacas *et al*.’s work [104], BERT-based [105], ACEP [106], APIN-fusion [16], APIN [16], AMPScannerV2 [15], DeepAVP [19], UnidLSTM [19], MultiLSTM [19], DynEvo [19], StaEvo [19], Deep-AmPEP30 [99], Deep-ABPpred [22], AniAMPpred [13], Deep-AFPpred [20], Deep-AVPpred [107] and StaBle-ABPpred [21]. Based on the datasets from Veltri *et al*. [15], Nishant *et al*. [76], Li *et al*. [19], Yan *et al*. [99], Sharma *et al*. [13, 20, 22, 107] and Singh *et al*. [21], the performance evaluation metrics of iAMPCN and these predictors were calculated and provided in Supplementary Tables S29 and S30. It is shown that iAMPCN achieved the highest MCC values on 5 out of 11 test datasets, including the Nishant *et al*. AVP dataset [76], Nishant *et al*. AVP^*^ dataset [76], Sharma *et al*. AMP dataset [13], Sharma *et al*. AFP dataset [20], Singh *et al*. evaluation dataset [21] and Singh *et al*. test dataset [21]. Moreover, iAMPCN also achieved the second-highest MCC values on 2 of 11 test datasets, including Li *et al*. [19] and Sharma *et al*. AVP [107]. These results show that iAMPCN is a very competitive predictor for identifying AMPs and AMPs with one specific functional activity. To further evaluate the performance, iAMPCN was compared with some latest state-of-the-art predictors, including the consensus model [14] and García-Jacas *et al*.’s model [108], based on the Pinacho-Castellanos *et al*.’s general AMP datasets [49] (Supplementary Table S31). The consensus model [14] is an ensemble model combining several predictive results from RNN and BERT [14, 109, 110] models. As a result, iAMPCN achieved an accuracy which was only 0.004 lower than that of the consensus model when being evaluated on the external test dataset, while iAMPCN also achieved a higher accuracy on the test dataset. All these results indicate that iAMPCN has the outstanding adaptability for identifying AMPs or AMPs with one specific functional activity.

As the predictive performance of different models might be affected by the selection of negative data sampling strategies [98], we applied the same datasets and evaluation strategy provided by Sidorczuk *et al.* [98] to further measure the effect caused by the selection of negative data. The corresponding AUC values are listed in Supplementary Table S32. We can see that iAMPCN achieved a much better performance than the other compared methods, with only one average AUC of <0.8. In addition, all the AUC’s standard deviations of iAMPCN were relatively low. In summary, these results suggest that iAMPCN has excellent stability.

### Performance comparison of alternative machine-learning algorithms

To further analyze and evaluate the feature extraction capacity of iAMPCN and the prediction capacity of the final dense layer of iAMPCN, we extracted the feature representation used as inputs into the final dense layer to conduct the comparison. We then replaced the final dense layer with other state-of-the-art machine-learning algorithms, including random forest (RF), adaptive boosting (AdaBoost), XGBoost, logistic regression (LR) and GBDT. In addition, we utilized two oversampling strategies, SMOTE [27] and ADASYN [30], to generate balance datasets. Finally, we compared the AUC values of these algorithms on different functional activity training datasets by 10-fold stratified cross-validation tests and plotted the ROC curves on test datasets. The corresponding performance comparison results are provided in Supplementary Figures S11 and S12, respectively. These results illustrated no significant differences in terms of predictive performance by these machine-learning algorithms. From another perspective, these results also indicate that the feature extraction part by CNN modules of iAMPCN can already learn appropriate feature representations from AMP sequences, leading to outstanding and robust performance, regardless of any machine-learning algorithm in the final dense layer. Furthermore, we conclude that the entire deep-learning framework of iAMPCN outperformed other machine-learning algorithms for the prediction of most functional activities and that the last part of iAMPCN has an excellent predictive capability for the AMP functional activities and accordingly, the trained models have the potential to be applied as effective feature extraction modules in future studies.

Apart from the performance comparison between iAMPCN with several different classic algorithms, we also modified the final dense layers of iAMPCN to evaluate the effect of dense layers on the predictive performance. In particular, we selected several different hidden dense layers, including three hidden layers with the node numbers [128, 64, 32], two hidden layers with the node numbers [128, 64], [64, 32], and one hidden layer with the node number [128], [64] and [32], to compare the performance of iAMPCN which connected all the extracted feature representations to a single final output dense layer without any hidden dense layers by performing 10-fold stratified cross-validation tests. Five datasets were selected for performance evaluation, including the AMP, antibacterial, antifungal, antiviral and antiparasitic datasets. Among these datasets, the antibacterial dataset is nearly balanced for positive and negative samples, while the antiviral and antifungal datasets are imbalanced, and the antiparasitic dataset is very imbalanced. Thus, these selected datasets for the performance comparison are representative. The corresponding AUCs are calculated and listed in Supplementary Table S33. It can be seen that the deep learning structures with more straightforward dense layers achieved higher AUCs and performed better when being tested on the AMP, antibacterial, antifungal and antiviral datasets, while the deep learning structures with different dense layers achieved similar AUC values when being tested on the antiparasitic dataset. Considering that training more complex structures is more time consuming and computationally expensive, the deep learning structure of iAMPCN without the hidden dense layers is more suitable for AMP identification.

### Performance comparison with the unsupervised pretrained model

In addition, we also compared the predictive performance of our proposed iAMPCN method with an unsupervised pretrained model. Particularly, we applied the Evolutionary Scale Modeling (ESM-2) [111] model to extract the sequence features and utilized the RF and XGBoost models to predict AMPs and their functional activities. To make a fair comparison, the same training datasets of iAMPCN were utilized. The predictive performance is shown in Supplementary Tables S34 and S35. As a result, we can see that compared with the other two models, iAMPCN achieved the best MCC value for AMP identification (Supplementary Table S34) and also achieved the best MCC values in predicting 12 out of 14 functional activities (Supplementary Table S35). In addition, we also performed the pairwise comparisons (based on the Wilcoxon signed-rank test), and the results showed that the accuracies and MCCs between our method and the other two methods were significantly different (Supplementary Tables S36), with the only exception for the AUC score between iAMPCN and XGBoost, which was statistically insignificant with the *P*-value of 0.3028.

### Model interpretability analysis

**Figure 7:**
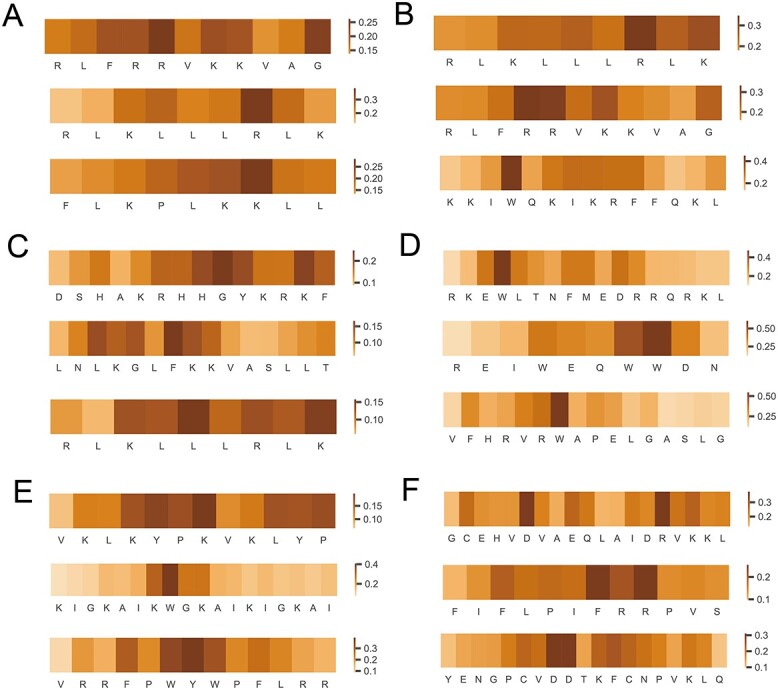
Model interpretability analysis using SHAP values for selected AMPs. (**A**) Anti-Gram negative, (**B**) anti-Gram positive, (**C**) antifungal, (**D**) antiviral, (**E**) anti-mammalian cells and (**F**) anticancer. The value on the color scale of each panel indicates the relative importance. The higher the value, the more important the corresponding amino acid residue for the functional activity of AMPs.

Model interpretability is critically important for researchers to better understand machine learning-based models and make informed decisions regarding the discovery of new potential AMPs. Here, we analyzed the AMPs with the six most common functional activities (i.e. anti-Gram positive, anti-Gram negative, antifungal, antiviral, anti-mammalian cells and anticancer) and applied the ‘GradientExplainer’ method from the SHapley Additive exPlanations (SHAP) algorithm to conduct the interpretability analysis. In particular, we selected three AMPs for each functional activity, and displayed the amino acids’ relative importance for each AMP sequence in Figure 7. We can see that lysine and arginine residues play a relatively important role in antimicrobial activities, presumably due to their positively charged characteristics (Figure 7). This observation is consistent with many previous studies [112, 113]. In addition, leucine, tryptophan and phenylalanine also contribute to antimicrobial activities because of their hydrophobicity (Figure 7) [114, 115]. Furthermore, the aspartic acid was also identified to be significant for anticancer activity (Figure 7), which is also suggested in previous studies [116, 117].

## CONCLUSIONS

Accurate identification of AMPs and their functional activities is critical for functional peptide design and antimicrobial therapy development. In this study, based on a systematic community-wide assessment of computational methods for AMP and their function prediction, we constructed a comprehensive AMP benchmark dataset by integrating a number of public databases and developed a novel deep learning-based framework, iAMPCN, to accurately identify AMPs and their 22 functional activities. Extensive stratified cross-validation tests based on the training datasets and independent test results demonstrated that iAMPCN achieved superior performance for predicting AMPs and most AMP functional types. In addition, the model structure analysis indicates that iAMPCN can be utilized as a feature extraction tool for AMP prediction. The superior predictive performance of iAMPCN can be attributed to three major factors: (i) the reliable dataset curation of the up-to-date annotations of AMPs and their functional activity to provide the most comprehensive training data; (ii) the highly accurate deep-learning framework of iAMPCN learns from the effective feature representations to build robust predictive power for AMP and functional activity prediction; (iii) the selection of appropriate sequence identity thresholds for AMP and function prediction, respectively. Our analysis indicates that a threshold of 40% is sufficient for identifying AMPs, while it is suggested that a higher threshold should be chosen for predicting AMP functional activities. Taken together, we anticipate iAMPCN will be a practical approach for identifying AMPs and their functional activities. In our future work, we will focus on tackling the label imbalance problem to improve model performance in predicting AMP functional activities.

**Key Points**

- We provided a comprehensive summary of existing tools for predicting antimicrobial peptides (AMPs) and their functional activities.
- We constructed comprehensive benchmark datasets of AMPs and 22 functional activities and accordingly developed a two-stage deep learning-based framework, termed iAMPCN, for the identification of AMPs and their functional activities based on four different types of sequence encodings.
- Performance benchmarking based on the independent test datasets suggested that iAMPCN outperformed other available state-of-the-art tools for the prediction of AMPs and the majority of functional activities.
- The source codes of iAMPCN are publicly available at https://github.com/joy50706/iAMPCN/tree/master for the wider research community to use.

## Supplementary Material

Supplementary_Material_updated_2905023_bbad240

## Data Availability

The source codes and data are available at https://github.com/joy50706/iAMPCN/tree/master.

## Author Biographies

**Jing Xu** is currently a PhD student in the Biomedicine Discovery Institute and Department of Biochemistry and Molecular Biology, Monash University, Australia. Her research interests are bioinformatics, computational biology, machine learning and deep learning.

**Fuyi Li** received his PhD in Bioinformatics from Monash University, Australia. He is currently a Professor at the College of Information Engineering, Northwest A&F University, China. His research interests are bioinformatics, computational biology, machine learning and deep learning.

**Chen Li** is a research fellow in the Biomedicine Discovery Institute and Department of Biochemistry and Molecular Biology, Monash University. His research interests include systems proteomics, immunopeptidomics, personalized medicine and experimental bioinformatics.

**Xudong Guo** received his MEng degree from Ningxia University, China. He is currently a research assistant at the College of Information Engineering, Northwest A&F University. His research interests are bioinformatics and data mining.

**Cornelia Landersdorfer** is an associate professor at the Monash Institute of Pharmaceutical Sciences, Monash University, Australia. Her research interests include antibiotic mono- and combination therapy to achieve synergistic bacterial killing and prevent resistance, mechanism-based mathematical modeling, population pharmacokinetics, pharmacodynamics, and optimized dosage regimens of anti-infectives and other agents.

**Hsin-Hui Shen** is an NHMRC Career Development Fellow at Monash University. She completed her PhD in Physical Chemistry at Oxford University in 2008. Her research interests include interaction of polymyxins with bacterial membranes and synergistic effects of antibiotics against Gram-positive bacteria.

**Anton Y. Peleg** is a professor of Infectious Diseases and Microbiology and Director of the Department of Infectious Diseases at the Alfred Hospital and Monash University. He is also a research group leader in the Department of Microbiology, Monash University. His research spans clinical to basic research, with a focus on hospital-acquired infections, antimicrobial resistance, infections in immunocompromised hosts and understanding mechanisms of disease caused by hospital pathogens.

**Jian Li** is a professor and an NHMRC Principal Research Fellow in the Biomedicine Discovery Institute and Department of Microbiology, Monash University, Australia. His research interests include the pharmacology of polymyxins and the discovery of novel, safer polymyxins.

**Seiya Imoto** is professor and director of the Human Genome Center, Institute of Medical Science, The University of Tokyo, Japan. His research interests include genome sequence data analysis, metagenome data analysis, immunogenomics, big data analysis, disease risk prediction and supercomputing in bioinformatics and computational biology.

**Jianhua Yao** is an expert fellow and head of the Medical Algorithm Group in Tencent AI Lab, China. He received his PhD degree from John Hopkins University. His research interests include bioinformatics, computational biology, medical imaging and pattern recognition.

**Tatsuya Akutsu** received his DEng degree in Information Engineering in 1989 from the University of Tokyo, Japan. Since 2001, he has been a professor in the Bioinformatics Centre, Institute for Chemical Research, Kyoto University, Japan. His research interests include bioinformatics and discrete algorithms.

**Jiangning Song** is currently an associate professor and group leader in the Monash Biomedicine Discovery Institute, Monash University. He is also a member of the Monash Data Futures Institute. His research interests include bioinformatics, computational biology, machine learning, medical imaging and pattern recognition.
