# Supplementary material for: iAMPCN: a deep-learning approach for identifying antimicrobial peptides and their functional activities
Source: Brief Bioinform. 2023 Jun 27;24(4):bbad240. doi: 10.1093/bib/bbad240 (PMC10359087; doi:10.1093/bib/bbad240)
Supplement: Supplementary_Material_updated_2905023_bbad240 [file supplementary_material_updated_2905023_bbad240.docx]

**Supplementary Material**

Contents

[Supplementary Methods 4](#_Toc136330333)

[One-hot encoding 4](#_Toc136330334)

[BLOSUM62 encoding 4](#_Toc136330335)

[AAIndex encoding 4](#_Toc136330336)

[PAAC encoding 4](#_Toc136330337)

[Model parameters 4](#_Toc136330338)

[Supplementary References 10](#_Toc136330339)

[Supplementary Tables 11](#_Toc136330340)

[Supplementary Table S1. 11](#_Toc136330341)

[Supplementary Table S2. 12](#_Toc136330342)

[Supplementary Table S3. 14](#_Toc136330343)

[Supplementary Table S4. 16](#_Toc136330344)

[Supplementary Table S5. 17](#_Toc136330345)

[Supplementary Table S6. 18](#_Toc136330346)

[Supplementary Table S7. 19](#_Toc136330347)

[Supplementary Table S8. 20](#_Toc136330348)

[Supplementary Table S9. 21](#_Toc136330349)

[Supplementary Table S10. 22](#_Toc136330350)

[Supplementary Table S11. 23](#_Toc136330351)

[Supplementary Table S12. 24](#_Toc136330352)

[Supplementary Table S13. 25](#_Toc136330353)

[Supplementary Table S14. 26](#_Toc136330354)

[Supplementary Table S15. 27](#_Toc136330355)

[Supplementary Table S16. 28](#_Toc136330356)

[Supplementary Table S17. 29](#_Toc136330357)

[Supplementary Table S18. 30](#_Toc136330358)

[Supplementary Table S19. 31](#_Toc136330359)

[Supplementary Table S20. 32](#_Toc136330360)

[Supplementary Table S21. 33](#_Toc136330361)

[Supplementary Table S22. 34](#_Toc136330362)

[Supplementary Table S23. 35](#_Toc136330363)

[Supplementary Table S24. 36](#_Toc136330364)

[Supplementary Table S25. 37](#_Toc136330365)

[Supplementary Table S26. 38](#_Toc136330366)

[Supplementary Table S27. 39](#_Toc136330367)

[Supplementary Table S28. 40](#_Toc136330368)

[Supplementary Table S29. 42](#_Toc136330369)

[Supplementary Table S30. 44](#_Toc136330370)

[Supplementary Table S31. 45](#_Toc136330371)

[Supplementary Table S32. 46](#_Toc136330372)

[Supplementary Table S33. 51](#_Toc136330373)

[Supplementary Table S34. 52](#_Toc136330374)

[Supplementary Table S35. 53](#_Toc136330375)

[Supplementary Table S36 54](#_Toc136330376)

[Supplementary Figure 55](#_Toc136330377)

[Supplementary Figure S1. 55](#_Toc136330378)

[Supplementary Figure S2. 57](#_Toc136330379)

[Supplementary Figure S3. 58](#_Toc136330380)

[Supplementary Figure S4. 60](#_Toc136330381)

[Supplementary Figure S5. 61](#_Toc136330382)

[Supplementary Figure S6. 62](#_Toc136330383)

[Supplementary Figure S7. 63](#_Toc136330384)

[Supplementary Figure S8. 64](#_Toc136330385)

[Supplementary Figure S9. 65](#_Toc136330386)

[Supplementary Figure S10. 66](#_Toc136330387)

[Supplementary Figure S11. 67](#_Toc136330388)

[Supplementary Figure S12. 69](#_Toc136330389)

# Supplementary Methods

## One-hot encoding

A peptide sequence is converted into a numerical matrix with the dimension of 200*21, where “200” represents the length of a peptide sequence, and a 21-dimensional binary vector represents each amino acid type. The amino acids are first sorted in a certain order and the corresponding position of the binary vector represent an amino acid. For example, the one-hot encoding of A is represented as [1,0,0,0,0,0,0,0,0,0,0,0,0,0,0,0,0,0,0,0,0], C is [0,1,0,0,0,0,0,0,0,0,0,0,0,0,0,0,0,0,0,0,0], and the one-hot encoding of those non-standard amino acids is [0,0,0,0,0,0,0,0,0,0,0,0,0,0,0,0,0,0,0,0,1].

## BLOSUM62 encoding

The BLOSUM62 [1] matrix indicates the relative frequency and probability of amino acid substitution, which represents primary sequence information.

## AAIndex encoding

AAindex [2, 3] is a database containing various physicochemical and biochemical properties of amino acids and pairs of amino acids. Here, we utilized 531 properties of amino acids that were extracted by iFeature [4].

## PAAC encoding

This group of descriptors is the original hydrophobicity values, the original hydrophilicity values, and the original side chain masses of the 20 natural amino acids, respectively, which were utilized to compute pseudo-amino acid composition (PAAC) [5, 6]. Therefore, we named this group of descriptors PAAC.

## Model parameters

==========================================================================================

Layer (type:depth-idx) Output Shape Param #

==========================================================================================

iAMPCN -- --

├─ModuleList: 1-1 -- --

├─ModuleList: 1-2 -- --

├─ModuleList: 1-3 -- --

├─ModuleList: 1-4 -- --

├─ModuleList: 1-1 -- --

│ └─Sequential: 2-1 [128, 64, 1] --

│ │ └─Conv1d: 3-1 [128, 64, 499] 68,032

│ │ └─BatchNorm1d: 3-2 [128, 64, 499] 128

│ │ └─ReLU: 3-3 [128, 64, 499] --

│ │ └─MaxPool1d: 3-4 [128, 64, 1] --

│ └─Sequential: 2-2 [128, 64, 1] --

│ │ └─Conv1d: 3-5 [128, 64, 498] 102,016

│ │ └─BatchNorm1d: 3-6 [128, 64, 498] 128

│ │ └─ReLU: 3-7 [128, 64, 498] --

│ │ └─MaxPool1d: 3-8 [128, 64, 1] --

│ └─Sequential: 2-3 [128, 64, 1] --

│ │ └─Conv1d: 3-9 [128, 64, 497] 136,000

│ │ └─BatchNorm1d: 3-10 [128, 64, 497] 128

│ │ └─ReLU: 3-11 [128, 64, 497] --

│ │ └─MaxPool1d: 3-12 [128, 64, 1] --

│ └─Sequential: 2-4 [128, 64, 1] --

│ │ └─Conv1d: 3-13 [128, 64, 496] 169,984

│ │ └─BatchNorm1d: 3-14 [128, 64, 496] 128

│ │ └─ReLU: 3-15 [128, 64, 496] --

│ │ └─MaxPool1d: 3-16 [128, 64, 1] --

│ └─Sequential: 2-5 [128, 64, 1] --

│ │ └─Conv1d: 3-17 [128, 64, 495] 203,968

│ │ └─BatchNorm1d: 3-18 [128, 64, 495] 128

│ │ └─ReLU: 3-19 [128, 64, 495] --

│ │ └─MaxPool1d: 3-20 [128, 64, 1] --

├─ModuleList: 1-2 -- --

│ └─Sequential: 2-6 [128, 64, 1] --

│ │ └─Conv1d: 3-21 [128, 64, 499] 2,752

│ │ └─BatchNorm1d: 3-22 [128, 64, 499] 128

│ │ └─ReLU: 3-23 [128, 64, 499] --

│ │ └─MaxPool1d: 3-24 [128, 64, 1] --

│ └─Sequential: 2-7 [128, 64, 1] --

│ │ └─Conv1d: 3-25 [128, 64, 498] 4,096

│ │ └─BatchNorm1d: 3-26 [128, 64, 498] 128

│ │ └─ReLU: 3-27 [128, 64, 498] --

│ │ └─MaxPool1d: 3-28 [128, 64, 1] --

│ └─Sequential: 2-8 [128, 64, 1] --

│ │ └─Conv1d: 3-29 [128, 64, 497] 5,440

│ │ └─BatchNorm1d: 3-30 [128, 64, 497] 128

│ │ └─ReLU: 3-31 [128, 64, 497] --

│ │ └─MaxPool1d: 3-32 [128, 64, 1] --

│ └─Sequential: 2-9 [128, 64, 1] --

│ │ └─Conv1d: 3-33 [128, 64, 496] 6,784

│ │ └─BatchNorm1d: 3-34 [128, 64, 496] 128

│ │ └─ReLU: 3-35 [128, 64, 496] --

│ │ └─MaxPool1d: 3-36 [128, 64, 1] --

│ └─Sequential: 2-10 [128, 64, 1] --

│ │ └─Conv1d: 3-37 [128, 64, 495] 8,128

│ │ └─BatchNorm1d: 3-38 [128, 64, 495] 128

│ │ └─ReLU: 3-39 [128, 64, 495] --

│ │ └─MaxPool1d: 3-40 [128, 64, 1] --

├─ModuleList: 1-3 -- --

│ └─Sequential: 2-11 [128, 64, 1] --

│ │ └─Conv1d: 3-41 [128, 64, 499] 3,008

│ │ └─BatchNorm1d: 3-42 [128, 64, 499] 128

│ │ └─ReLU: 3-43 [128, 64, 499] --

│ │ └─MaxPool1d: 3-44 [128, 64, 1] --

│ └─Sequential: 2-12 [128, 64, 1] --

│ │ └─Conv1d: 3-45 [128, 64, 498] 4,480

│ │ └─BatchNorm1d: 3-46 [128, 64, 498] 128

│ │ └─ReLU: 3-47 [128, 64, 498] --

│ │ └─MaxPool1d: 3-48 [128, 64, 1] --

│ └─Sequential: 2-13 [128, 64, 1] --

│ │ └─Conv1d: 3-49 [128, 64, 497] 5,952

│ │ └─BatchNorm1d: 3-50 [128, 64, 497] 128

│ │ └─ReLU: 3-51 [128, 64, 497] --

│ │ └─MaxPool1d: 3-52 [128, 64, 1] --

│ └─Sequential: 2-14 [128, 64, 1] --

│ │ └─Conv1d: 3-53 [128, 64, 496] 7,424

│ │ └─BatchNorm1d: 3-54 [128, 64, 496] 128

│ │ └─ReLU: 3-55 [128, 64, 496] --

│ │ └─MaxPool1d: 3-56 [128, 64, 1] --

│ └─Sequential: 2-15 [128, 64, 1] --

│ │ └─Conv1d: 3-57 [128, 64, 495] 8,896

│ │ └─BatchNorm1d: 3-58 [128, 64, 495] 128

│ │ └─ReLU: 3-59 [128, 64, 495] --

│ │ └─MaxPool1d: 3-60 [128, 64, 1] --

├─BatchNorm1d: 1-5 [128, 3, 500] 6

├─ModuleList: 1-4 -- --

│ └─Sequential: 2-16 [128, 64, 1] --

│ │ └─Conv1d: 3-61 [128, 64, 499] 448

│ │ └─BatchNorm1d: 3-62 [128, 64, 499] 128

│ │ └─ReLU: 3-63 [128, 64, 499] --

│ │ └─MaxPool1d: 3-64 [128, 64, 1] --

│ └─Sequential: 2-17 [128, 64, 1] --

│ │ └─Conv1d: 3-65 [128, 64, 498] 640

│ │ └─BatchNorm1d: 3-66 [128, 64, 498] 128

│ │ └─ReLU: 3-67 [128, 64, 498] --

│ │ └─MaxPool1d: 3-68 [128, 64, 1] --

│ └─Sequential: 2-18 [128, 64, 1] --

│ │ └─Conv1d: 3-69 [128, 64, 497] 832

│ │ └─BatchNorm1d: 3-70 [128, 64, 497] 128

│ │ └─ReLU: 3-71 [128, 64, 497] --

│ │ └─MaxPool1d: 3-72 [128, 64, 1] --

│ └─Sequential: 2-19 [128, 64, 1] --

│ │ └─Conv1d: 3-73 [128, 64, 496] 1,024

│ │ └─BatchNorm1d: 3-74 [128, 64, 496] 128

│ │ └─ReLU: 3-75 [128, 64, 496] --

│ │ └─MaxPool1d: 3-76 [128, 64, 1] --

│ └─Sequential: 2-20 [128, 64, 1] --

│ │ └─Conv1d: 3-77 [128, 64, 495] 1,216

│ │ └─BatchNorm1d: 3-78 [128, 64, 495] 128

│ │ └─ReLU: 3-79 [128, 64, 495] --

│ │ └─MaxPool1d: 3-80 [128, 64, 1] --

├─MaxPool1d: 1-6 [128, 64, 1] --

├─MaxPool1d: 1-7 [128, 64, 1] --

├─MaxPool1d: 1-8 [128, 64, 1] --

├─MaxPool1d: 1-9 [128, 64, 1] --

├─Linear: 1-10 [128, 1] 257

├─Sigmoid: 1-11 [128, 1] --

==========================================================================================

The model with one-hot vector encoding with 21 bits and the model with one-hot vector encoding with 20 bits are the same when training and testing the sequences with usual amino acids. A detailed description is provided as follows:

Let $X_{n*21}$ be the one-hot vector encoding with 21 bits, $W_{m*21}^{k}$ is the *k*th Conv1d kernel:

$$X_{n*21}=\left[ \begin{matrix} x_{1,1} & \cdots& x_{1,21} \\ \vdots& \ddots& \vdots\\ x_{n,1} & \cdots& x_{n,21} \end{matrix} \right]$$

For sequences with usual amino acids, $x_{i,21}=0$, so $x_{i,21}w_{t, 21}^{k}=0$. After the Conv1d computation, the $w_{t, 21}^{k}$ disappears and does not contribute to the final loss function. Therefore, the structure and the loss function of the model with one-hot vector encoding with 21 bits are the same as those of the model with one-hot vector encoding with 20 bits. As a result, the optimized parameters (except $w_{t, 21}^{k}$) of the model with one-hot vector encoding with 21 bits are the same as those of the model with one-hot vector encoding with 20 bits. When testing the sequences with usual amino acids, because $x_{i,21}w_{t, 21}^{k}=0$, $w_{t, 21}^{k}$ would not affect the prediction output, and as such, the prediction results of the model with one-hot vector encoding with 21 bits are the same as those of the model with one-hot vector encoding with 20 bits. Therefore, it is unnecessary to modify the original model as that with one-hot vector encoding with 20 bits and retrain all the new models if we just apply the models to the sequences with usual amino acids. Accordingly, we have modified the source codes to be able to provide the warning message to the user so that the user can be aware of the sequences with unusual amino acids as the input.

# Supplementary References

1. Eddy SR. Where did the BLOSUM62 alignment score matrix come from?, Nature Biotechnology 2004;22:1035-1036.

2. Kawashima S, Ogata H, Kanehisa M. AAindex: Amino Acid Index Database, Nucleic Acids Research 1999;27:368-369.

3. Kawashima S, Pokarowski P, Pokarowska M et al. AAindex: amino acid index database, progress report 2008, Nucleic Acids Research 2007;36:D202-D205.

4. Chen Z, Zhao P, Li F et al. iFeature: a Python package and web server for features extraction and selection from protein and peptide sequences, Bioinformatics 2018;34:2499-2502.

5. Chou K-C. Prediction of protein cellular attributes using pseudo-amino acid composition, Proteins: Structure, Function, and Bioinformatics 2001;43:246-255.

6. Chou K-C. Using amphiphilic pseudo amino acid composition to predict enzyme subfamily classes, Bioinformatics 2004;21:10-19.

# Supplementary Tables

Supplementary Table S1. A summary of the datasets for AMP prediction.

| **Pos/Neg** | **Training dataset** | **Training dataset**  **with sequence redudandancy removal using CD-HIT** | | | | | | | **Test data set** | **Independent test dataset** |
| --- | --- | --- | --- | --- | --- | --- | --- | --- | --- | --- |
|  |  | 40% | 50% | 60% | 70% | 80% | 90% | 100% |  |  |
| Positive | 40799 | 2300 | 7418 | 13010 | 15293 | 17109 | 20795 | 27928 | 8316 | 4211 |
| Negative | 156420 | 33651 | 47088 | 62419 | 78652 | 95805 | 115797 | 156056 | 39105 | 10691 |

Supplementary Table S2. **A summary of the datasets for AMP’s functional activities prediction.**

| **Activity** | **Pos/Neg** | **Training dataset** | **Training datasets after CD-HIT** | | | | | | | **Test data set** | **Balanced independent test dataset** | **Imbalanced independent test dataset** |
| --- | --- | --- | --- | --- | --- | --- | --- | --- | --- | --- | --- | --- |
|  |  |  | 40% | 50% | 60% | 70% | 80% | 90% | 100% |  |  |  |
| antibacterial | positive | 12868 | 997 | 1731 | 2771 | 3646 | 4540 | 6223 | 8865 | 3190 | 2656 | 3177 |
|  | negative | 10843 | 1508 | 3047 | 4596 | 5259 | 5749 | 6607 | 7865 | 2708 | 2656 | 2656 |
| antibiofilm | positive | 298 | 63 | 118 | 197 | 211 | 223 | 233 | 242 | 74 | 74 | 74 |
|  | negative | 23361 | 2036 | 4182 | 6741 | 8294 | 9675 | 12141 | 16218 | 5841 | 74 | 5763 |
| anticancer | positive | 3759 | 964 | 1644 | 2173 | 2451 | 2693 | 2964 | 3262 | 939 | 930 | 930 |
|  | negative | 19901 | 1675 | 3184 | 5268 | 6645 | 7847 | 10083 | 13630 | 4975 | 930 | 4741 |
| anticandidal | positive | 536 | 139 | 224 | 283 | 337 | 382 | 434 | 520 | 133 | / | / |
|  | negative | 23124 | 2050 | 4172 | 6792 | 8363 | 9696 | 12125 | 16024 | 5781 | / | / |
| antifungal | positive | 4764 | 668 | 1182 | 1721 | 2145 | 2566 | 3091 | 3926 | 1191 | 1140 | 1140 |
|  | negative | 18895 | 1782 | 3656 | 5855 | 7018 | 8009 | 9986 | 12993 | 4724 | 1140 | 4551 |
| anti-Gram negative | positive | 6840 | 600 | 1190 | 1901 | 2480 | 3105 | 3953 | 5346 | 1697 | 1643 | 1643 |
|  | negative | 16830 | 1884 | 3751 | 5864 | 6910 | 7766 | 9451 | 11809 | 4214 | 1643 | 4097 |
| anti-Gram positive | positive | 6473 | 600 | 1188 | 1896 | 2456 | 3060 | 3834 | 5101 | 1580 | 1516 | 1516 |
|  | negative | 17209 | 1888 | 3747 | 5871 | 6946 | 7790 | 9576 | 12092 | 4330 | 1516 | 4207 |
| anti-HIV | positive | 650 | 134 | 205 | 247 | 276 | 318 | 398 | 509 | 162 | 159 | 159 |
|  | negative | 23009 | 2051 | 4166 | 6796 | 8378 | 9718 | 12112 | 16097 | 5753 | 159 | 5673 |
| antimalarial | positive | 59 | 35 | 42 | 47 | 50 | 51 | 53 | 57 | 14 | / | / |
|  | negative | 23601 | 2033 | 4205 | 6876 | 8480 | 9876 | 12365 | 16459 | 5900 | / | / |
| anti-MRSA | positive | 214 | 75 | 115 | 152 | 161 | 174 | 187 | 202 | 53 | 53 | 53 |
|  | negative | 23445 | 2033 | 4176 | 6834 | 8420 | 9806 | 12298 | 16359 | 5862 | 53 | 6150 |
| antiparasitic | positive | 366 | 99 | 147 | 183 | 214 | 236 | 264 | 313 | 91 | 87 | 87 |
|  | negative | 23293 | 2015 | 4169 | 6825 | 8403 | 9771 | 12247 | 16287 | 5824 | 87 | 5738 |
| antiplasmodial | positive | 51 | 19 | 21 | 23 | 28 | 32 | 33 | 37 | 12 | / | / |
|  | negative | 23609 | 2039 | 4195 | 6891 | 8487 | 9862 | 12345 | 16449 | 5902 | / | / |
| antiprotozoal | positive | 43 | 20 | 25 | 32 | 33 | 38 | 39 | 43 | 10 | / | / |
|  | negative | 23617 | 2048 | 4206 | 6889 | 8488 | 9871 | 12358 | 16442 | 5904 | / | / |
| anti-TB | positive | 220 | 50 | 63 | 71 | 75 | 83 | 96 | 107 | 55 | / | / |
|  | negative | 23439 | 2037 | 4200 | 6844 | 8437 | 9808 | 12293 | 16397 | 5860 | / | / |
| antiviral | positive | 5209 | 635 | 1219 | 1686 | 1997 | 2262 | 2778 | 3525 | 1286 | 1237 | 1237 |
|  | negative | 18464 | 1830 | 3551 | 5684 | 6972 | 8110 | 10112 | 13384 | 4629 | 1237 | 4439 |
| anti-mammalian cells | positive | 3476 | 319 | 645 | 1038 | 1326 | 1661 | 2136 | 2812 | 869 | 831 | 831 |
|  | negative | 20183 | 2007 | 4036 | 6509 | 7839 | 8930 | 10985 | 14062 | 5046 | 831 | 4915 |
| anuran defense | positive | 624 | 64 | 109 | 191 | 273 | 358 | 455 | 612 | 155 | / | / |
|  | negative | 23036 | 2039 | 4192 | 6857 | 8397 | 9709 | 12097 | 15977 | 5759 | / | / |
| chemotactic | positive | 68 | 44 | 56 | 59 | 62 | 63 | 65 | 66 | 17 | 14 | 14 |
|  | negative | 23591 | 2022 | 4179 | 6828 | 8433 | 9820 | 12313 | 16399 | 5898 | 14 | 5816 |
| cytotoxic | positive | 144 | 36 | 55 | 73 | 82 | 89 | 106 | 134 | 36 | / | / |
|  | negative | 23515 | 2045 | 4200 | 6879 | 8469 | 9823 | 12283 | 16355 | 5879 | / | / |
| endotoxin | positive | 66 | 45 | 51 | 57 | 61 | 62 | 65 | 65 | 16 | 16 | 16 |
|  | negative | 23593 | 2037 | 4216 | 6888 | 8489 | 9855 | 12377 | 16481 | 5899 | 16 | 5985 |
| hemolytic | positive | 1907 | 170 | 328 | 511 | 660 | 859 | 1148 | 1643 | 476 | / | / |
|  | negative | 21753 | 2036 | 4164 | 6766 | 8238 | 9434 | 11654 | 15089 | 5438 | / | / |
| insecticidal | positive | 332 | 137 | 179 | 200 | 216 | 224 | 244 | 291 | 83 | 83 | 83 |
|  | negative | 23327 | 2036 | 4169 | 6799 | 8376 | 9716 | 12223 | 16249 | 5832 | 83 | 5752 |

Supplementary Table S3. **Preliminary comparison of the predictive performance based on the AMPfun datasets.**

|  | **Tool** |  |  |  |  | **AUC** |
| --- | --- | --- | --- | --- | --- | --- |
| AMPs | AMPfun |  |  |  |  | 0.9894 |
|  | iAMPCN (one-hot) |  |  |  |  | 0.9854 |
|  | iAMPCN (BLOSUM62) |  |  |  |  | 0.9861 |
|  | iAMPCN (AAINDEX) |  |  |  |  | 0.9854 |
|  | iAMPCN (PAAC) |  |  |  |  | 0.9560 |
|  | iAMPCN (one-hot+AAI+PAAC+BLOSUM62) |  |  |  |  | 0.9905 |
| **Activities** | **Tool** | **Sensitivity** | **Specificity** | **Accuracy** | **MCC** | **AUC** |
| antiviral | AMPfun | 0.9085 | 0.8406 | 0.8613 | 0.7075 | 0.9404 |
|  | iAMPpred | 0.3128 | 0.3959 | 0.3706 | −0.2682 | 0.3158 |
|  | AVPpred | 0.2409 | 0.8857 | 0.6901 | 0.1643 | N.A. |
|  | iAMPCN (one-hot) | 0.8741 | 0.8852 | 0.8775 | 0.7292 | 0.9360 |
|  | iAMPCN (BLOSUM62) | 0.8850 | 0.8486 | 0.8739 | 0.7134 | 0.9285 |
|  | iAMPCN (AAINDEX) | 0.7766 | 0.9334 | 0.8243 | 0.6582 | 0.9303 |
|  | iAMPCN (PAAC) | 0.8850 | 0.6256 | 0.8061 | 0.5290 | 0.8545 |
|  | iAMPCN (one-hot+AAI+PAAC+BLOSUM62) | 0.8981 | 0.8802 | 0.8927 | 0.7567 | 0.9405 |
| anticancer | AMPfun | 0.7766 | 0.7060 | 0.7094 | 0.2208 | 0.8231 |
|  | MLACP | 0.7234 | 0.7512 | 0.7499 | 0.2272 | 0.8320 |
|  | iAMPCN (one-hot) | 0.8889 | 0.5638 | 0.8734 | 0.2842 | 0.7998 |
|  | iAMPCN (BLOSUM62) | 0.8458 | 0.7234 | 0.8400 | 0.3146 | 0.8352 |
|  | iAMPCN (AAINDEX) | 0.8729 | 0.6383 | 0.8618 | 0.3037 | 0.8173 |
|  | iAMPCN (PAAC) | 0.7943 | 0.5638 | 0.7833 | 0.1832 | 0.7395 |
|  | iAMPCN (one-hot+AAI+PAAC+BLOSUM62) | 0.8549 | 0.7128 | 0.8481 | 0.3201 | 0.8390 |
| antifungal | AMPfun | 0.7929 | 0.7455 | 0.7678 | 0.5375 | 0.8448 |
|  | AntiFP | 0.6699 | 0.7039 | 0.6879 | 0.3737 | N.A. |
|  | iAMPCN (one-hot) | 0.7255 | 0.8061 | 0.7590 | 0.5240 | 0.8361 |
|  | iAMPCN (BLOSUM62) | 0.6693 | 0.8488 | 0.7438 | 0.5127 | 0.8242 |
|  | iAMPCN (AAINDEX) | 0.6814 | 0.8268 | 0.7418 | 0.5017 | 0.8110 |
|  | iAMPCN (PAAC) | 0.6805 | 0.7012 | 0.6891 | 0.3766 | 0.7471 |
|  | iAMPCN (one-hot+AAI+PAAC+BLOSUM62) | 0.7801 | 0.7683 | 0.7752 | 0.5434 | 0.8502 |
| anti-Gram positive | AMPfun | 0.8829 | 0.6282 | 0.7385 | 0.5155 | 0.8653 |
|  | iAMPpred | 0.6872 | 0.6541 | 0.6684 | 0.3382 | 0.6950 |
|  | iAMPCN (one-hot) | 0.6748 | 0.8684 | 0.7559 | 0.5391 | 0.8610 |
|  | iAMPCN (BLOSUM62) | 0.6094 | 0.9191 | 0.7392 | 0.5352 | 0.8480 |
|  | iAMPCN (AAINDEX) | 0.6661 | 0.8635 | 0.7489 | 0.5260 | 0.8401 |
|  | iAMPCN (PAAC) | 0.5562 | 0.8031 | 0.6597 | 0.3612 | 0.7493 |
|  | iAMPCN (one-hot+AAI+PAAC+BLOSUM62) | 0.7716 | 0.8333 | 0.7975 | 0.5974 | 0.8728 |
| anti-Gram negative | AMPfun | 0.8563 | 0.6522 | 0.7406 | 0.5086 | 0.8590 |
|  | iAMPpred | 0.6932 | 0.6568 | 0.6726 | 0.3469 | 0.6958 |
|  | iAMPCN (one-hot) | 0.7010 | 0.8587 | 0.7671 | 0.5536 | 0.8643 |
|  | iAMPCN (BLOSUM62) | 0.7044 | 0.8708 | 0.7742 | 0.5692 | 0.8664 |
|  | iAMPCN (AAINDEX) | 0.6731 | 0.8696 | 0.7554 | 0.5387 | 0.8562 |
|  | iAMPCN (PAAC) | 0.6617 | 0.7174 | 0.6851 | 0.3741 | 0.7623 |
|  | iAMPCN (one-hot+AAI+PAAC+BLOSUM62) | 0.8099 | 0.7995 | 0.8056 | 0.6050 | 0.8847 |
| Targeting mammals | AMPfun | 0.7849 | 0.8045 | 0.8035 | 0.2998 | 0.8648 |
|  | iAMPCN (one-hot) | 0.8374 | 0.6882 | 0.8304 | 0.2853 | 0.8638 |
|  | iAMPCN (BLOSUM62) | 0.8443 | 0.7204 | 0.8385 | 0.3099 | 0.8835 |
|  | iAMPCN (AAINDEX) | 0.8491 | 0.7527 | 0.8446 | 0.3324 | 0.8831 |
|  | iAMPCN (PAAC) | 0.8183 | 0.6129 | 0.8086 | 0.2275 | 0.7546 |
|  | iAMPCN (one-hot+AAI+PAAC+BLOSUM62) | 0.8533 | 0.7634 | 0.8491 | 0.3433 | 0.8940 |
| antiparasitic | AMPfun | 0.6167 | 0.7732 | 0.7685 | 0.1570 | 0.7773 |
|  | iAMPCN (one-hot) | 0.9775 | 0.0833 | 0.9504 | 0.0678 | 0.6973 |
|  | iAMPCN (BLOSUM62) | 0.9634 | 0.2833 | 0.9428 | 0.2064 | 0.7246 |
|  | iAMPCN (AAINDEX) | 0.9420 | 0.3000 | 0.9225 | 0.1681 | 0.7100 |
|  | iAMPCN (PAAC) | 0.5110 | 0.7667 | 0.5187 | 0.0953 | 0.6567 |
|  | iAMPCN (one-hot+AAI+PAAC+BLOSUM62) | 0.7936 | 0.6167 | 0.7882 | 0.1704 | 0.7805 |

Supplementary Table S4. **Preliminary comparison of predictive performance based on the iAMP-2L datasets.**

| **Method** | **Absolute true↑** | **Aiming↑** | **Recall↑** | **Accuracy↑** | **Absolute false↓** |
| --- | --- | --- | --- | --- | --- |
| iAMP-2L | 0.4305 | 0.8331 | 0.7570 | 0.6687 | 0.1640 |
| MLAMP | 0.4846 | 0.8338 | 0.7631 | 0.6864 | 0.1595 |
| iAMP-CA2L | 0.5943 | 0.9169 | 0.8112 | 0.7737 | **0.0582** |
| iAMPCN | **0.6420** | **0.9198** | **0.8173** | **0.7909** | 0.1009 |

Supplementary Table S5. **Preliminary comparison of predictive performance based on the iAMP-CA2L and iAMP-2L datasets.**

| **Testing on an AMP independent test dataset from iAMP-2L** | | | | |
| --- | --- | --- | --- | --- |
| **AMPs** | **iAMP-2L** | **MLAMP** | **iAMP-CA2L** | **iAMPCN** |
| ***Acc*** | 0.9223 | 0.9470 | 0.9612 | **0.9674** |
| **MCC** | 0.8446 | 0.8950 | 0.9187 | **0.9348** |
| ***Sn*** | 0.9772 | 0.9730 | **0.9747** | 0.9717 |
| ***Sp*** | 0.8674 | 0.9210 | 0.9405 | **0.9630** |
| **Precision** | - | - | 0.9618 | **0.9634** |
| **F1** | - | - | **0.9682** | 0.9675 |
| **Testing on AMP activities benchmarking datasets from iAMP-CA2L** | | | | |
| **Activities** | **iAMP-2L** | **MLAMP** | **iAMP-CA2L** | **iAMPCN** |
| **Subset accuracy↑** | 0.4631 | 0.4982 | 0.5585 | **0.5841** |
| **Average precision↑** | 0.7379 | 0.7606 | 0.8057 | **0.9142** |
| **Coverage↓** | 4.2949 | 3.3658 | 2.8977 | **2.4354** |
| **Hamming loss↓** | 0.1026 | 0.1018 | 0.0831 | **0.0631** |
| **Ranking loss↓** | 0.3476 | 0.2579 | 0.2413 | **0.0553** |
| **One error↓** | 0.0973 | 0.0897 | 0.0672 | **0.0240** |
| **MacroAveragedPrecision↑** | 0.1856 | 0.1988 | **0.2889** | 0.2445 |
| **MacroAveragedSensitivity↑** | 0.1111 | 0.1696 | 0.2587 | **0.9815** |
| **MacroAveragedF_score↑** | 0.1051 | 0.1576 | 0.3099 | **0.3316** |
| **MicroAveragedPrecision↑** | 0.5682 | 0.6049 | **0.6724** | 0.6639 |
| **MicroAveragedSensitivity↑** | 0.5375 | 0.6563 | 0.6735 | **0.9478** |
| **MicroAveragedF_score↑** | 0.7623 | 0.7309 | 0.7698 | **0.7808** |

Supplementary Table S6. **Performances of 10-fold stratified cross-validation test based on the antibacterial training dataset.**

| **CD-HIT** | **Sensitivity** | **Specificity** | **Accuracy** | **MCC** | **AUC** |
| --- | --- | --- | --- | --- | --- |
| 40% | 0.7187($\pm$0.0695) | 0.7142($\pm$0.0616) | 0.7165($\pm$0.0360) | 0.4278($\pm$0.0708) | 0.7737($\pm$0.0316) |
| 50% | 0.7195($\pm$0.0670) | 0.7123($\pm$0.0504) | 0.7159($\pm$0.0145) | 0.4202($\pm$0.0267) | 0.7724($\pm$0.0202) |
| 60% | 0.7190($\pm$0.0489) | 0.7125($\pm$0.0444) | 0.7157($\pm$0.0187) | 0.4216($\pm$0.0373) | 0.7712($\pm$0.0183) |
| 70% | 0.7577($\pm$0.0337) | 0.7083($\pm$0.0309) | 0.7330($\pm$0.0137) | 0.4591($\pm$0.0263) | 0.7996($\pm$0.0140) |
| 80% | 0.7613($\pm$0.0416) | 0.7434($\pm$0.0416) | 0.7523($\pm$0.0122) | 0.5031($\pm$0.0233) | 0.8204($\pm$0.0126) |
| 90% | 0.8041($\pm$0.0139) | 0.7700($\pm$0.0187) | 0.7871($\pm$0.0097) | 0.5741($\pm$0.0194) | 0.8614($\pm$0.0129) |
| 100% | 0.8268($\pm$0.0193) | 0.8301($\pm$0.0258) | 0.8284($\pm$0.0078) | 0.6567($\pm$0.0151) | 0.9029($\pm$0.0069) |

Supplementary Table S7. **Performances of 10-fold stratified cross-validation test based on the anti-Gram-positive training dataset.**

| **CD-HIT** | **Sensitivity** | **Specificity** | **Accuracy** | **MCC** | **AUC** |
| --- | --- | --- | --- | --- | --- |
| 40% | 0.6815($\pm$0.0817) | 0.6501($\pm$0.0742) | 0.6658($\pm$0.0277) | 0.2891($\pm$0.0485) | 0.7223($\pm$0.0290) |
| 50% | 0.6517($\pm$0.2283) | 0.6890($\pm$0.1261) | 0.6703($\pm$0.0579) | 0.2969($\pm$0.1016) | 0.7484($\pm$0.0124) |
| 60% | 0.7139($\pm$0.0597) | 0.6829($\pm$0.0347) | 0.6984($\pm$0.0185) | 0.3463($\pm$0.0288) | 0.7556($\pm$0.0140) |
| 70% | 0.7325($\pm$0.0395) | 0.6757($\pm$0.0352) | 0.7041($\pm$0.0095) | 0.3625($\pm$0.0169) | 0.7637($\pm$0.0071) |
| 80% | 0.7700($\pm$0.0372) | 0.6613($\pm$0.0227) | 0.7157($\pm$0.0152) | 0.3893($\pm$0.0271) | 0.7756($\pm$0.0158) |
| 90% | 0.7870($\pm$0.0370) | 0.6865($\pm$0.0278) | 0.7368($\pm$0.0129) | 0.4303($\pm$0.0232) | 0.8042($\pm$0.0149) |
| 100% | 0.8053($\pm$0.0286) | 0.7382($\pm$0.0250) | 0.7718($\pm$0.0089) | 0.5032($\pm$0.0170) | 0.8462($\pm$0.0090) |

Supplementary Table S8. **Performances of 10-fold stratified cross-validation test based on the anti-Gram-negative training dataset.**

| **CD-HIT** | **Sensitivity** | **Specificity** | **Accuracy** | **MCC** | **AUC** |
| --- | --- | --- | --- | --- | --- |
| 40% | 0.6500($\pm$0.0997) | 0.6466($\pm$0.0639) | 0.6483($\pm$0.0402) | 0.2588($\pm$0.0684) | 0.7083($\pm$0.0387) |
| 50% | 0.6983($\pm$0.0359) | 0.6897($\pm$0.0277) | 0.6940($\pm$0.0154) | 0.3387($\pm$0.0268) | 0.7441($\pm$0.0176) |
| 60% | 0.7322($\pm$0.0444) | 0.6704($\pm$0.0546) | 0.7013($\pm$0.0169) | 0.3520($\pm$0.0327) | 0.7613($\pm$0.0238) |
| 70% | 0.7851($\pm$0.0380) | 0.6486($\pm$0.0342) | 0.7169($\pm$0.0116) | 0.3842($\pm$0.0206) | 0.7721($\pm$0.0137) |
| 80% | 0.8026($\pm$0.0393) | 0.6434($\pm$0.0504) | 0.7230($\pm$0.0125) | 0.4050($\pm$0.0239) | 0.7793($\pm$0.0161) |
| 90% | 0.7926(0$\pm$.0287) | 0.6957($\pm$0.0372) | 0.7441($\pm$0.0125) | 0.4488($\pm$0.0243) | 0.8082($\pm$0.0083) |
| 100% | 0.8148($\pm$0.0337) | 0.7258($\pm$0.0309) | 0.7703($\pm$0.0078) | 0.5051($\pm$0.0149) | 0.8472($\pm$0.0088) |

Supplementary Table S9. **Performances of 10-fold stratified cross-validation test based on the antifungal training dataset.**

| **CD-HIT** | **Sensitivity** | **Specificity** | **Accuracy** | **MCC** | **AUC** |
| --- | --- | --- | --- | --- | --- |
| 40% | 0.6795($\pm$0.0826) | 0.7194($\pm$0.0695) | 0.6994($\pm$0.0187) | 0.3687($\pm$0.0343) | 0.7725($\pm$0.0334) |
| 50% | 0.7292($\pm$0.0412) | 0.7171($\pm$0.0622) | 0.7232($\pm$0.0151) | 0.3964($\pm$0.0358) | 0.7871($\pm$0.0215) |
| 60% | 0.7147($\pm$0.0331) | 0.7438($\pm$0.0414) | 0.7293($\pm$0.0230) | 0.4021($\pm$0.0470) | 0.7938($\pm$0.0249) |
| 70% | 0.7352($\pm$0.0399) | 0.7295($\pm$0.0396) | 0.7323($\pm$0.0091) | 0.4071($\pm$0.0181) | 0.8039($\pm$0.0110) |
| 80% | 0.7256($\pm$0.0576) | 0.7403($\pm$0.0560) | 0.7329($\pm$0.0220) | 0.4159($\pm$0.0405) | 0.8086($\pm$0.0225) |
| 90% | 0.7658($\pm$0.0642) | 0.7523($\pm$0.0550) | 0.7590($\pm$0.0148) | 0.4596($\pm$0.0288) | 0.8351($\pm$0.0161) |
| 100% | 0.7756($\pm$0.0282) | 0.8155($\pm$0.0261) | 0.7956($\pm$0.0115) | 0.5354($\pm$0.0249) | 0.8713($\pm$0.0089) |

Supplementary Table S10. **Performances of 10-fold stratified cross-validation test based on the antiviral training dataset.**

| **CD-HIT** | **Sensitivity** | **Specificity** | **Accuracy** | **MCC** | **AUC** |
| --- | --- | --- | --- | --- | --- |
| 40% | 0.6222($\pm$0.1410) | 0.6201($\pm$0.1288) | 0.6212($\pm$0.0547) | 0.2198($\pm$0.1004) | 0.6532($\pm$0.0521) |
| 50% | 0.5415($\pm$0.0838) | 0.6914($\pm$0.0786) | 0.6165($\pm$0.0237) | 0.2138($\pm$0.0446) | 0.6597($\pm$0.0264) |
| 60% | 0.6345($\pm$0.0661) | 0.5546($\pm$0.0650) | 0.5945($\pm$0.0146) | 0.1601($\pm$0.0254) | 0.6257($\pm$0.0165) |
| 70% | 0.6318($\pm$0.1234) | 0.6247($\pm$0.1324) | 0.6282($\pm$0.0127) | 0.2243($\pm$0.0269) | 0.6878($\pm$0.0152) |
| 80% | 0.6518($\pm$0.1256) | 0.6375($\pm$0.1206) | 0.6447($\pm$0.0137) | 0.2502($\pm$0.0259) | 0.7091($\pm$0.0151) |
| 90% | 0.6550($\pm$0.0559) | 0.7434($\pm$0.0419) | 0.6992($\pm$0.0156) | 0.3463($\pm$0.0249) | 0.7792($\pm$0.0148) |
| 100% | 0.7072($\pm$0.0300) | 0.8074($\pm$0.0375) | 0.7573($\pm$0.0136) | 0.4587($\pm$0.0348) | 0.8400($\pm$0.0105) |

Supplementary Table S11. **Performances of 10-fold stratified cross-validation test based on the targeting mammalian cells training dataset.**

| **CD-HIT** | **Sensitivity** | **Specificity** | **Accuracy** | **MCC** | **AUC** |
| --- | --- | --- | --- | --- | --- |
| 40% | 0.7524($\pm$0.0703) | 0.7902($\pm$0.0726) | 0.7713($\pm$0.0398) | 0.4231($\pm$0.0810) | 0.8442($\pm$0.0404) |
| 50% | 0.7643($\pm$0.0653) | 0.7644($\pm$0.0514) | 0.7643($\pm$0.0251) | 0.3977($\pm$0.0395) | 0.8242($\pm$0.0256) |
| 60% | 0.7476($\pm$0.0395) | 0.7463($\pm$0.0387) | 0.7470($\pm$0.0129) | 0.3661($\pm$0.0246) | 0.8135($\pm$0.0162) |
| 70% | 0.7723($\pm$0.0365) | 0.7237($\pm$0.0254) | 0.7480($\pm$0.0228) | 0.3670($\pm$0.0358) | 0.8057($\pm$0.0233) |
| 80% | 0.7706($\pm$0.0315) | 0.7263($\pm$0.0166) | 0.7485($\pm$0.0168) | 0.3787($\pm$0.0264) | 0.8083($\pm$0.0144) |
| 90% | 0.7913($\pm$0.0500) | 0.7312($\pm$0.0294) | 0.7612($\pm$0.0142) | 0.4043($\pm$0.0172) | 0.8214($\pm$0.0077) |
| 100% | 0.7984($\pm$0.0187) | 0.7731($\pm$0.0170) | 0.7857($\pm$0.0087) | 0.4562($\pm$0.0167) | 0.8549($\pm$0.0100) |

Supplementary Table S12. **Performances of 10-fold stratified cross-validation test based on the anti-HIV training dataset.**

| **CD-HIT** | **Sensitivity** | **Specificity** | **Accuracy** | **MCC** | **AUC** |
| --- | --- | --- | --- | --- | --- |
| 40% | 0.6033($\pm$0.3246) | 0.7810($\pm$0.1417) | 0.6921($\pm$0.1088) | 0.2329($\pm$0.1118) | 0.8111($\pm$0.0819) |
| 50% | 0.6479($\pm$0.1709) | 0.7294($\pm$0.0870) | 0.6886($\pm$0.0512) | 0.1799($\pm$0.0404) | 0.7529($\pm$0.0546) |
| 60% | 0.5142($\pm$0.3153) | 0.6569($\pm$0.3252) | 0.5855($\pm$0.0370) | 0.0974($\pm$0.0481) | 0.6937($\pm$0.0508) |
| 70% | 0.5815($\pm$0.2360) | 0.6694($\pm$0.2813) | 0.6254($\pm$0.0445) | 0.1248($\pm$0.0523) | 0.7297($\pm$0.0414) |
| 80% | 0.5544($\pm$0.2488) | 0.7871($\pm$0.2219) | 0.6708($\pm$0.0643) | 0.1813($\pm$0.0618) | 0.7729($\pm$0.0447) |
| 90% | 0.6633($\pm$0.1279) | 0.8322($\pm$0.1076) | 0.7478($\pm$0.0343) | 0.2515($\pm$0.0631) | 0.8299($\pm$0.0280) |
| 100% | 0.7153($\pm$0.1190) | 0.8516($\pm$0.0735) | 0.7835($\pm$0.0281) | 0.2924($\pm$0.0752) | 0.8619($\pm$0.0351) |

Supplementary Table S13. **Performances of 10-fold stratified cross-validation test based on the antibiofilm training dataset.**

| **CD-HIT** | **Sensitivity** | **Specificity** | **Accuracy** | **MCC** | **AUC** |
| --- | --- | --- | --- | --- | --- |
| 40% | 0.5262($\pm$0.1648) | 0.9671($\pm$0.0176) | 0.7466($\pm$0.0820) | 0.4106($\pm$0.1514) | 0.8919($\pm$0.0811) |
| 50% | 0.5318($\pm$0.1698) | 0.9658($\pm$0.0135) | 0.7488($\pm$0.0841) | 0.3880($\pm$0.1209) | 0.8770($\pm$0.0877) |
| 60% | 0.7618($\pm$0.0869) | 0.8561($\pm$0.0367) | 0.8090($\pm$0.0482) | 0.2857($\pm$0.0608) | 0.8837($\pm$0.0375) |
| 70% | 0.6926($\pm$0.0970) | 0.8670($\pm$0.0168) | 0.7798($\pm$0.0512) | 0.2473($\pm$0.0494) | 0.8548($\pm$0.0525) |
| 80% | 0.6057($\pm$0.0927) | 0.8970($\pm$0.0178) | 0.7513($\pm$0.0484) | 0.2371($\pm$0.0510) | 0.8431($\pm$0.0511) |
| 90% | 0.6132($\pm$0.1568) | 0.8903($\pm$0.0346) | 0.7518($\pm$0.0662) | 0.2131($\pm$0.0421) | 0.8477($\pm$0.0311) |
| 100% | 0.5540($\pm$0.0716) | 0.9294($\pm$0.0222) | 0.7417($\pm$0.0374) | 0.2244($\pm$0.0465) | 0.8398($\pm$0.0262) |

Supplementary Table S14. **Performances of 10-fold stratified cross-validation test based on the anticancer training dataset.**

| **CD-HIT** | **Sensitivity** | **Specificity** | **Accuracy** | **MCC** | **AUC** |
| --- | --- | --- | --- | --- | --- |
| 40% | 0.7438($\pm$0.0480) | 0.6836($\pm$0.0677) | 0.7137($\pm$0.0221) | 0.4151($\pm$0.0437) | 0.7821($\pm$0.0236) |
| 50% | 0.6502($\pm$0.0627) | 0.6193($\pm$0.0665) | 0.6348($\pm$0.0256) | 0.2580($\pm$0.0501) | 0.6999($\pm$0.0265) |
| 60% | 0.6424($\pm$0.0578) | 0.6204($\pm$0.0499) | 0.6314($\pm$0.0166) | 0.2412($\pm$0.0312) | 0.6755($\pm$0.0158) |
| 70% | 0.6805($\pm$0.0451) | 0.5664($\pm$0.0436) | 0.6235($\pm$0.0196) | 0.2199($\pm$0.0348) | 0.6680($\pm$0.0211) |
| 80% | 0.7029($\pm$0.0521) | 0.5687($\pm$0.0434) | 0.6358($\pm$0.0188) | 0.2378($\pm$0.0326) | 0.6789($\pm$0.0172) |
| 90% | 0.7051($\pm$0.0595) | 0.6023($\pm$0.0536) | 0.6537($\pm$0.0114) | 0.2597($\pm$0.0191) | 0.7092($\pm$0.0125) |
| 100% | 0.7609($\pm$0.0533) | 0.6106($\pm$0.0497) | 0.6857($\pm$0.0131) | 0.2955($\pm$0.0198) | 0.7558($\pm$0.0123) |

Supplementary Table S15. **Performances of 10-fold stratified cross-validation test based on the hemolytic training dataset.**

| **CD-HIT** | **Sensitivity** | **Specificity** | **Accuracy** | **MCC** | **AUC** |
| --- | --- | --- | --- | --- | --- |
| 40% | 0.7824($\pm$0.1207) | 0.8703($\pm$0.0518) | 0.8263($\pm$0.0463) | 0.4638($\pm$0.0656) | 0.9014($\pm$0.0490) |
| 50% | 0.8264($\pm$0.0741) | 0.8069($\pm$0.0642) | 0.8167($\pm$0.0286) | 0.3946($\pm$0.0517) | 0.8842($\pm$0.0268) |
| 60% | 0.7867($\pm$0.0516) | 0.8074($\pm$0.0253) | 0.7970($\pm$0.0198) | 0.3596($\pm$0.0230) | 0.8684($\pm$0.0166) |
| 70% | 0.8076($\pm$0.0761) | 0.7875($\pm$0.0401) | 0.7975($\pm$0.0249) | 0.3594($\pm$0.0253) | 0.8634($\pm$0.0208) |
| 80% | 0.8068($\pm$0.0443) | 0.8046($\pm$0.0192) | 0.8057($\pm$0.0242) | 0.3934($\pm$0.0351) | 0.8701($\pm$0.0194) |
| 90% | 0.8258($\pm$0.0394) | 0.8156($\pm$0.0280) | 0.8207($\pm$0.0173) | 0.4298($\pm$0.0293) | 0.8866($\pm$0.0128) |
| 100% | 0.8417($\pm$0.0299) | 0.8523($\pm$0.0295) | 0.8470($\pm$0.0117) | 0.5054($\pm$0.0349) | 0.9169($\pm$0.0130) |

Supplementary Table S16. **Performances of 10-fold stratified cross-validation test based on the chemotactic training dataset.**

| **CD-HIT** | **Sensitivity** | **Specificity** | **Accuracy** | **MCC** | **AUC** |
| --- | --- | --- | --- | --- | --- |
| 40% | 0.5700($\pm$0.2786) | 0.9135($\pm$0.0737) | 0.7418($\pm$0.1216) | 0.2694($\pm$0.1511) | 0.8579($\pm$0.0783) |
| 50% | 0.6433($\pm$0.2161) | 0.8351($\pm$0.0662) | 0.7392($\pm$0.0932) | 0.1490($\pm$0.0543) | 0.8529($\pm$0.0607) |
| 60% | 0.5900($\pm$0.2201) | 0.8423($\pm$0.1615) | 0.7161($\pm$0.0988) | 0.1671($\pm$0.1198) | 0.8730($\pm$0.0747) |
| 70% | 0.7310($\pm$0.2119) | 0.8545($\pm$0.0933) | 0.7927($\pm$0.1071) | 0.1915($\pm$0.1568) | 0.8753($\pm$0.0762) |
| 80% | 0.6762($\pm$0.2509) | 0.7815($\pm$0.2545) | 0.7288($\pm$0.0837) | 0.1657($\pm$0.0877) | 0.8637($\pm$0.0438) |
| 90% | 0.5619($\pm$0.2723) | 0.9501($\pm$0.0318) | 0.7560($\pm$0.1315) | 0.1958($\pm$0.1073) | 0.8727($\pm$0.1232) |
| 100% | 0.6548($\pm$0.2188) | 0.8816($\pm$0.1224) | 0.7682($\pm$0.0892) | 0.1709($\pm$0.0863) | 0.8880($\pm$0.0664) |

Supplementary Table S17. **Performances of 10-fold stratified cross-validation test based on the anti-MRSA training dataset.**

| **CD-HIT** | **Sensitivity** | **Specificity** | **Accuracy** | **MCC** | **AUC** |
| --- | --- | --- | --- | --- | --- |
| 40% | 0.6929($\pm$0.2169) | 0.8553($\pm$0.1790) | 0.7741($\pm$0.0849) | 0.3573($\pm$0.1393) | 0.9157($\pm$0.0433) |
| 50% | 0.5720($\pm$0.2692) | 0.8724($\pm$0.0922) | 0.7222($\pm$0.0943) | 0.2293($\pm$0.0436) | 0.8795($\pm$0.0493) |
| 60% | 0.6333($\pm$0.2782) | 0.8109($\pm$0.1366) | 0.7221($\pm$0.0951) | 0.1774($\pm$0.0619) | 0.8508($\pm$0.0474) |
| 70% | 0.6581($\pm$0.1464) | 0.8233($\pm$0.0867) | 0.7407($\pm$0.0573) | 0.1846($\pm$0.0564) | 0.8251($\pm$0.0682) |
| 80% | 0.6219($\pm$0.2400) | 0.8049($\pm$0.1088) | 0.7134($\pm$0.0740) | 0.1431($\pm$0.0365) | 0.8196($\pm$0.0469) |
| 90% | 0.7637($\pm$0.1114) | 0.7553($\pm$0.0915) | 0.7595($\pm$0.0274) | 0.1511($\pm$0.0225) | 0.8338($\pm$0.0410) |
| 100% | 0.6888($\pm$0.2032) | 0.7818($\pm$0.0969) | 0.7353($\pm$0.0568) | 0.1294($\pm$0.0133) | 0.8224($\pm$0.0482) |

Supplementary Table S18. **Performances of 10-fold stratified cross-validation test based on the anti-TB training dataset.**

| **CD-HIT** | **Sensitivity** | **Specificity** | **Accuracy** | **MCC** | **AUC** |
| --- | --- | --- | --- | --- | --- |
| 40% | 0.5800($\pm$0.3156) | 0.8616($\pm$0.0929) | 0.7208($\pm$0.1276) | 0.2264($\pm$0.1148) | 0.8447($\pm$0.1010) |
| 50% | 0.4762($\pm$0.2065) | 0.8198($\pm$0.1263) | 0.6480($\pm$0.0892) | 0.1200($\pm$0.0902) | 0.7824($\pm$0.0981) |
| 60% | 0.4982($\pm$0.2353) | 0.7639($\pm$0.1814) | 0.6311($\pm$0.1068) | 0.0735($\pm$0.0498) | 0.7175($\pm$0.0760) |
| 70% | 0.3786($\pm$0.4163) | 0.7056($\pm$0.3248) | 0.5421($\pm$0.0603) | 0.0188($\pm$0.0286) | 0.7087($\pm$0.0752) |
| 80% | 0.5722($\pm$0.2930) | 0.7076($\pm$0.2164) | 0.6399($\pm$0.0714) | 0.0730($\pm$0.0369) | 0.7411($\pm$0.1164) |
| 90% | 0.3700($\pm$0.2725) | 0.8668($\pm$0.1063) | 0.6184($\pm$0.0941) | 0.0639($\pm$0.0430) | 0.7353($\pm$0.0757) |
| 100% | 0.4764($\pm$0.1724) | 0.8755($\pm$0.1265) | 0.6759($\pm$0.0440) | 0.1146($\pm$0.0536) | 0.7587($\pm$0.0658) |

Supplementary Table S19. **Performances of 10-fold stratified cross-validation test based on the antiparasitic training dataset.**

| **CD-HIT** | **Sensitivity** | **Specificity** | **Accuracy** | **MCC** | **AUC** |
| --- | --- | --- | --- | --- | --- |
| 40% | 0.5878($\pm$0.2000) | 0.7673($\pm$0.1677) | 0.6775($\pm$0.0690) | 0.1993($\pm$0.0778) | 0.7797($\pm$0.0924) |
| 50% | 0.6257($\pm$0.1735) | 0.7831($\pm$0.1077) | 0.7044($\pm$0.0386) | 0.1851($\pm$0.0209) | 0.7835($\pm$0.0535) |
| 60% | 0.7161($\pm$0.2213) | 0.6598($\pm$0.1240) | 0.6879($\pm$0.0711) | 0.1320($\pm$0.0481) | 0.7700($\pm$0.0740) |
| 70% | 0.6359($\pm$0.2164) | 0.7464($\pm$0.1293) | 0.6912($\pm$0.0604) | 0.1426($\pm$0.0315) | 0.7796($\pm$0.0340) |
| 80% | 0.7208($\pm$0.1322) | 0.7116($\pm$0.0973) | 0.7162($\pm$0.0484) | 0.1480($\pm$0.0336) | 0.7818($\pm$0.0558) |
| 90% | 0.6481($\pm$0.2272) | 0.7818($\pm$0.1489) | 0.7150($\pm$0.0617) | 0.1658($\pm$0.0425) | 0.7891($\pm$0.0343) |
| 100% | 0.6069($\pm$0.0819) | 0.8564($\pm$0.0328) | 0.7316($\pm$0.0359) | 0.1778($\pm$0.0290) | 0.8071($\pm$0.0350) |

Supplementary Table S20. **Performances of 10-fold stratified cross-validation test based on the cytotoxic training dataset.**

| **CD-HIT** | **Sensitivity** | **Specificity** | **Accuracy** | **MCC** | **AUC** |
| --- | --- | --- | --- | --- | --- |
| 40% | 0.5250($\pm$0.2765) | 0.8632($\pm$0.1748) | 0.6941($\pm$0.0908) | 0.2106($\pm$0.1127) | 0.9042($\pm$0.0674) |
| 50% | 0.6567($\pm$0.2868) | 0.8581($\pm$0.1356) | 0.7574($\pm$0.1263) | 0.1943($\pm$0.1114) | 0.9069($\pm$0.0502) |
| 60% | 0.6321($\pm$0.2514) | 0.8694($\pm$0.1107) | 0.7508($\pm$0.0898) | 0.1639($\pm$0.0470) | 0.8739($\pm$0.0535) |
| 70% | 0.5833($\pm$0.2472) | 0.8764($\pm$0.0834) | 0.7299($\pm$0.0938) | 0.1443($\pm$0.0440) | 0.8799($\pm$0.0476) |
| 80% | 0.6861($\pm$0.2201) | 0.8477($\pm$0.0953) | 0.7669($\pm$0.0885) | 0.1582($\pm$0.0633) | 0.8753($\pm$0.0475) |
| 90% | 0.6809($\pm$0.1878) | 0.8493($\pm$0.0731) | 0.7651($\pm$0.0687) | 0.1469($\pm$0.0334) | 0.8861($\pm$0.0390) |
| 100% | 0.6885($\pm$0.1237) | 0.9329($\pm$0.0360) | 0.8107($\pm$0.0530) | 0.2400($\pm$0.0685) | 0.9150($\pm$0.0281) |

Supplementary Table S21. **Performances of 10-fold stratified cross-validation test based on the endotoxin training dataset.**

| **CD-HIT** | **Sensitivity** | **Specificity** | **Accuracy** | **MCC** | **AUC** |
| --- | --- | --- | --- | --- | --- |
| 40% | 0.6300($\pm$0.3945) | 0.8170($\pm$0.1828) | 0.7235($\pm$0.1549) | 0.1801($\pm$0.1416) | 0.8678($\pm$0.0923) |
| 50% | 0.5200($\pm$0.3816) | 0.7705($\pm$0.1908) | 0.6453($\pm$0.1358) | 0.0752($\pm$0.0771) | 0.8041($\pm$0.0949) |
| 60% | 0.4533($\pm$0.4392) | 0.7180($\pm$0.2471) | 0.5857($\pm$0.1233) | 0.0297($\pm$0.0497) | 0.7739($\pm$0.1039) |
| 70% | 0.7071($\pm$0.1881) | 0.7085($\pm$0.1626) | 0.7078($\pm$0.0537) | 0.0865($\pm$0.0273) | 0.7660($\pm$0.0754) |
| 80% | 0.5048($\pm$0.3447) | 0.7796($\pm$0.2056) | 0.6422($\pm$0.0889) | 0.0595($\pm$0.0335) | 0.7874($\pm$0.0728) |
| 90% | 0.4810($\pm$0.2960) | 0.7612($\pm$0.2258) | 0.6211($\pm$0.0631) | 0.0519($\pm$0.0288) | 0.7712($\pm$0.0761) |
| 100% | 0.6048($\pm$0.2486) | 0.7040($\pm$0.1821) | 0.6544($\pm$0.0775) | 0.0491($\pm$0.0222) | 0.7370($\pm$0.0864) |

Supplementary Table S22. **Performances of 10-fold stratified cross-validation test based on the insecticidal training dataset.**

| **CD-HIT** | **Sensitivity** | **Specificity** | **Accuracy** | **MCC** | **AUC** |
| --- | --- | --- | --- | --- | --- |
| 40% | 0.7418($\pm$0.1573) | 0.6448($\pm$0.1938) | 0.6933($\pm$0.0571) | 0.2170($\pm$0.0760) | 0.7628($\pm$0.0485) |
| 50% | 0.5873($\pm$0.1460) | 0.7040($\pm$0.1172) | 0.6456($\pm$0.0403) | 0.1320($\pm$0.0387) | 0.7265($\pm$0.0431) |
| 60% | 0.5400($\pm$0.2809) | 0.7301($\pm$0.1432) | 0.6351($\pm$0.0770) | 0.0993($\pm$0.0490) | 0.7213($\pm$0.0617) |
| 70% | 0.6074($\pm$0.2527) | 0.6947($\pm$0.2100) | 0.6510($\pm$0.0435) | 0.1250($\pm$0.0405) | 0.7448($\pm$0.0609) |
| 80% | 0.6198($\pm$0.2504) | 0.6453($\pm$0.2453) | 0.6325($\pm$0.0577) | 0.1011($\pm$0.0390) | 0.7263($\pm$0.0633) |
| 90% | 0.6663($\pm$0.2067) | 0.6720($\pm$0.2193) | 0.6692($\pm$0.0391) | 0.1286($\pm$0.0582) | 0.7693($\pm$0.0584) |
| 100% | 0.6397($\pm$0.2017) | 0.7985($\pm$0.1178) | 0.7191($\pm$0.0566) | 0.1608($\pm$0.0474) | 0.8232($\pm$0.0490) |

Supplementary Table S23. **Performances of 10-fold stratified cross-validation test based on the antimalarial training dataset.**

| **CD-HIT** | **Sensitivity** | **Specificity** | **Accuracy** | **MCC** | **AUC** |
| --- | --- | --- | --- | --- | --- |
| 40% | 0.6417($\pm$0.3271) | 0.6952($\pm$0.2142) | 0.6684($\pm$0.1077) | 0.1074($\pm$0.0723) | 0.8071($\pm$0.1053) |
| 50% | 0.4300($\pm$0.2909) | 0.8495($\pm$0.1133) | 0.6397($\pm$0.1124) | 0.0864($\pm$0.0710) | 0.7559($\pm$0.0999) |
| 60% | 0.4200($\pm$0.3842) | 0.8221($\pm$0.2098) | 0.6210($\pm$0.1202) | 0.0642($\pm$0.0714) | 0.7857($\pm$0.0749) |
| 70% | 0.4600($\pm$0.3105) | 0.7757($\pm$0.1862) | 0.6179($\pm$0.1084) | 0.0576($\pm$0.0699) | 0.7601($\pm$0.1028) |
| 80% | 0.4600($\pm$0.4104) | 0.8137($\pm$0.2032) | 0.6369($\pm$0.1353) | 0.0589($\pm$0.0699) | 0.7641($\pm$0.1039) |
| 90% | 0.4933($\pm$0.2670) | 0.8696($\pm$0.0884) | 0.6815($\pm$0.0956) | 0.0838($\pm$0.0280) | 0.8021($\pm$0.0821) |
| 100% | 0.4900($\pm$0.1886) | 0.8747($\pm$0.0514) | 0.6823($\pm$0.0799) | 0.0648($\pm$0.0248) | 0.7948($\pm$0.0670) |

Supplementary Table S24. **Performances of 10-fold stratified cross-validation test based on the anticandidal training dataset.**

| **CD-HIT** | **Sensitivity** | **Specificity** | **Accuracy** | **MCC** | **AUC** |
| --- | --- | --- | --- | --- | --- |
| 40% | 0.5764($\pm$0.2301) | 0.7922($\pm$0.1305) | 0.6843($\pm$0.0686) | 0.2248($\pm$0.0708) | 0.7956($\pm$0.0589) |
| 50% | 0.6885($\pm$0.1775) | 0.7905($\pm$0.1089) | 0.7395($\pm$0.0596) | 0.2640($\pm$0.0679) | 0.8254($\pm$0.0293) |
| 60% | 0.7589($\pm$0.1019) | 0.7672($\pm$0.0501) | 0.7630($\pm$0.0330) | 0.2392($\pm$0.0257) | 0.8296($\pm$0.0314) |
| 70% | 0.6980($\pm$0.1511) | 0.7751($\pm$0.0865) | 0.7366($\pm$0.0542) | 0.2226($\pm$0.0556) | 0.8284($\pm$0.0361) |
| 80% | 0.7435($\pm$0.0621) | 0.7748($\pm$0.0530) | 0.7591($\pm$0.0325) | 0.2353($\pm$0.0432) | 0.8447($\pm$0.0244) |
| 90% | 0.7204($\pm$0.1611) | 0.7869($\pm$0.0700) | 0.7536($\pm$0.0567) | 0.2238($\pm$0.0418) | 0.8436($\pm$0.0405) |
| 100% | 0.7500($\pm$0.0999) | 0.8177($\pm$0.0655) | 0.7838($\pm$0.0347) | 0.2552($\pm$0.0340) | 0.8704($\pm$0.0255) |

Supplementary Table S25. **Performances of 10-fold stratified cross-validation test based on the antiplasmodial training dataset.**

| **CD-HIT** | **Sensitivity** | **Specificity** | **Accuracy** | **MCC** | **AUC** |
| --- | --- | --- | --- | --- | --- |
| 40% | 0.4000($\pm$0.3742) | 0.8882($\pm$0.1762) | 0.6441($\pm$0.1786) | 0.1717($\pm$0.2400) | 0.8794($\pm$0.1509) |
| 50% | 0.4167($\pm$0.4425) | 0.8458($\pm$0.1666) | 0.6312($\pm$0.1520) | 0.0411($\pm$0.0512) | 0.8154($\pm$0.1469) |
| 60% | 0.3833($\pm$0.4349) | 0.8240($\pm$0.2261) | 0.6037($\pm$0.1494) | 0.0449($\pm$0.0708) | 0.7256($\pm$0.1857) |
| 70% | 0.3500($\pm$0.3023) | 0.9694($\pm$0.0272) | 0.6597($\pm$0.1487) | 0.1124($\pm$0.1018) | 0.7908($\pm$0.1586) |
| 80% | 0.3500($\pm$0.3371) | 0.9793($\pm$0.0125) | 0.6647($\pm$0.1688) | 0.1424($\pm$0.1597) | 0.8025($\pm$0.1509) |
| 90% | 0.4083($\pm$0.2281) | 0.9190($\pm$0.0846) | 0.6637($\pm$0.1069) | 0.1183($\pm$0.0957) | 0.8153($\pm$0.1031) |
| 100% | 0.5917($\pm$0.2750) | 0.9221($\pm$0.0716) | 0.7569($\pm$0.1439) | 0.1344($\pm$0.0921) | 0.8505($\pm$0.1210) |

Supplementary Table S26. **Performances of 10-fold stratified cross-validation test based on the antiprotozoal training dataset.**

| **CD-HIT** | **Sensitivity** | **Specificity** | **Accuracy** | **MCC** | **AUC** |
| --- | --- | --- | --- | --- | --- |
| 40% | 0.6000($\pm$0.3000) | 0.7721($\pm$0.3781) | 0.6861($\pm$0.1414) | 0.2067($\pm$0.1741) | 0.8794($\pm$0.0777) |
| 50% | 0.4000($\pm$0.3512) | 0.9116($\pm$0.1422) | 0.6558($\pm$0.1802) | 0.1223($\pm$0.1382) | 0.8869($\pm$0.0886) |
| 60% | 0.3833($\pm$0.2794) | 0.9322($\pm$0.0784) | 0.6578($\pm$0.1182) | 0.0981($\pm$0.0923) | 0.8721($\pm$0.0884) |
| 70% | 0.3583($\pm$0.3801) | 0.9396($\pm$0.0557) | 0.6490($\pm$0.1645) | 0.0667($\pm$0.0687) | 0.8780($\pm$0.0758) |
| 80% | 0.4083($\pm$0.2512) | 0.9608($\pm$0.0391) | 0.6846($\pm$0.1225) | 0.1584($\pm$0.1160) | 0.9019($\pm$0.0787) |
| 90% | 0.5000($\pm$0.3708) | 0.9430($\pm$0.0646) | 0.7215($\pm$0.1671) | 0.1140($\pm$0.0961) | 0.8676($\pm$0.0870) |
| 100% | 0.7650($\pm$0.2730) | 0.8696($\pm$0.0990) | 0.8173($\pm$0.1301) | 0.1265($\pm$0.0774) | 0.8921($\pm$0.0875) |

Supplementary Table S27. **Performances of 10-fold stratified cross-validation test based on the anuran defense training dataset.**

| **CD-HIT** | **Sensitivity** | **Specificity** | **Accuracy** | **MCC** | **AUC** |
| --- | --- | --- | --- | --- | --- |
| 40% | 0.8405($\pm$0.1860) | 0.8657($\pm$0.1699) | 0.8531($\pm$0.0920) | 0.4317($\pm$0.1563) | 0.9691($\pm$0.0272) |
| 50% | 0.7264($\pm$0.2721) | 0.9158($\pm$0.0456) | 0.8211($\pm$0.1197) | 0.3300($\pm$0.1256) | 0.9502($\pm$0.0297) |
| 60% | 0.8650($\pm$0.1045) | 0.8803($\pm$0.0605) | 0.8726($\pm$0.0423) | 0.3709($\pm$0.0765) | 0.9343($\pm$0.0199) |
| 70% | 0.8643($\pm$0.0923) | 0.9078($\pm$0.0281) | 0.8861($\pm$0.0387) | 0.4274($\pm$0.0441) | 0.9449($\pm$0.0202) |
| 80% | 0.8852($\pm$0.0411) | 0.9274($\pm$0.0142) | 0.9063($\pm$0.0193) | 0.5018($\pm$0.0396) | 0.9521($\pm$0.0095) |
| 90% | 0.9142($\pm$0.0373) | 0.9262($\pm$0.0133) | 0.9202($\pm$0.0180) | 0.5172($\pm$0.0382) | 0.9631($\pm$0.0124) |
| 100% | 0.9232($\pm$0.0421) | 0.9283($\pm$0.0112) | 0.9257($\pm$0.0223) | 0.5300($\pm$0.0418) | 0.9717($\pm$0.0105) |

Supplementary Table S28. **Performances of different computational approaches for predicting AMP functional activities based on imbalanced independent test datasets.**

| **Activity** | **Method** | **Sensitivity** | **Specificity** | **Accuracy** | **MCC** | **AUC** |
| --- | --- | --- | --- | --- | --- | --- |
| antibacterial | AMAP | 0.8785 | 0.3633 | 0.6209 | 0.2855 | 0.6209 |
|  | AMPDiscover-RF | **0.8798** | 0.4518 | 0.6658 | 0.3714 | 0.6328 |
|  | AMPDiscover-RNN | 0.8750 | 0.4495 | 0.6623 | 0.3629 | 0.6623 |
|  | iAMP-CA2L | 0.6852 | 0.3148 | 0.5000 | 0.0000 | 0.5000 |
|  | iAMPCN | 0.7923 | **0.6118** | **0.7020** | **0.4122** | **0.7625** |
| antifungal | AMAP | **0.8228** | 0.2922 | 0.5575 | 0.1038 | 0.5575 |
|  | AMPDiscover-RF | 0.7851 | 0.3316 | 0.5583 | 0.1011 | 0.5522 |
|  | AMPDiscover-RNN | 0.7895 | 0.2977 | 0.5436 | 0.0777 | 0.5436 |
|  | AMPfun | 0.5746 | 0.5884 | 0.5815 | 0.1313 | 0.6157 |
|  | dbAMP | 0.8044 | 0.3557 | 0.5801 | 0.1370 | 0.6073 |
|  | iAMP-CA2L | 0.2140 | 0.7783 | 0.4962 | -0.0074 | 0.4962 |
|  | iAMP-RAAC | 0.6763 | 0.5071 | 0.5917 | 0.1471 | 0.5917 |
|  | iAMPCN | 0.5368 | **0.8104** | **0.6736** | **0.3171** | **0.7466** |
| antiviral | AMAP | 0.2336 | 0.4553 | 0.3445 | -0.2571 | 0.3445 |
|  | AMPDiscover-RF | 0.5432 | 0.2818 | 0.4125 | -0.1548 | 0.5031 |
|  | AMPDiscover-RNN | 0.5683 | 0.2974 | 0.4328 | -0.1182 | 0.4328 |
|  | AMPfun | 0.7502 | 0.4717 | 0.6110 | 0.1854 | 0.6714 |
|  | dbAMP | **0.8618** | 0.2494 | 0.5556 | 0.1099 | 0.5992 |
|  | iAMP-CA2L | 0.0243 | **0.9766** | 0.5004 | 0.0022 | 0.5004 |
|  | iAMP-RAAC | 0.6653 | 0.5830 | 0.6242 | 0.2054 | 0.6242 |
|  | iAMPCN | 0.7308 | 0.8651 | **0.7979** | **0.5575** | **0.8888** |
| anticancer | AMAP | 0.4118 | 0.3676 | 0.3897 | -0.1664 | 0.3897 |
|  | AMPfun | 0.4172 | 0.4617 | 0.4395 | -0.0897 | 0.4335 |
|  | dbAMP | 0.6344 | 0.3181 | 0.4762 | -0.0375 | 0.4747 |
|  | iAMP-RAAC | 0.1172 | **0.8640** | 0.4906 | -0.0205 | 0.4906 |
|  | iAMPCN | **0.7667** | 0.6345 | **0.7006** | **0.2999** | **0.7641** |
| anti-Gram positive | AMPfun | 0.8865 | 0.2907 | 0.5886 | 0.1822 | 0.6224 |
|  | dbAMP | 0.8265 | 0.3266 | 0.5766 | 0.1495 | 0.6168 |
|  | iAMP-RAAC | **0.8879** | 0.3007 | 0.5943 | 0.1920 | 0.5943 |
|  | iAMPCN | 0.7995 | **0.5510** | **0.6752** | **0.3104** | **0.7257** |
| anti-Gram negative | AMPfun | **0.8868** | 0.3114 | 0.5991 | 0.2057 | 0.6324 |
|  | dbAMP | 0.8515 | 0.3232 | 0.5873 | 0.1772 | 0.6226 |
|  | iAMP-RAAC | 0.8582 | 0.3334 | 0.5958 | 0.1932 | 0.5958 |
|  | iAMPCN | 0.8058 | **0.5426** | **0.6742** | **0.3171** | **0.7270** |
| Anti-mammalian cells | AMPfun | 0.3574 | **0.7864** | 0.5719 | 0.1194 | 0.6267 |
|  | dbAMP | 0.6486 | 0.4716 | 0.5601 | 0.0849 | 0.5750 |
|  | iAMP-RAAC | 0.1769 | 0.9089 | 0.5429 | 0.0990 | 0.5429 |
|  | iAMPCN | **0.7942** | 0.6256 | **0.7099** | **0.2979** | **0.7684** |
| anti-HIV | AMAP | 0.2830 | 0.5022 | 0.3926 | -0.0700 | 0.3926 |
|  | iAMP-CA2L | 0.0063 | **0.9882** | 0.4972 | -0.0084 | 0.4972 |
|  | iAMPCN | **0.7421** | 0.8736 | **0.8079** | **0.2863** | **0.8690** |
| anti-MRSA | iAMP-CA2L | 0.4151 | **0.9798** | 0.6975 | **0.2398** | 0.6975 |
|  | iAMPCN | **0.8113** | 0.7821 | **0.7967** | 0.1312 | **0.8184** |
| antiparasitic | AMAP | 0.1609 | 0.5807 | 0.3708 | -0.0636 | 0.3708 |
|  | AMPDiscover-RF | **0.7241** | 0.3133 | 0.5187 | 0.0098 | 0.6001 |
|  | AMPDiscover-RNN | 0.7011 | 0.3168 | 0.5090 | 0.0047 | 0.5090 |
|  | AMPfun | 0.4368 | 0.4646 | 0.4507 | -0.0240 | 0.4633 |
|  | iAMP-CA2L | 0.0230 | **0.9923** | 0.5077 | 0.0210 | 0.5077 |
|  | iAMP-RAAC | 0.1839 | 0.8648 | 0.5243 | 0.0172 | 0.5243 |
|  | iAMPCN | 0.6322 | 0.7572 | **0.6947** | **0.1093** | **0.7899** |
| antibiofilm | AMAP | 0.4595 | 0.5940 | 0.5267 | 0.0122 | 0.5267 |
|  | iAMP-CA2L | 0.0811 | **0.9953** | 0.5382 | 0.1140 | 0.5382 |
|  | iAMPCN | **0.8243** | 0.7536 | **0.7890** | **0.1486** | **0.8479** |
| chemotactic | AMAP | 0.1429 | 0.8176 | 0.4802 | -0.0050 | 0.4802 |
|  | iAMP-CA2L | 0.0000 | 0.9938 | 0.4969 | -0.0039 | 0.4969 |
|  | iAMPCN | **0.5000** | **0.9216** | **0.7108** | **0.0763** | **0.7851** |
| endotoxin | iAMP-CA2L | 0.6875 | **0.9898** | **0.8387** | **0.3208** | **0.8387** |
|  | iAMPCN | 0.7500 | 0.6650 | 0.7075 | 0.0453 | 0.7252 |
| insecticidal | AMAP | 0.2289 | 0.8374 | 0.5332 | 0.0212 | 0.5332 |
|  | iAMPCN | **0.7590** | **0.8820** | **0.8205** | **0.2278** | **0.9017** |

Supplementary Table S29. **Performances of different computational approaches based on different datasets.**

| **Method** | **SN** | **SP** | **ACC** | **MCC** | **AUC** |
| --- | --- | --- | --- | --- | --- |
| (A) Performance of shallow and DNN models built and assessed on the Veltri AMP benchmarking dataset | | | | | |
| LogitBoost+RF | **0.956** | 0.956 | **0.9565** | **0.913** | **0.991** |
| LogitBoost+RF | 0.941 | **0.959** | 0.9501 | 0.9 | 0.99 |
| RandomCommittee+RF | 0.935 | 0.947 | 0.941 | 0.882 | 0.986 |
| RF | 0.935 | 0.945 | 0.9403 | 0.881 | 0.985 |
| BERT-based | 0.92 | 0.953 | 0.9368 | 0.874 | - |
| RandomCommittee+RF | 0.927 | 0.938 | 0.9326 | 0.865 | 0.982 |
| ACEP | 0.924 | 0.937 | 0.9304 | 0.861 | 0.978 |
| RF | 0.917 | 0.935 | 0.9263 | 0.853 | 0.98 |
| APIN-fusion | 0.899 | 0.95 | 0.9255 | 0.852 | 0.973 |
| APIN | 0.91 | 0.936 | 0.9241 | 0.849 | 0.972 |
| LogitBoost+RF | 0.904 | 0.935 | 0.9199 | 0.84 | 0.974 |
| RF | 0.902 | 0.931 | 0.9164 | 0.833 | 0.97 |
| RandomCommittee+RF | 0.9 | 0.927 | 0.9136 | 0.828 | 0.971 |
| AMPScanner | 0.899 | 0.921 | 0.9101 | 0.82 | 0.965 |
| iAMPCN | 0.9242 | 0.9284 | 0.9263 | 0.853 | 0.972 |
| (B) Performance of shallow and DNN models built and assessed on the Nishant AVP benchmarking dataset | | | | | |
| RandomCommittee+RF | 0.883 | **0.867** | 0.876 | 0.75 | 0.948 |
| RandomCommittee+RF | 0.9 | 0.844 | 0.876 | 0.75 | 0.961 |
| LogitBoost+RF | 0.9 | 0.844 | 0.876 | 0.75 | 0.947 |
| DeepAVP | 0.9 | 0.844 | 0.876 | 0.75 | - |
| RF | 0.867 | 0.867 | 0.867 | 0.73 | 0.947 |
| LogitBoost+RF | 0.883 | 0.844 | 0.867 | 0.73 | **0.964** |
| BidLSTM | 0.85 | 0.867 | 0.857 | 0.71 | - |
| LogitBoost+RF | 0.867 | 0.822 | 0.848 | 0.69 | 0.957 |
| RandomCommittee+RF | 0.883 | 0.8 | 0.848 | 0.69 | 0.948 |
| RF* | 0.9 | 0.778 | 0.848 | 0.69 | 0.939 |
| RF** | 0.867 | 0.8 | 0.838 | 0.67 | 0.953 |
| UnidLSTM | 0.9 | 0.711 | 0.819 | 0.63 | - |
| MultiLSTM | 0.883 | 0.733 | 0.819 | 0.63 | - |
| DynEvo | **0.917** | 0.733 | 0.838 | 0.67 | - |
| StaEvo | 0.9 | 0.667 | 0.8 | 0.59 | - |
| iAMPCN | 0.9 | **0.867** | **0.8833** | **0.7667** | 0.935 |
| (C) Performance of shallow and DNN models built and assessed on the Nishant AVP benchmarking dataset | | | | | |
| LogitBoost+RF | 0.9 | **1** | 0.95 | 0.905 | 0.98 |
| LogitBoost+RF | 0.9 | **1** | 0.95 | 0.905 | **0.985** |
| DeepAVP | **0.967** | 0.9 | 0.933 | 0.87 | - |
| iAMPCN | **0.967** | 0.95 | **0.9583** | **0.9168** | 0.9844 |
| (D) Performance of shallow and DNN models built and assessed on the Li AVP benchmarking dataset | | | | | |
| RandomCommittee+RF | 0.814 | **0.857** | 0.8229 | 0.576 | 0.891 |
| RandomCommittee+RF | **0.864** | 0.8 | **0.8514** | **0.6** | **0.915** |
| LogitBoost+RF | 0.821 | 0.829 | 0.8229 | 0.563 | 0.906 |
| LogitBoost+RF | 0.836 | 0.8 | 0.8286 | 0.56 | 0.914 |
| DeepAVP | 0.807 | 0.829 | 0.8114 | 0.545 | - |
| RF | 0.786 | **0.857** | 0.8 | 0.542 | 0.904 |
| iAMPCN | 0.8357 | 0.8286 | 0.8321 | 0.5814 | 0.882 |
| (E)Performance of shallow and DNN models built and assessed on the Yan AMP benchmarking dataset | | | | | |
| LogitBoost+RF | **0.883** | 0.883 | **0.883** | **0.766** | **0.932** |
| RandomCommittee+RF | 0.84 | **0.894** | 0.867 | 0.735 | 0.925 |
| LogitBoost+RF | 0.851 | 0.883 | 0.867 | 0.734 | 0.932 |
| RF* | 0.862 | 0.872 | 0.867 | 0.734 | 0.92 |
| RandomCommittee+RF | 0.83 | 0.883 | 0.8564 | 0.714 | 0.922 |
| RF** | 0.84 | 0.862 | 0.8511 | 0.702 | 0.927 |
| BERT-based | 0.851 | 0.777 | 0.8138 | 0.629 | - |
| Deep-AmPEP30 | 0.766 | 0.777 | 0.7713 | 0.543 | 0.853 |
| iAMPCN | 0.7872 | 0.7979 | 0.7926 | 0.5851 | 0.862 |
| (F) Performance of shallow and DNN models built and assessed on the Sharma ABP benchmarking dataset | | | | | |
| LogitBoost+RF | **0.994** | 0.946 | **0.9659** | **0.932** | **0.998** |
| LogitBoost+RF | 0.991 | 0.947 | 0.9649 | 0.93 | **0.998** |
| Deep-ABPpred | 0.962 | 0.955 | 0.958 | 0.914 | - |
| iAMPCN | 0.9263 | **0.9721** | 0.9533 | 0.9034 | 0.990 |
| (G)Performance of shallow and DNN models built and assessed on the Sharma AMP benchmarking dataset | | | | | |
| AniAMPpred | 0.95 | **0.986** | 0.9682 | 0.937 | 0.993 |
| LogitBoost+RF | **0.966** | 0.969 | 0.9674 | 0.935 | **0.995** |
| LogitBoost+RF | 0.958 | 0.967 | 0.9624 | 0.925 | 0.994 |
| iAMPCN | 0.9538 | 0.9825 | **0.9684** | **0.9371** | 0.992 |
| (H) Performance of shallow and DNN models built and assessed on the Sharma AFP benchmarking dataset | | | | | |
| Deep-AFPpred | **0.916** | 0.938 | 0.9268 | 0.854 | 0.969 |
| LogitBoost+RF | 0.895 | 0.94 | 0.9173 | 0.836 | 0.969 |
| LogitBoost+RF | 0.893 | 0.938 | 0.9152 | 0.831 | 0.969 |
| iAMPCN | 0.9137 | **0.9405** | **0.9271** | **0.8545** | **0.973** |
| (I) Performance of shallow and DNN models built and assessed on the Sharma AVP benchmarking dataset | | | | | |
| Deep-AVPpred | **0.913** | **0.932** | **0.9224** | **0.845** | **0.975** |
| LogitBoost+RF | 0.866 | 0.919 | 0.8924 | 0.786 | 0.962 |
| LogitBoost+RF | 0.852 | 0.928 | 0.89 | 0.782 | 0.962 |
| iAMPCN | 0.8961 | 0.9242 | 0.9101 | 0.8206 | 0.967 |

*means same algorithm with different features.

Supplementary Table S30. **Performances of different computational approaches based on StaBle-ABPpred datasets.**

| **Validation dataset** |  |  |  |  |  |  |
| --- | --- | --- | --- | --- | --- | --- |
| **Model** | **Accuracy(%)** | **Precision(%)** | **Recall(%)** | **F1-score(%)** | **MCC(%)** | **AUROC(%)** |
| StaBle-ABPpred | 97.90 | 97.85 | **97.60** | 97.60 | 94.14 | **99.92** |
| Deep-ABPpred | 97.00 | 95.76 | 96.95 | 96.22 | 92.70 | 99.43 |
| iAMPpred | 79.80 | 80.70 | 81.00 | 79.80 | 62.02 | 81.09 |
| IAMPE-KNN | 77.40 | 79.40 | 78.90 | 77.30 | 58.24 | 78.91 |
| IAMPE-SVM | 68.00 | 77.60 | 71.70 | 67.20 | 48.58 | 71.50 |
| IAMPE-RF | 78.20 | 82.20 | 80.60 | 78.10 | 62.53 | 80.48 |
| IAMPE-XGBOOST | 76.20 | 81.00 | 78.70 | 75.90 | 59.93 | 78.73 |
| iAMPCN | **97.91** | **98.40** | 96.84 | **97.61** | **95.77** | 99.74 |
| **Test dataset** |  |  |  |  |  |  |
| **Model** | **Accuracy(%)** | **Precision(%)** | **Recall(%)** | **F1-score(%)** | **MCC(%)** | **AUROC(%)** |
| StaBle-ABPpred | 97.60 | 97.60 | **97.60** | **97.60** | 94.86 | 99.58 |
| Deep-ABPpred | 96.40 | 94.92 | 96.70 | 95.94 | 92.05 | 99.10 |
| iAMPpred | 80.50 | 81.50 | 81.70 | 80.50 | 63.24 | 81.66 |
| IAMPE-KNN | 77.40 | 79.40 | 78.90 | 77.30 | 58.24 | 78.91 |
| IAMPE-SVM | 68.00 | 77.70 | 71.40 | 66.90 | 48.66 | 71.43 |
| IAMPE-RF | 78.20 | 82.10 | 80.40 | 77.80 | 62.37 | 80.32 |
| IAMPE-XGBOOST | 76.20 | 81.20 | 78.50 | 75.70 | 59.64 | 78.54 |
| iAMPCN | **97.80** | **98.09** | 96.76 | 97.42 | **95.51** | **99.73** |

Supplementary Table S31. **Performances of different computational approaches based on Pinacho-Castellanos AMPs datasets.**

| **Type of models** | **Testing datasets** | |  |  | **External datasets** | |  |  |
| --- | --- | --- | --- | --- | --- | --- | --- | --- |
|  | **SNtest** | **SPtest** | **ACCtest** | **MCCtest** | **SNext** | **SPext** | **ACCext** | **MCCext** |
| ProtDCal | 0.862 | 0.945 | 0.904 | 0.810 | 0.911 | 0.909 | 0.910 | 0.799 |
| iLearnG1 | 0.856 | 0.938 | 0.897 | 0.796 | 0.875 | 0.899 | 0.892 | 0.756 |
| iLearnG6 | 0.851 | 0.942 | 0.897 | 0.797 | 0.904 | 0.908 | 0.906 | 0.791 |
| iLearnG7 | 0.850 | 0.941 | 0.895 | 0.794 | 0.893 | 0.912 | 0.906 | 0.788 |
| iLearn Ensemble | 0.849 | 0.945 | 0.897 | 0.797 | 0.896 | 0.917 | 0.911 | 0.797 |
| iLearn ProtDCal Ensemble | 0.866 | 0.952 | 0.909 | 0.820 | 0.910 | 0.910 | 0.910 | 0.799 |
| ESM-1b | 0.840 | 0.947 | 0.894 | 0.792 | 0.904 | 0.945 | 0.932 | 0.843 |
| ESM-1b and iLearn | 0.857 | 0.954 | 0.906 | 0.815 | 0.906 | 0.936 | 0.927 | 0.833 |
| ESM-1b and ProtDCal | 0.859 | 0.962 | 0.911 | 0.826 | **0.916** | 0.929 | 0.925 | 0.830 |
| ESM-1b and iLearn and ProtDCal | **0.866** | 0.960 | 0.913 | 0.830 | 0.915 | 0.934 | 0.928 | 0.837 |
| APIN (max) | - | - | - | - | 0.915 | 0.931 | 0.926 | 0.832 |
| APIN-fusion (max) | - | - | - | - | 0.901 | **0.954** | **0.938** | **0.855** |
| Attention | 0.731 | 0.857 | 0.794 | 0.592 | 0.925 | 0.904 | 0.910 | 0.802 |
| BERT | 0.681 | 0.935 | 0.808 | 0.637 | 0.888 | 0.930 | 0.917 | 0.809 |
| LSTM | 0.795 | 0.779 | 0.787 | 0.574 | 0.932 | 0.793 | 0.836 | 0.678 |
| Consensus | 0.590 | 0.987 | 0.788 | 0.628 | 0.837 | 0.983 | 0.937 | 0.853 |
| iAMPCN | **0.866** | **0.979** | **0.922** | **0.850** | 0.890 | 0.951 | 0.932 | 0.842 |

**(**max) means selected the best prediction by multiple runs. These results were obtained from García-Jacas *et al*.’s study.

Supplementary Table S32. **AUC values of different computational approaches depending on the negative data sampling method used for training and benchmarking.**

Mean AUC (std AUC)

| Sampling method for  Training Datasets | Sampling method for  benchmark test Datasets | AMAP | AmPEP | AmPEPpy | AmpGram | Ampir | AMPScannerV2 | CS-AMPPred | Deep-AmPEP30 | iAMP-2L | iAMPCN | MACREL | MLAMP | SVM-LZ |
| --- | --- | --- | --- | --- | --- | --- | --- | --- | --- | --- | --- | --- | --- | --- |
| AMAP | AMAP | 0.9211(0.0055) | 0.7413(0.0072) | 0.9577(0.0009) | 0.974(0.0024) | 0.9802(0.0013) | 0.9781(0.0027) | 0.9016(0.0027) | 0.9373(0.0037) | 0.8322(0.0168) | 0.9815(0.0013) | 0.972(0.0011) | 0.9731(0.0023) | 0.7981(0.0091) |
| AMAP | AmpGram | 0.9016(0.0025) | 0.687(0.0107) | 0.9149(0.0012) | 0.9627(0.0009) | 0.9493(0.0023) | 0.9512(0.0085) | 0.8899(0.0021) | 0.8539(0.0093) | 0.6917(0.0971) | 0.9303(0.0049) | 0.9449(0.0009) | 0.9548(0.0016) | 0.7536(0.0116) |
| AMAP | ampir-mature | 0.871(0.005) | 0.6266(0.0111) | 0.7624(0.0025) | 0.944(0.003) | 0.9114(0.0036) | 0.7873(0.0383) | 0.8735(0.0038) | 0.698(0.0289) | 0.5165(0.0096) | 0.8579(0.0085) | 0.8257(0.0064) | 0.8475(0.0018) | 0.6728(0.0073) |
| AMAP | AMPlify | 0.9021(0.0032) | 0.6648(0.0064) | 0.9096(0.0022) | 0.9599(0.0032) | 0.9456(0.0021) | 0.9535(0.0022) | 0.8811(0.0035) | 0.8373(0.0137) | 0.7129(0.0396) | 0.9184(0.0065) | 0.9433(0.0015) | 0.9514(0.0009) | 0.7398(0.0043) |
| AMAP | AMPScannerV2 | 0.897(0.0027) | 0.6997(0.0121) | 0.9161(0.0009) | 0.9602(0.0019) | 0.9463(0.002) | 0.9465(0.01) | 0.8866(0.0014) | 0.845(0.0128) | 0.672(0.0755) | 0.9337(0.0049) | 0.9419(0.001) | 0.9508(0.0011) | 0.756(0.0038) |
| AMAP | CS-AMPPred | 0.8779(0.0052) | 0.9049(0.0043) | 0.9055(0.0025) | 0.9653(0.0021) | 0.9352(0.0024) | 0.9217(0.0161) | 0.8856(0.0045) | 0.8466(0.0332) | 0.5704(0.0166) | 0.9847(0.0012) | 0.936(0.0021) | 0.9092(0.0033) | 0.7997(0.0064) |
| AMAP | dbAMP | 0.8741(0.0063) | 0.9077(0.0055) | 0.8983(0.0017) | 0.9669(0.0017) | 0.9328(0.0042) | 0.9169(0.0262) | 0.8806(0.0036) | 0.8515(0.0372) | 0.5323(0.0044) | 0.9892(0.0013) | 0.9349(0.0029) | 0.9065(0.0039) | 0.7971(0.0076) |
| AMAP | Gabere&Noble | 0.8487(0.002) | 0.6626(0.0099) | 0.9235(0.0024) | 0.9473(0.0031) | 0.9456(0.0014) | 0.9546(0.0059) | 0.7881(0.0023) | 0.8856(0.0112) | 0.8327(0.0215) | 0.9329(0.0035) | 0.9462(0.0021) | 0.9557(0.0012) | 0.8129(0.0062) |
| AMAP | iAMP-2L | 0.8827(0.0055) | 0.8986(0.0041) | 0.8984(0.0025) | 0.9642(0.0008) | 0.9308(0.0031) | 0.9241(0.0171) | 0.8852(0.0055) | 0.8564(0.0253) | 0.5906(0.0292) | 0.9874(0.0014) | 0.9326(0.0022) | 0.9054(0.0052) | 0.7946(0.0026) |
| AMAP | Wang et al. | 0.894(0.0031) | 0.5146(0.0043) | 0.9348(0.002) | 0.9595(0.0021) | 0.96(0.0024) | 0.963(0.002) | 0.8723(0.002) | 0.8808(0.0138) | 0.8019(0.058) | 0.9489(0.0024) | 0.9547(0.0018) | 0.964(0.0019) | 0.8064(0.0081) |
| AMAP | Witten&Witten | 0.8368(0.005) | 0.723(0.0081) | 0.9144(0.0018) | 0.9445(0.001) | 0.9247(0.0048) | 0.852(0.0313) | 0.8671(0.0052) | 0.7902(0.0107) | 0.7285(0.0439) | 0.9377(0.0039) | 0.9376(0.0016) | 0.8618(0.0041) | 0.7434(0.0088) |
| AmpGram | AMAP | 0.7917(0.0105) | 0.6784(0.0117) | 0.7969(0.0036) | 0.9027(0.0064) | 0.8054(0.0034) | 0.7965(0.0208) | 0.8082(0.0108) | 0.624(0.0238) | 0.6391(0.0495) | 0.9706(0.0006) | 0.8663(0.0058) | 0.7196(0.009) | 0.8201(0.0083) |
| AmpGram | AmpGram | 0.8819(0.007) | 0.6329(0.0096) | 0.9351(0.0028) | 0.9482(0.0034) | 0.9566(0.0022) | 0.9562(0.0026) | 0.8604(0.0045) | 0.8592(0.0102) | 0.5764(0.0609) | 0.9683(0.0009) | 0.9532(0.0027) | 0.9571(0.0029) | 0.7911(0.0083) |
| AmpGram | ampir-mature | 0.8(0.005) | 0.7194(0.0057) | 0.8444(0.0044) | 0.8911(0.0033) | 0.9009(0.0014) | 0.848(0.0081) | 0.8089(0.01) | 0.7475(0.0142) | 0.5016(0.0015) | 0.8485(0.0059) | 0.8772(0.0047) | 0.8649(0.0034) | 0.732(0.0071) |
| AmpGram | AMPlify | 0.8846(0.0055) | 0.5994(0.0104) | 0.9326(0.0028) | 0.9447(0.0018) | 0.9537(0.0013) | 0.9538(0.003) | 0.849(0.0066) | 0.8513(0.011) | 0.5515(0.038) | 0.9603(0.0022) | 0.9529(0.0036) | 0.9551(0.0023) | 0.7804(0.0025) |
| AmpGram | AMPScannerV2 | 0.8769(0.0058) | 0.6504(0.0102) | 0.9327(0.0038) | 0.9454(0.0025) | 0.955(0.0026) | 0.956(0.0009) | 0.8505(0.0096) | 0.8595(0.0074) | 0.5723(0.0721) | 0.9715(0.0018) | 0.9498(0.0037) | 0.9545(0.0029) | 0.7939(0.0034) |
| AmpGram | CS-AMPPred | 0.6879(0.0099) | 0.6977(0.0057) | 0.75(0.0036) | 0.889(0.0024) | 0.7245(0.0087) | 0.7084(0.0535) | 0.7576(0.013) | 0.5567(0.0115) | 0.586(0.0204) | 0.9428(0.0027) | 0.7877(0.0046) | 0.6387(0.0077) | 0.8178(0.0072) |
| AmpGram | dbAMP | 0.6826(0.009) | 0.6901(0.0097) | 0.7472(0.0067) | 0.8811(0.0038) | 0.709(0.0051) | 0.6961(0.0471) | 0.7539(0.0139) | 0.5667(0.0179) | 0.6079(0.0199) | 0.9528(0.0030) | 0.7854(0.0065) | 0.6359(0.0088) | 0.8146(0.0061) |
| AmpGram | Gabere&Noble | 0.8445(0.0024) | 0.7146(0.0083) | 0.939(0.0036) | 0.9303(0.0031) | 0.9528(0.0024) | 0.9585(0.0041) | 0.7814(0.0034) | 0.8904(0.0054) | 0.6053(0.0223) | 0.9704(0.0006) | 0.9556(0.003) | 0.9596(0.0029) | 0.8246(0.0044) |
| AmpGram | iAMP-2L | 0.6893(0.0069) | 0.6872(0.0036) | 0.7449(0.0044) | 0.8792(0.0026) | 0.7017(0.0052) | 0.6834(0.0459) | 0.764(0.014) | 0.5557(0.0142) | 0.6198(0.02) | 0.9443(0.0038) | 0.7767(0.0072) | 0.6397(0.0133) | 0.8081(0.0048) |
| AmpGram | Wang et al. | 0.8757(0.0073) | 0.5057(0.0041) | 0.9271(0.0046) | 0.9198(0.0046) | 0.9549(0.0017) | 0.9436(0.0057) | 0.8501(0.0029) | 0.8465(0.0139) | 0.6679(0.0638) | 0.9696(0.0009) | 0.9515(0.0037) | 0.9463(0.003) | 0.8207(0.0067) |
| AmpGram | Witten&Witten | 0.8308(0.0056) | 0.6723(0.0061) | 0.9332(0.0035) | 0.9317(0.0014) | 0.9343(0.002) | 0.8705(0.0186) | 0.8325(0.0023) | 0.8295(0.007) | 0.6301(0.0428) | 0.9708(0.0012) | 0.9462(0.0031) | 0.9007(0.0021) | 0.7833(0.0029) |
| ampir-mature | AMAP | 0.718(0.0093) | 0.7489(0.0041) | 0.8156(0.0062) | 0.8176(0.0064) | 0.8183(0.0031) | 0.7968(0.0139) | 0.6951(0.0033) | 0.758(0.0143) | 0.6341(0.0283) | 0.9567(0.0015) | 0.849(0.0049) | 0.81(0.0081) | 0.7112(0.0097) |
| ampir-mature | AmpGram | 0.7278(0.0099) | 0.6541(0.0115) | 0.8266(0.0037) | 0.8058(0.0106) | 0.8082(0.0068) | 0.8195(0.0098) | 0.7204(0.0051) | 0.749(0.0121) | 0.5606(0.0583) | 0.9181(0.0036) | 0.8411(0.0028) | 0.8383(0.0051) | 0.6726(0.0103) |
| ampir-mature | ampir-mature | 0.8292(0.004) | 0.5838(0.011) | 0.9086(0.0044) | 0.9025(0.0021) | 0.9251(0.002) | 0.9279(0.0039) | 0.8078(0.0055) | 0.8682(0.009) | 0.5064(0.002) | 0.9175(0.0029) | 0.9085(0.0027) | 0.9132(0.003) | 0.7391(0.0066) |
| ampir-mature | AMPlify | 0.7395(0.0097) | 0.6359(0.0063) | 0.8204(0.0055) | 0.8112(0.0061) | 0.838(0.0064) | 0.8454(0.0088) | 0.7239(0.0127) | 0.7484(0.0138) | 0.5322(0.0196) | 0.9038(0.0034) | 0.8476(0.0028) | 0.8468(0.0039) | 0.6898(0.0033) |
| ampir-mature | AMPScannerV2 | 0.7148(0.0074) | 0.679(0.0103) | 0.823(0.0058) | 0.7933(0.0112) | 0.7966(0.0132) | 0.817(0.0079) | 0.7004(0.0107) | 0.7444(0.016) | 0.5483(0.0438) | 0.9213(0.0044) | 0.832(0.0037) | 0.8295(0.0076) | 0.6708(0.0141) |
| ampir-mature | CS-AMPPred | 0.6865(0.0091) | 0.7749(0.0052) | 0.7867(0.0113) | 0.8339(0.0045) | 0.7624(0.0105) | 0.7606(0.034) | 0.6955(0.0028) | 0.6908(0.023) | 0.5211(0.0147) | 0.9630(0.0022) | 0.8079(0.0059) | 0.753(0.0107) | 0.7426(0.0134) |
| ampir-mature | dbAMP | 0.6707(0.0082) | 0.7721(0.0058) | 0.7784(0.0075) | 0.8267(0.0067) | 0.7527(0.009) | 0.7313(0.0391) | 0.6853(0.0064) | 0.6784(0.0264) | 0.5261(0.0217) | 0.9618(0.0007) | 0.8001(0.006) | 0.7401(0.009) | 0.7229(0.005) |
| ampir-mature | Gabere&Noble | 0.7204(0.0046) | 0.5819(0.0098) | 0.8209(0.0045) | 0.8109(0.0038) | 0.8045(0.0087) | 0.8298(0.0129) | 0.7193(0.0046) | 0.7871(0.0114) | 0.6175(0.017) | 0.9206(0.0042) | 0.8335(0.004) | 0.8295(0.0059) | 0.7169(0.0066) |
| ampir-mature | iAMP-2L | 0.6859(0.0093) | 0.7693(0.0077) | 0.7773(0.0073) | 0.8302(0.0073) | 0.7522(0.0073) | 0.7556(0.0267) | 0.6989(0.0068) | 0.6861(0.0171) | 0.5584(0.026) | 0.9635(0.0010) | 0.8022(0.0043) | 0.7517(0.0117) | 0.7313(0.0066) |
| ampir-mature | Wang et al. | 0.7321(0.0083) | 0.5384(0.0105) | 0.8377(0.0035) | 0.8189(0.0096) | 0.82(0.0075) | 0.8447(0.0106) | 0.7374(0.0076) | 0.7872(0.0137) | 0.6207(0.0625) | 0.9317(0.0032) | 0.8491(0.0036) | 0.8494(0.0046) | 0.7105(0.0134) |
| ampir-mature | Witten&Witten | 0.6858(0.0104) | 0.6849(0.0074) | 0.8216(0.0062) | 0.7721(0.0141) | 0.704(0.0123) | 0.6605(0.02) | 0.6818(0.0081) | 0.6776(0.0154) | 0.5425(0.0241) | 0.9221(0.0067) | 0.828(0.0051) | 0.7569(0.0075) | 0.6534(0.0122) |
| AMPlify | AMAP | 0.7675(0.0101) | 0.6719(0.012) | 0.787(0.004) | 0.893(0.0059) | 0.7906(0.0048) | 0.7899(0.0197) | 0.7817(0.0109) | 0.6156(0.0185) | 0.6139(0.0499) | 0.9680(0.0013) | 0.8518(0.0049) | 0.7119(0.0102) | 0.8169(0.0069) |
| AMPlify | AmpGram | 0.8614(0.0091) | 0.6564(0.0092) | 0.9256(0.0018) | 0.9373(0.0045) | 0.9467(0.0041) | 0.9497(0.0015) | 0.8382(0.0088) | 0.8449(0.0086) | 0.5692(0.041) | 0.9669(0.0023) | 0.9403(0.0019) | 0.9457(0.0031) | 0.79(0.0082) |
| AMPlify | ampir-mature | 0.7844(0.0065) | 0.7261(0.0029) | 0.8325(0.0031) | 0.8795(0.0046) | 0.8846(0.004) | 0.8315(0.0107) | 0.7757(0.0085) | 0.7343(0.0165) | 0.5016(0.0012) | 0.8634(0.0051) | 0.8602(0.0037) | 0.851(0.0029) | 0.7351(0.0064) |
| AMPlify | AMPlify | 0.8723(0.0068) | 0.6202(0.008) | 0.9268(0.003) | 0.9427(0.0026) | 0.9511(0.0029) | 0.9524(0.0027) | 0.8394(0.006) | 0.8454(0.0046) | 0.5501(0.031) | 0.9648(0.0020) | 0.9451(0.0014) | 0.9497(0.0032) | 0.8155(0.0033) |
| AMPlify | AMPScannerV2 | 0.8544(0.0051) | 0.6688(0.0087) | 0.9241(0.0021) | 0.9345(0.0046) | 0.9444(0.0034) | 0.9477(0.0014) | 0.8259(0.0043) | 0.8437(0.0098) | 0.5648(0.0636) | 0.9700(0.0027) | 0.9376(0.0023) | 0.944(0.0034) | 0.791(0.0047) |
| AMPlify | CS-AMPPred | 0.6806(0.0092) | 0.6793(0.007) | 0.7405(0.0043) | 0.8851(0.0054) | 0.7027(0.0121) | 0.6929(0.0552) | 0.738(0.0123) | 0.5539(0.01) | 0.588(0.0205) | 0.9467(0.0021) | 0.7726(0.0024) | 0.6344(0.0103) | 0.8183(0.0024) |
| AMPlify | dbAMP | 0.676(0.0105) | 0.6727(0.0088) | 0.7353(0.0065) | 0.8785(0.0047) | 0.69(0.0076) | 0.6776(0.0412) | 0.7385(0.0117) | 0.5655(0.0177) | 0.6031(0.0188) | 0.9550(0.0020) | 0.7688(0.0072) | 0.6323(0.0049) | 0.8159(0.0056) |
| AMPlify | Gabere&Noble | 0.8385(0.004) | 0.6794(0.008) | 0.9311(0.0027) | 0.9236(0.0036) | 0.9462(0.0024) | 0.9513(0.0038) | 0.7777(0.0038) | 0.8712(0.0037) | 0.5921(0.0214) | 0.9682(0.0011) | 0.9443(0.0027) | 0.9487(0.0031) | 0.8175(0.0037) |
| AMPlify | iAMP-2L | 0.6816(0.0099) | 0.6682(0.0061) | 0.7307(0.0039) | 0.8759(0.0043) | 0.6811(0.0041) | 0.6711(0.0461) | 0.7424(0.0099) | 0.5533(0.011) | 0.6062(0.0204) | 0.9487(0.0014) | 0.7648(0.0049) | 0.6324(0.0096) | 0.8077(0.0054) |
| AMPlify | Wang et al. | 0.8629(0.0042) | 0.5305(0.0026) | 0.9185(0.003) | 0.9126(0.002) | 0.9462(0.0027) | 0.9377(0.0039) | 0.8368(0.0054) | 0.8284(0.012) | 0.6584(0.0561) | 0.9678(0.0016) | 0.9399(0.0021) | 0.9339(0.0025) | 0.8184(0.0041) |
| AMPlify | Witten&Witten | 0.8132(0.0073) | 0.6943(0.0098) | 0.9249(0.002) | 0.9203(0.0033) | 0.9193(0.0051) | 0.8617(0.026) | 0.8075(0.0087) | 0.8087(0.0078) | 0.6139(0.0507) | 0.9673(0.0013) | 0.9349(0.0018) | 0.8957(0.0054) | 0.778(0.0041) |
| AMPScannerV2 | AMAP | 0.8001(0.0101) | 0.6805(0.0119) | 0.802(0.0046) | 0.9085(0.0051) | 0.805(0.0064) | 0.803(0.0166) | 0.8214(0.0077) | 0.6259(0.0196) | 0.6464(0.0491) | 0.9694(0.0012) | 0.8742(0.0017) | 0.727(0.0108) | 0.8205(0.0067) |
| AMPScannerV2 | AmpGram | 0.8888(0.0045) | 0.613(0.0053) | 0.9413(0.0016) | 0.9534(0.0022) | 0.9608(0.0019) | 0.9598(0.002) | 0.8689(0.0035) | 0.864(0.0053) | 0.5744(0.0557) | 0.9674(0.0029) | 0.9588(0.0008) | 0.9628(0.0013) | 0.7967(0.0068) |
| AMPScannerV2 | ampir-mature | 0.8116(0.0077) | 0.7136(0.0076) | 0.8494(0.0044) | 0.892(0.0021) | 0.9042(0.0042) | 0.8502(0.0066) | 0.8144(0.0044) | 0.7463(0.0172) | 0.5014(0.0011) | 0.8370(0.0097) | 0.8825(0.0033) | 0.869(0.0032) | 0.7299(0.005) |
| AMPScannerV2 | AMPlify | 0.8894(0.007) | 0.5779(0.0087) | 0.94(0.0035) | 0.9497(0.0022) | 0.9581(0.0025) | 0.9569(0.0047) | 0.8585(0.0047) | 0.8584(0.0079) | 0.5483(0.0403) | 0.9598(0.0027) | 0.9577(0.0013) | 0.9611(0.0017) | 0.7852(0.0027) |
| AMPScannerV2 | AMPScannerV2 | 0.8887(0.0079) | 0.6275(0.0104) | 0.9419(0.0021) | 0.9531(0.0015) | 0.9619(0.0017) | 0.9622(0.0027) | 0.8628(0.0034) | 0.8645(0.0074) | 0.569(0.0743) | 0.9726(0.0020) | 0.9585(0.001) | 0.963(0.0019) | 0.8085(0.0071) |
| AMPScannerV2 | CS-AMPPred | 0.6941(0.0059) | 0.7028(0.0117) | 0.7523(0.008) | 0.8923(0.0034) | 0.723(0.01) | 0.7114(0.051) | 0.7641(0.0041) | 0.5548(0.0063) | 0.5907(0.0246) | 0.9359(0.0031) | 0.791(0.0042) | 0.6437(0.009) | 0.8184(0.0047) |
| AMPScannerV2 | dbAMP | 0.6925(0.0074) | 0.6918(0.0128) | 0.7521(0.0065) | 0.8856(0.0042) | 0.7062(0.0059) | 0.6913(0.0506) | 0.7645(0.0076) | 0.5675(0.0203) | 0.6142(0.015) | 0.9502(0.0016) | 0.7886(0.0097) | 0.642(0.0041) | 0.8145(0.0054) |
| AMPScannerV2 | Gabere&Noble | 0.8462(0.0026) | 0.7389(0.0089) | 0.9454(0.002) | 0.9338(0.005) | 0.9562(0.0018) | 0.9619(0.0038) | 0.7831(0.0041) | 0.8938(0.0032) | 0.6109(0.0309) | 0.9708(0.0010) | 0.9605(0.0009) | 0.9652(0.0016) | 0.8304(0.0049) |
| AMPScannerV2 | iAMP-2L | 0.6952(0.0052) | 0.6932(0.0094) | 0.7463(0.005) | 0.8823(0.0034) | 0.6988(0.0036) | 0.6874(0.0417) | 0.7672(0.0049) | 0.5548(0.0091) | 0.6186(0.0191) | 0.9379(0.0029) | 0.7834(0.0038) | 0.6427(0.0093) | 0.8085(0.0034) |
| AMPScannerV2 | Wang et al. | 0.8827(0.0027) | 0.5167(0.0072) | 0.9346(0.0021) | 0.9228(0.0034) | 0.958(0.0014) | 0.9468(0.0041) | 0.8551(0.0034) | 0.8474(0.0151) | 0.6745(0.0574) | 0.9690(0.0013) | 0.957(0.0019) | 0.9508(0.0022) | 0.8235(0.006) |
| AMPScannerV2 | Witten&Witten | 0.8429(0.0068) | 0.6538(0.01) | 0.9416(0.0009) | 0.9382(0.0019) | 0.941(0.0036) | 0.8803(0.0249) | 0.8468(0.0044) | 0.8301(0.007) | 0.6352(0.0453) | 0.9709(0.0019) | 0.9543(0.0019) | 0.9086(0.0033) | 0.7852(0.0032) |
| CS-AMPPred | AMAP | 0.9264(0.0065) | 0.81(0.0091) | 0.9686(0.0026) | 0.9735(0.0019) | 0.9751(0.0021) | 0.9736(0.0047) | 0.8949(0.0055) | 0.9534(0.0093) | 0.9415(0.0085) | 0.9712(0.0010) | 0.9766(0.0015) | 0.9834(0.0017) | 0.7626(0.0053) |
| CS-AMPPred | AmpGram | 0.8713(0.0078) | 0.8348(0.0116) | 0.8381(0.0092) | 0.94(0.0044) | 0.8971(0.0041) | 0.8901(0.0281) | 0.8656(0.0029) | 0.8071(0.0103) | 0.9012(0.0234) | 0.8818(0.0050) | 0.8974(0.0033) | 0.9193(0.0045) | 0.711(0.0059) |
| CS-AMPPred | ampir-mature | 0.9(0.0047) | 0.5831(0.0046) | 0.5984(0.0101) | 0.9392(0.0063) | 0.7286(0.0039) | 0.6532(0.0895) | 0.8667(0.0046) | 0.724(0.0535) | 0.6436(0.0736) | 0.8694(0.0061) | 0.6161(0.0144) | 0.603(0.011) | 0.6664(0.0104) |
| CS-AMPPred | AMPlify | 0.8821(0.0079) | 0.8244(0.008) | 0.8317(0.0027) | 0.9404(0.0047) | 0.8911(0.0058) | 0.9181(0.0138) | 0.8642(0.0034) | 0.7852(0.0341) | 0.8924(0.0408) | 0.8726(0.0054) | 0.8957(0.0045) | 0.9225(0.0053) | 0.7173(0.0066) |
| CS-AMPPred | AMPScannerV2 | 0.8602(0.009) | 0.8408(0.01) | 0.8369(0.0091) | 0.9364(0.0032) | 0.8926(0.002) | 0.8842(0.0183) | 0.8581(0.0026) | 0.7944(0.038) | 0.8573(0.0366) | 0.8830(0.0059) | 0.8915(0.0029) | 0.9112(0.0036) | 0.713(0.0061) |
| CS-AMPPred | CS-AMPPred | 0.9472(0.0044) | 0.5735(0.0096) | 0.995(0.0005) | 0.9833(0.0021) | 0.9913(0.0006) | 0.9932(0.0011) | 0.9073(0.0039) | 0.9888(0.0023) | 0.7446(0.0384) | 0.9932(0.0009) | 0.9904(0.0005) | 0.9931(0.0007) | 0.7884(0.008) |
| CS-AMPPred | dbAMP | 0.9474(0.0036) | 0.5807(0.007) | 0.9959(0.0004) | 0.9825(0.0022) | 0.9916(0.0009) | 0.9945(0.0007) | 0.9013(0.0031) | 0.9882(0.0012) | 0.5213(0.008) | 0.9965(0.0007) | 0.9924(0.0006) | 0.994(0.0004) | 0.8077(0.0065) |
| CS-AMPPred | Gabere&Noble | 0.8367(0.0029) | 0.5801(0.0154) | 0.8465(0.0046) | 0.9273(0.0037) | 0.88(0.0017) | 0.9098(0.0178) | 0.7864(0.0032) | 0.8568(0.0097) | 0.9458(0.0071) | 0.8832(0.0048) | 0.8926(0.0015) | 0.9118(0.0039) | 0.7902(0.0053) |
| CS-AMPPred | iAMP-2L | 0.9527(0.0032) | 0.5233(0.0062) | 0.9969(0.0003) | 0.9824(0.0016) | 0.9922(0.0004) | 0.9948(0.001) | 0.9111(0.0041) | 0.9901(0.001) | 0.5773(0.0427) | 0.9966(0.0005) | 0.994(0.0006) | 0.9954(0.0002) | 0.8234(0.0023) |
| CS-AMPPred | Wang et al. | 0.8782(0.0035) | 0.7258(0.0162) | 0.859(0.014) | 0.9417(0.0038) | 0.9056(0.005) | 0.9347(0.0083) | 0.8639(0.0052) | 0.85(0.0192) | 0.9135(0.0351) | 0.9114(0.0046) | 0.9101(0.0027) | 0.9302(0.0062) | 0.7713(0.0086) |
| CS-AMPPred | Witten&Witten | 0.7551(0.0045) | 0.8414(0.0025) | 0.8458(0.0058) | 0.9162(0.0036) | 0.8914(0.003) | 0.7437(0.0401) | 0.8139(0.0048) | 0.6718(0.0197) | 0.8853(0.0246) | 0.8904(0.0088) | 0.8932(0.0032) | 0.7784(0.008) | 0.6988(0.0035) |
| dbAMP | AMAP | 0.9359(0.0043) | 0.8123(0.0078) | 0.9724(0.0033) | 0.9768(0.0026) | 0.9793(0.0013) | 0.9776(0.0045) | 0.9083(0.0053) | 0.9567(0.0067) | 0.9538(0.0075) | 0.9708(0.0016) | 0.9773(0.0012) | 0.9863(0.0013) | 0.7722(0.0086) |
| dbAMP | AmpGram | 0.8874(0.0023) | 0.8313(0.0102) | 0.8382(0.0096) | 0.9459(0.0032) | 0.8952(0.0032) | 0.902(0.0289) | 0.8805(0.0058) | 0.8119(0.018) | 0.924(0.0166) | 0.8847(0.0034) | 0.8978(0.0047) | 0.9255(0.0024) | 0.7219(0.0039) |
| dbAMP | ampir-mature | 0.8968(0.0039) | 0.5336(0.0059) | 0.645(0.0082) | 0.9336(0.0053) | 0.7261(0.0088) | 0.6764(0.0868) | 0.8632(0.0068) | 0.7761(0.0513) | 0.6931(0.0905) | 0.8498(0.0084) | 0.5903(0.0152) | 0.5612(0.0094) | 0.661(0.005) |
| dbAMP | AMPlify | 0.8967(0.0046) | 0.8245(0.0145) | 0.831(0.0014) | 0.9472(0.0036) | 0.8863(0.0043) | 0.9235(0.0113) | 0.8806(0.0031) | 0.802(0.0373) | 0.9177(0.0287) | 0.8761(0.0051) | 0.8967(0.0037) | 0.9293(0.0019) | 0.7263(0.0052) |
| dbAMP | AMPScannerV2 | 0.8817(0.0046) | 0.8384(0.012) | 0.8394(0.0102) | 0.9452(0.0026) | 0.8925(0.0039) | 0.9019(0.0194) | 0.8797(0.0046) | 0.8062(0.0414) | 0.8876(0.0307) | 0.8880(0.0057) | 0.8973(0.0028) | 0.9221(0.0017) | 0.7259(0.0091) |
| dbAMP | CS-AMPPred | 0.9536(0.0023) | 0.6551(0.0091) | 0.9859(0.0005) | 0.9833(0.0017) | 0.9908(0.0004) | 0.995(0.0014) | 0.9173(0.0026) | 0.992(0.0014) | 0.6999(0.0518) | 0.9933(0.0007) | 0.9788(0.002) | 0.9932(0.0005) | 0.8275(0.0097) |
| dbAMP | dbAMP | 0.9527(0.0033) | 0.5692(0.007) | 0.9964(0.0002) | 0.9845(0.0022) | 0.9925(0.0005) | 0.9956(0.0007) | 0.9125(0.0022) | 0.9886(0.0027) | 0.5599(0.0317) | 0.9942(0.0007) | 0.9912(0.0005) | 0.9939(0.0005) | 0.7856(0.0048) |
| dbAMP | Gabere&Noble | 0.8446(0.001) | 0.5732(0.0158) | 0.8568(0.0037) | 0.9351(0.0068) | 0.8817(0.0019) | 0.9187(0.0122) | 0.7881(0.0038) | 0.8675(0.0188) | 0.9576(0.0066) | 0.8884(0.0050) | 0.8948(0.002) | 0.9211(0.0024) | 0.7973(0.0057) |
| dbAMP | iAMP-2L | 0.957(0.0023) | 0.5054(0.0044) | 0.9981(0.0001) | 0.9832(0.0015) | 0.9923(0.0004) | 0.9955(0.0012) | 0.9189(0.0042) | 0.992(0.0009) | 0.6269(0.0488) | 0.9953(0.0006) | 0.9951(0.0004) | 0.9964(0.0003) | 0.824(0.0046) |
| dbAMP | Wang et al. | 0.8889(0.003) | 0.7244(0.0179) | 0.8636(0.014) | 0.9455(0.0024) | 0.9068(0.0037) | 0.9419(0.0061) | 0.8701(0.0026) | 0.8574(0.0275) | 0.9357(0.0268) | 0.9144(0.0040) | 0.9111(0.0038) | 0.9371(0.0044) | 0.7757(0.0035) |
| dbAMP | Witten&Witten | 0.7957(0.006) | 0.8327(0.0037) | 0.8489(0.0063) | 0.9267(0.0034) | 0.898(0.0017) | 0.7855(0.0421) | 0.848(0.0042) | 0.6949(0.0222) | 0.9153(0.0192) | 0.9023(0.0015) | 0.9009(0.0031) | 0.7935(0.0046) | 0.7145(0.0037) |
| Gabere&Noble | AMAP | 0.8009(0.0042) | 0.6812(0.0095) | 0.8021(0.0019) | 0.9056(0.0042) | 0.8059(0.0048) | 0.7983(0.0203) | 0.8202(0.004) | 0.6254(0.0196) | 0.6408(0.0531) | 0.9714(0.0013) | 0.8726(0.0022) | 0.7252(0.0103) | 0.8207(0.0046) |
| Gabere&Noble | AmpGram | 0.8882(0.0029) | 0.6173(0.0044) | 0.9398(0.0009) | 0.9521(0.0022) | 0.9602(0.0007) | 0.9592(0.002) | 0.8701(0.0027) | 0.8613(0.0049) | 0.5732(0.0513) | 0.9720(0.0011) | 0.9581(0.0003) | 0.9619(0.0007) | 0.7975(0.0054) |
| Gabere&Noble | ampir-mature | 0.8115(0.0033) | 0.7118(0.0017) | 0.8479(0.0016) | 0.8929(0.0015) | 0.9042(0.0015) | 0.8505(0.0068) | 0.8169(0.0032) | 0.7491(0.0172) | 0.5013(0.0008) | 0.8453(0.0045) | 0.882(0.0015) | 0.8696(0.0026) | 0.7321(0.0044) |
| Gabere&Noble | AMPlify | 0.889(0.003) | 0.5822(0.0045) | 0.9384(0.0017) | 0.948(0.0013) | 0.9572(0.0005) | 0.9564(0.0028) | 0.8594(0.0029) | 0.8559(0.0077) | 0.5514(0.037) | 0.9652(0.0015) | 0.9568(0.0006) | 0.96(0.0011) | 0.7856(0.0013) |
| Gabere&Noble | AMPScannerV2 | 0.8862(0.0023) | 0.6322(0.0079) | 0.9392(0.0008) | 0.9506(0.0022) | 0.9601(0.0007) | 0.9595(0.0014) | 0.8638(0.002) | 0.861(0.0072) | 0.5714(0.0769) | 0.9754(0.0014) | 0.9564(0.0008) | 0.9606(0.001) | 0.8056(0.0054) |
| Gabere&Noble | CS-AMPPred | 0.6933(0.0014) | 0.7028(0.0075) | 0.7524(0.0035) | 0.8905(0.0029) | 0.7238(0.0075) | 0.709(0.0522) | 0.7649(0.0068) | 0.5553(0.0088) | 0.5917(0.021) | 0.9385(0.0040) | 0.7899(0.0025) | 0.6429(0.0091) | 0.8181(0.0021) |
| Gabere&Noble | dbAMP | 0.6896(0.003) | 0.695(0.0088) | 0.7509(0.003) | 0.8833(0.0045) | 0.7068(0.0058) | 0.6935(0.0476) | 0.7659(0.0041) | 0.5665(0.0179) | 0.6167(0.0195) | 0.9505(0.0025) | 0.7875(0.0069) | 0.6392(0.0047) | 0.8172(0.0026) |
| Gabere&Noble | Gabere&Noble | 0.8467(0.0018) | 0.7354(0.0066) | 0.9443(0.0021) | 0.9323(0.0034) | 0.9558(0.0006) | 0.9605(0.0034) | 0.7827(0.0031) | 0.8909(0.0013) | 0.614(0.0233) | 0.9745(0.0006) | 0.9599(0.0014) | 0.9645(0.001) | 0.83(0.0045) |
| Gabere&Noble | iAMP-2L | 0.6936(0.0011) | 0.6936(0.0081) | 0.7471(0.0018) | 0.8809(0.0028) | 0.6996(0.005) | 0.6858(0.0432) | 0.7694(0.004) | 0.5554(0.0115) | 0.6265(0.0179) | 0.9407(0.0023) | 0.7811(0.0045) | 0.6413(0.0113) | 0.8079(0.0021) |
| Gabere&Noble | Wang et al. | 0.881(0.0029) | 0.5147(0.0025) | 0.933(0.0013) | 0.9217(0.003) | 0.9573(0.0005) | 0.9462(0.0052) | 0.8551(0.0018) | 0.8464(0.015) | 0.6734(0.0624) | 0.9728(0.0007) | 0.9558(0.0014) | 0.9497(0.0013) | 0.8255(0.002) |
| Gabere&Noble | Witten&Witten | 0.8416(0.005) | 0.6555(0.0054) | 0.9392(0.0008) | 0.9364(0.0017) | 0.9399(0.0019) | 0.8778(0.0217) | 0.8475(0.0033) | 0.8294(0.0037) | 0.6323(0.0458) | 0.9741(0.0008) | 0.9523(0.0012) | 0.9073(0.0032) | 0.7872(0.001) |
| iAMP-2L | AMAP | 0.9319(0.0029) | 0.8121(0.0066) | 0.9686(0.0035) | 0.9753(0.0016) | 0.9755(0.0021) | 0.9736(0.0033) | 0.8978(0.0053) | 0.9548(0.009) | 0.9542(0.0122) | 0.9708(0.0008) | 0.9773(0.0011) | 0.9839(0.001) | 0.7727(0.0066) |
| iAMP-2L | AmpGram | 0.8768(0.0016) | 0.8381(0.0092) | 0.8352(0.0096) | 0.941(0.0031) | 0.8911(0.0025) | 0.8892(0.0301) | 0.8694(0.0046) | 0.8164(0.0133) | 0.9276(0.0176) | 0.8721(0.0075) | 0.8965(0.0045) | 0.9195(0.0022) | 0.7132(0.0114) |
| iAMP-2L | ampir-mature | 0.9015(0.0033) | 0.5303(0.0069) | 0.6366(0.011) | 0.9378(0.0055) | 0.729(0.0056) | 0.6757(0.0937) | 0.8716(0.0025) | 0.7787(0.0461) | 0.6838(0.0823) | 0.8656(0.0039) | 0.5893(0.0133) | 0.5698(0.012) | 0.6637(0.005) |
| iAMP-2L | AMPlify | 0.8867(0.0024) | 0.8273(0.0098) | 0.8271(0.0017) | 0.9424(0.0039) | 0.8819(0.004) | 0.9197(0.0125) | 0.868(0.0037) | 0.7992(0.032) | 0.9209(0.0323) | 0.8631(0.0105) | 0.8929(0.0032) | 0.923(0.0021) | 0.7251(0.0067) |
| iAMP-2L | AMPScannerV2 | 0.8648(0.0038) | 0.8423(0.0116) | 0.8345(0.0104) | 0.9381(0.004) | 0.8874(0.0029) | 0.8863(0.0194) | 0.8602(0.0062) | 0.8049(0.0411) | 0.8956(0.0258) | 0.8735(0.0073) | 0.8926(0.0024) | 0.9127(0.0014) | 0.7134(0.0076) |
| iAMP-2L | CS-AMPPred | 0.9538(0.0024) | 0.6465(0.0061) | 0.9862(0.0009) | 0.9835(0.0018) | 0.9907(0.0002) | 0.9947(0.0014) | 0.9155(0.0024) | 0.992(0.0008) | 0.7096(0.051) | 0.9966(0.0002) | 0.9804(0.0012) | 0.9935(0.0001) | 0.8256(0.0079) |
| iAMP-2L | dbAMP | 0.9525(0.0017) | 0.5489(0.0125) | 0.9975(0.0001) | 0.9838(0.0019) | 0.9922(0.0003) | 0.9956(0.0006) | 0.9053(0.0027) | 0.99(0.0019) | 0.5506(0.0311) | 0.9976(0.0002) | 0.9941(0.0004) | 0.9954(0.0003) | 0.8117(0.0034) |
| iAMP-2L | Gabere&Noble | 0.8375(0.0018) | 0.5847(0.0153) | 0.8472(0.0047) | 0.9301(0.0078) | 0.8738(0.0015) | 0.9095(0.0138) | 0.7877(0.0036) | 0.8674(0.0199) | 0.9588(0.0049) | 0.8735(0.0065) | 0.8912(0.0011) | 0.9122(0.0029) | 0.7929(0.0061) |
| iAMP-2L | iAMP-2L | 0.951(0.0016) | 0.5371(0.0113) | 0.9964(0.0004) | 0.9832(0.0012) | 0.9921(0.0003) | 0.9946(0.0012) | 0.9135(0.0032) | 0.9898(0.0015) | 0.6172(0.0437) | 0.9958(0.0003) | 0.9916(0.0009) | 0.9945(0.0003) | 0.784(0.0043) |
| iAMP-2L | Wang et al. | 0.8805(0.0028) | 0.7302(0.0178) | 0.8574(0.0139) | 0.942(0.0021) | 0.8999(0.0042) | 0.9349(0.0092) | 0.8665(0.0024) | 0.8588(0.0266) | 0.9374(0.0256) | 0.9056(0.0041) | 0.9084(0.0034) | 0.9304(0.0037) | 0.7718(0.006) |
| iAMP-2L | Witten&Witten | 0.768(0.0024) | 0.841(0.0028) | 0.8426(0.0063) | 0.9179(0.0031) | 0.8921(0.0023) | 0.7528(0.0389) | 0.8218(0.0065) | 0.6801(0.0108) | 0.9152(0.0178) | 0.8805(0.0088) | 0.8926(0.0029) | 0.781(0.0043) | 0.7029(0.0042) |
| Wang et al. | AMAP | 0.835(0.0032) | 0.7157(0.0076) | 0.8246(0.005) | 0.9226(0.0029) | 0.8558(0.0042) | 0.8159(0.0134) | 0.8397(0.0023) | 0.6812(0.0273) | 0.6685(0.042) | 0.9772(0.0017) | 0.8933(0.0021) | 0.7729(0.0127) | 0.8118(0.0089) |
| Wang et al. | AmpGram | 0.8892(0.0018) | 0.6263(0.0043) | 0.936(0.0018) | 0.9541(0.0011) | 0.9579(0.0005) | 0.958(0.0011) | 0.8714(0.0014) | 0.8593(0.005) | 0.58(0.056) | 0.9682(0.0025) | 0.9548(0.001) | 0.959(0.0009) | 0.7846(0.0062) |
| Wang et al. | ampir-mature | 0.8229(0.0034) | 0.7189(0.0021) | 0.8534(0.0026) | 0.9225(0.0017) | 0.9202(0.0022) | 0.8657(0.0061) | 0.828(0.0038) | 0.7699(0.0156) | 0.5016(0.0009) | 0.8549(0.0058) | 0.8881(0.0027) | 0.8878(0.003) | 0.7174(0.0025) |
| Wang et al. | AMPlify | 0.8893(0.0016) | 0.594(0.0066) | 0.9342(0.0021) | 0.9506(0.0023) | 0.9553(0.0008) | 0.9558(0.0019) | 0.8615(0.004) | 0.8524(0.0077) | 0.5552(0.0377) | 0.9612(0.0033) | 0.9543(0.001) | 0.9567(0.0005) | 0.7739(0.0017) |
| Wang et al. | AMPScannerV2 | 0.8851(0.0036) | 0.6424(0.0078) | 0.9342(0.0011) | 0.9509(0.0007) | 0.9562(0.0009) | 0.9557(0.0031) | 0.8634(0.0036) | 0.8581(0.0083) | 0.574(0.0703) | 0.9717(0.0018) | 0.9518(0.0011) | 0.9562(0.0005) | 0.7873(0.0049) |
| Wang et al. | CS-AMPPred | 0.7262(0.0054) | 0.7396(0.0075) | 0.7679(0.0042) | 0.9063(0.0035) | 0.753(0.0112) | 0.7101(0.0591) | 0.7916(0.0058) | 0.5805(0.01) | 0.5725(0.0206) | 0.9526(0.0052) | 0.8106(0.0046) | 0.6749(0.0102) | 0.8161(0.0043) |
| Wang et al. | dbAMP | 0.7203(0.0033) | 0.7329(0.0102) | 0.7629(0.0039) | 0.8997(0.0044) | 0.7374(0.0055) | 0.6961(0.0483) | 0.79(0.0033) | 0.5935(0.0222) | 0.5997(0.0169) | 0.9626(0.0037) | 0.8101(0.0064) | 0.6702(0.0073) | 0.8123(0.0019) |
| Wang et al. | Gabere&Noble | 0.8461(0.0014) | 0.7155(0.0064) | 0.9402(0.0021) | 0.9368(0.0039) | 0.9538(0.0011) | 0.9599(0.0034) | 0.784(0.0029) | 0.8874(0.0026) | 0.6462(0.0239) | 0.9707(0.0013) | 0.9567(0.0013) | 0.9608(0.0005) | 0.8205(0.0035) |
| Wang et al. | iAMP-2L | 0.7282(0.0033) | 0.7311(0.0097) | 0.7603(0.0019) | 0.8976(0.0026) | 0.7325(0.0035) | 0.6939(0.0455) | 0.7968(0.0042) | 0.5821(0.0157) | 0.6189(0.019) | 0.9547(0.0033) | 0.8038(0.0045) | 0.6755(0.0105) | 0.8062(0.0032) |
| Wang et al. | Wang et al. | 0.8838(0.0022) | 0.5372(0.0059) | 0.9426(0.0008) | 0.9459(0.0021) | 0.9638(0.001) | 0.9602(0.003) | 0.8589(0.0033) | 0.8805(0.0094) | 0.6845(0.0668) | 0.9733(0.0010) | 0.959(0.0013) | 0.9637(0.0013) | 0.817(0.0024) |
| Wang et al. | Witten&Witten | 0.8361(0.0044) | 0.6639(0.0066) | 0.9347(0.0008) | 0.9376(0.0018) | 0.9357(0.0017) | 0.8711(0.0223) | 0.8461(0.0028) | 0.821(0.0054) | 0.6336(0.0495) | 0.9713(0.0010) | 0.9483(0.001) | 0.8979(0.0023) | 0.774(0.002) |
| Witten&Witten | AMAP | 0.8043(0.0115) | 0.695(0.0059) | 0.8067(0.0051) | 0.9126(0.0055) | 0.8229(0.0043) | 0.8055(0.0141) | 0.8307(0.0101) | 0.6281(0.0264) | 0.6412(0.0531) | 0.9191(0.0051) | 0.8747(0.0049) | 0.7336(0.0065) | 0.8238(0.0065) |
| Witten&Witten | AmpGram | 0.8975(0.0038) | 0.6241(0.0089) | 0.9399(0.002) | 0.9605(0.0015) | 0.9607(0.0013) | 0.9591(0.0031) | 0.8816(0.0041) | 0.8716(0.0038) | 0.5805(0.0642) | 0.9210(0.0057) | 0.9592(0.0021) | 0.962(0.0016) | 0.8005(0.0125) |
| Witten&Witten | ampir-mature | 0.8044(0.0037) | 0.7188(0.0035) | 0.8496(0.005) | 0.8954(0.0054) | 0.9075(0.0039) | 0.8544(0.006) | 0.818(0.0037) | 0.7504(0.0164) | 0.5029(0.0016) | 0.7405(0.0160) | 0.8841(0.0045) | 0.8674(0.0049) | 0.7447(0.0074) |
| Witten&Witten | AMPlify | 0.8958(0.0012) | 0.5939(0.0079) | 0.9377(0.0029) | 0.9549(0.0044) | 0.9541(0.001) | 0.9542(0.0048) | 0.8687(0.0062) | 0.8641(0.0093) | 0.552(0.0407) | 0.9103(0.0080) | 0.9571(0.0026) | 0.9571(0.0015) | 0.7952(0.0037) |
| Witten&Witten | AMPScannerV2 | 0.8957(0.003) | 0.6409(0.008) | 0.9395(0.0036) | 0.9587(0.0035) | 0.9602(0.002) | 0.9587(0.0042) | 0.8764(0.0052) | 0.8732(0.0028) | 0.5535(0.0601) | 0.9286(0.0046) | 0.9577(0.0023) | 0.9603(0.0019) | 0.806(0.0083) |
| Witten&Witten | CS-AMPPred | 0.684(0.0099) | 0.7107(0.0046) | 0.762(0.006) | 0.8946(0.0022) | 0.7457(0.0146) | 0.7096(0.058) | 0.7532(0.0082) | 0.5545(0.01) | 0.572(0.0268) | 0.8531(0.0167) | 0.7967(0.0079) | 0.6516(0.0056) | 0.8256(0.0033) |
| Witten&Witten | dbAMP | 0.6842(0.0083) | 0.7016(0.0106) | 0.7588(0.0087) | 0.8882(0.0048) | 0.7223(0.0096) | 0.6952(0.0512) | 0.7622(0.0089) | 0.5671(0.0179) | 0.6198(0.0215) | 0.8759(0.0144) | 0.7941(0.0105) | 0.6445(0.0055) | 0.8226(0.0023) |
| Witten&Witten | Gabere&Noble | 0.8481(0.0017) | 0.7348(0.0069) | 0.9417(0.0029) | 0.9348(0.0035) | 0.9512(0.0012) | 0.9589(0.0027) | 0.7847(0.0027) | 0.9022(0.0042) | 0.6154(0.021) | 0.9278(0.0052) | 0.9596(0.002) | 0.9637(0.0012) | 0.8237(0.0059) |
| Witten&Witten | iAMP-2L | 0.6843(0.0078) | 0.7027(0.0099) | 0.7558(0.0089) | 0.8855(0.0033) | 0.7188(0.01) | 0.6865(0.0379) | 0.7591(0.0089) | 0.5533(0.01) | 0.6313(0.0196) | 0.8571(0.0189) | 0.7884(0.0046) | 0.6478(0.0141) | 0.8115(0.0062) |
| Witten&Witten | Wang et al. | 0.8852(0.0045) | 0.5072(0.0056) | 0.9309(0.0029) | 0.9256(0.0046) | 0.9575(0.0021) | 0.9453(0.0055) | 0.8607(0.0024) | 0.8577(0.0167) | 0.671(0.0616) | 0.9235(0.0046) | 0.956(0.0028) | 0.9512(0.0018) | 0.8275(0.0047) |
| Witten&Witten | Witten&Witten | 0.9106(0.0037) | 0.6273(0.0061) | 0.944(0.0037) | 0.9617(0.003) | 0.9673(0.001) | 0.9661(0.0058) | 0.8885(0.0038) | 0.8951(0.0045) | 0.6314(0.0457) | 0.9692(0.0082) | 0.9622(0.0022) | 0.9694(0.0023) | 0.8109(0.0038) |

Supplementary Table S33. **Performance comparison of different kinds of hidden dense layers in terms of AUC on the AMP dataset, antibacterial dataset, antifungal dataset, antiviral dataset, and antiparasitic dataset based on 10-fold stratified cross-validation test.**

| **Prediction target** | **Hidden layers (mean AUCs and std AUCs)** | | | | | | |
| --- | --- | --- | --- | --- | --- | --- | --- |
|  | **[32]** | **[64]** | **[128]** | **[128, 64]** | **[64, 32]** | **[128, 64, 32]** | **None** |
| AMP | 0.9476(0.0091) | 0.9469(0.0085) | 0.9459(0.0074) | 0.9446(0.0077) | 0.9435(0.0087) | 0.9450(0.0065) | \| 0.9541(0.007) \| \| --- \| |
| antibacterial | 0.8942(0.0069) | 0.8952(0.0071) | 0.8925(0.0086) | 0.8898(0.0073) | 0.8895(0.0063) | 0.8895(0.0102) | 0.9029(0.0069) |
| antifungal | 0.8650(0.0094) | 0.8581(0.0104) | 0.8633(0.0079) | 0.8604(0.0098) | 0.8587(0.0124) | 0.8557(0.0122) | 0.8713(0.0089) |
| antiviral | 0.8316(0.0151) | 0.8331(0.0127) | 0.8290(0.0119) | 0.8280(0.0113) | 0.8287(0.0101) | 0.8239(0.0102) | 0.8400(0.0105) |
| antiparasitic | 0.8078(0.0246) | 0.8015(0.0304) | 0.8030(0.0298) | 0.8122(0.0272) | 0.8084(0.0304) | 0.8081(0.0313) | 0.8071(0.0350) |

Supplementary Table S34. **Performance comparison with RF and XGBoost models (features extracted by the ESM-2 model) for predicting AMPs based on the AMP independent test dataset.** The sequence identity cut-off threshold of the AMP training dataset was 40%.

| **Method** | **Precision** | **Sensitivity** | **Specificity** | **Accuracy** | **F1** | **MCC** | **AUC** |
| --- | --- | --- | --- | --- | --- | --- | --- |
| RF | **0.9922** | 0.8775 | **0.9973** | 0.9374 | 0.9313 | 0.9097 | 0.9966 |
| XGBoost | 0.9876 | 0.9668 | 0.9952 | 0.9810 | 0.9771 | 0.9683 | **0.9987** |
| iAMPCN | 0.9809 | **1.0000** | 0.9923 | **0.9962** | **0.9904** | **0.9866** | 0.9973 |

Supplementary Table S35. **Performance comparison with RF and XGBoost models (features extracted by the ESM-2 model) for predicting AMP functional activities based on balanced independent test datasets.**

| **Activity** | **Method** | **Precision** | **Sensitivity** | **Specificity** | **Accuracy** | **F1** | **MCC** | **AUC** |
| --- | --- | --- | --- | --- | --- | --- | --- | --- |
| Antibacterial | RF | 0.6621 | 0.7636 | 0.6094 | 0.6865 | 0.7092 | 0.3775 | 0.7525 |
|  | XGBoost | **0.6736** | 0.7700 | **0.6260** | 0.6980 | 0.7186 | 0.4002 | **0.7656** |
|  | iAMPCN | 0.6709 | **0.7914** | 0.6118 | **0.7016** | **0.7262** | **0.4099** | 0.7621 |
| Antibiofilm | RF | 0.7536 | 0.7027 | 0.7703 | 0.7365 | 0.7273 | 0.4741 | 0.8121 |
|  | XGBoost | **0.8387** | 0.7027 | **0.8649** | 0.7838 | 0.7647 | 0.5752 | 0.8654 |
|  | iAMPCN | 0.8243 | **0.8243** | 0.8243 | **0.8243** | **0.8243** | **0.6486** | **0.8667** |
| Anticancer | RF | 0.6735 | **0.7763** | 0.6237 | 0.7000 | 0.7213 | 0.4047 | 0.7740 |
|  | XGBoost | **0.7184** | 0.7323 | **0.7129** | **0.7226** | **0.7252** | **0.4452** | **0.7927** |
|  | iAMPCN | 0.6830 | 0.7667 | 0.6441 | 0.7054 | 0.7224 | 0.4139 | 0.7698 |
| Antifungal | RF | 0.7398 | 0.4140 | **0.8544** | 0.6342 | 0.5309 | 0.2990 | 0.7134 |
|  | XGBoost | **0.7497** | 0.4702 | 0.8430 | 0.6566 | 0.5779 | 0.3375 | 0.7312 |
|  | iAMPCN | 0.7347 | **0.5368** | 0.8061 | **0.6715** | **0.6204** | **0.3561** | **0.7483** |
| Anti-Gram-negative | RF | 0.6282 | 0.7797 | 0.5386 | 0.6592 | 0.6958 | 0.3280 | 0.7046 |
|  | XGBoost | **0.6354** | 0.7742 | **0.5557** | 0.6649 | 0.6979 | 0.3381 | 0.7177 |
|  | iAMPCN | 0.6335 | **0.8058** | 0.5338 | **0.6698** | **0.7093** | **0.3529** | **0.7212** |
| Anti-Gram-positive | RF | 0.6304 | 0.7493 | 0.5607 | 0.6550 | 0.6847 | 0.3157 | 0.6905 |
|  | XGBoost | **0.6423** | 0.7592 | **0.5772** | 0.6682 | 0.6959 | 0.3421 | 0.7111 |
|  | iAMPCN | 0.6372 | **0.7995** | 0.5449 | **0.6722** | **0.7092** | **0.3561** | **0.7246** |
| Anti-HIV | RF | 0.8413 | 0.6667 | 0.8742 | 0.7704 | 0.7439 | 0.5529 | 0.8452 |
|  | XGBoost | 0.8333 | 0.5975 | **0.8805** | 0.7390 | 0.6960 | 0.4984 | 0.8157 |
|  | iAMPCN | **0.8489** | **0.7421** | 0.8679 | **0.8050** | **0.7919** | **0.6149** | **0.8633** |
| Anti-MRSA | RF | 0.8529 | 0.5472 | 0.9057 | 0.7264 | 0.6667 | 0.4851 | 0.8418 |
|  | XGBoost | **0.9000** | 0.6792 | **0.9245** | 0.8019 | 0.7742 | 0.6228 | **0.8768** |
|  | iAMPCN | 0.8600 | **0.8113** | 0.8679 | **0.8396** | **0.8350** | **0.6803** | 0.8597 |
| Antiparasitic | RF | 0.6575 | 0.5517 | 0.7126 | 0.6322 | 0.6000 | 0.2679 | 0.7181 |
|  | XGBoost | **0.7857** | 0.3793 | **0.8966** | 0.6379 | 0.5116 | 0.3223 | 0.7704 |
|  | iAMPCN | 0.7534 | **0.6322** | 0.7931 | **0.7126** | **0.6875** | **0.4309** | **0.7861** |
| Antiviral | RF | 0.7908 | 0.7793 | 0.7939 | 0.7866 | 0.7850 | 0.5732 | 0.8805 |
|  | XGBoost | 0.7982 | **0.7801** | 0.8027 | **0.7914** | **0.7890** | **0.5830** | 0.8808 |
|  | iAMPCN | **0.8248** | 0.7308 | **0.8448** | 0.7878 | 0.7750 | 0.5794 | **0.8865** |
| Anti-mammalian cells | RF | 0.6563 | 0.7653 | 0.5993 | 0.6823 | 0.7067 | 0.3698 | 0.7407 |
|  | XGBoost | **0.6782** | 0.7810 | **0.6294** | 0.7052 | 0.7260 | 0.4151 | 0.7546 |
|  | iAMPCN | 0.6748 | **0.7942** | 0.6173 | **0.7058** | **0.7297** | **0.4181** | **0.7665** |
| Chemotactic | RF | **1.0000** | 0.2143 | **1.0000** | 0.6071 | 0.3529 | 0.3464 | 0.7704 |
|  | XGBoost | **1.0000** | **0.5000** | **1.0000** | **0.7500** | **0.6667** | **0.5774** | 0.7755 |
|  | iAMPCN | **1.0000** | **0.5000** | **1.0000** | **0.7500** | **0.6667** | **0.5774** | **0.8163** |
| Endotoxin | RF | **0.8571** | 0.3750 | **0.9375** | 0.6563 | 0.5217 | 0.3780 | 0.6328 |
|  | XGBoost | 0.7143 | 0.6250 | 0.7500 | 0.6875 | 0.6667 | 0.3780 | **0.8008** |
|  | iAMPCN | 0.7059 | **0.7500** | 0.6875 | **0.7188** | **0.7273** | **0.4384** | 0.7617 |
| Insecticidal | RF | 0.8289 | 0.7590 | 0.8434 | 0.8012 | 0.7925 | 0.6046 | 0.8675 |
|  | XGBoost | 0.8243 | 0.7349 | 0.8434 | 0.7892 | 0.7771 | 0.5817 | 0.8782 |
|  | iAMPCN | **0.8514** | **0.7590** | **0.8675** | **0.8133** | **0.8025** | **0.6302** | **0.8936** |

Supplementary Table S36**. The Wilcoxon signed-ranks test for measuring the statistical difference of the performance between iAMPCN, RF and XGBoost models (features extracted by the ESM-2 model) in terms of Accuracy, F1, MCC, and AUC.**

| **Accuracy** |  |  |
| --- | --- | --- |
| Wilcoxon *p*-value | RF | XGBoost |
| XGBoost | 0.0103 | - |
| iAMPCN | 6.10E-05 | 0.0083 |
| **F1** |  |  |
| Wilcoxon *p*-value | RF | XGBoost |
| XGBoost | 0.0833 | - |
| iAMPCN | 0.00018 | 0.0052 |
| **MCC** |  |  |
| Wilcoxon *p*-value | RF | XGBoost |
| XGBoost | 0.0157 | - |
| iAMPCN | 6.10E-05 | 0.0076 |
| **AUC** |  |  |
| Wilcoxon *p*-value | RF | XGBoost |
| XGBoost | 0.0034 | - |
| iAMPCN | 0.00018 | 0.3028 |

# Supplementary Figure

**
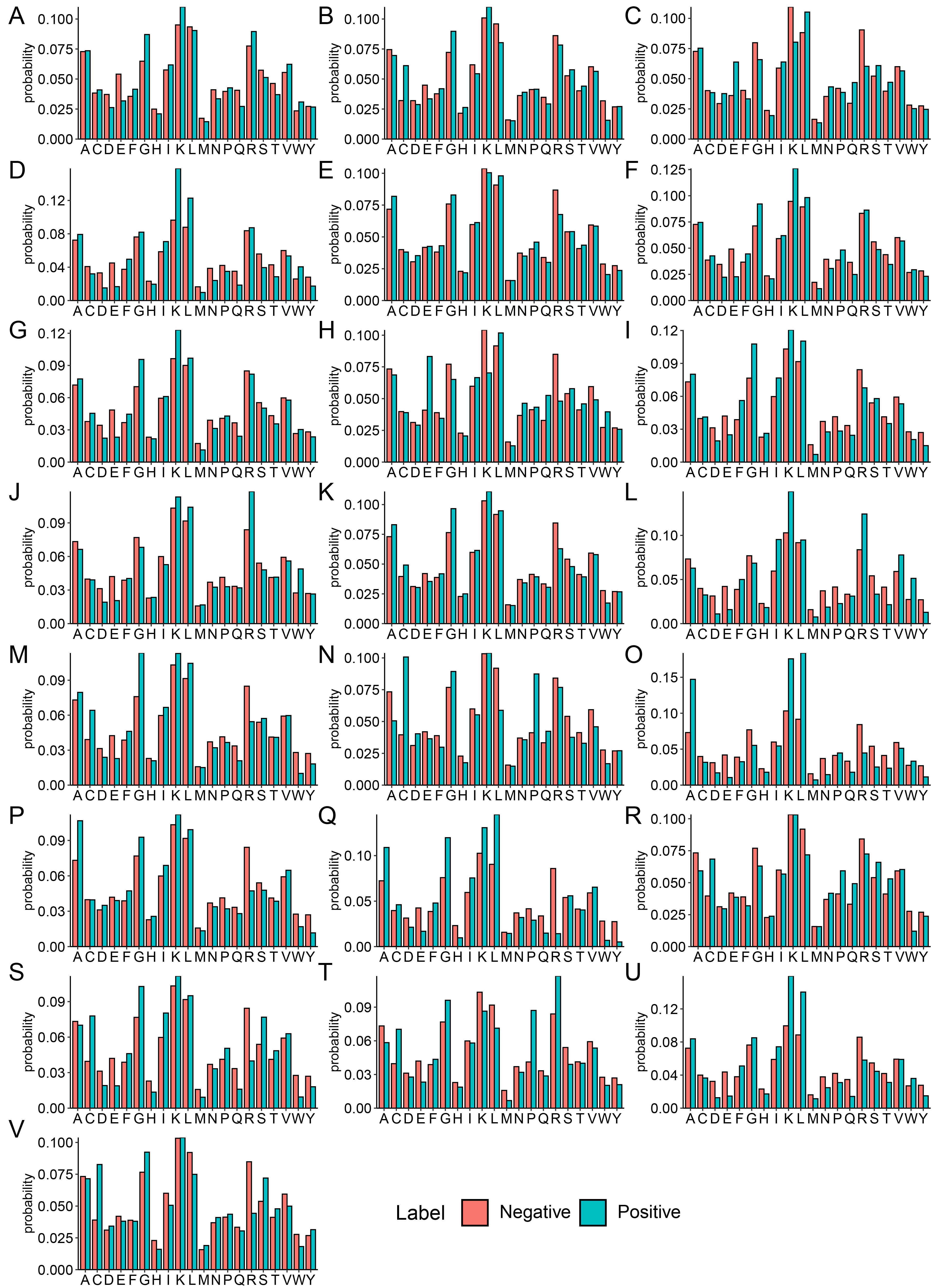
**

Supplementary Figure S1. **Amino acid distributions of positive and negative datasets of different AMP functional activities.** These functional activities include: (A) antibacterial, (B) antifungal, (C) antiviral, (D) anti-mammalian cells, (E) anticancer, (F) anti-Gram-negative, (G) anti-Gram-positive, (H) anti-HIV, (I) anti-MRSA, (J) anti-TB, (K) antiparasitic, (L) antibiofilm, (M)anticandia, (N) antimalarial, (O) antiplasmodial, (P) antiprotozoal, (Q) anurandefense, (R) chemotactic, (S) cytotoxic, (T) endotoxin, (U) hemolytic, and (V) insecticidal.

**
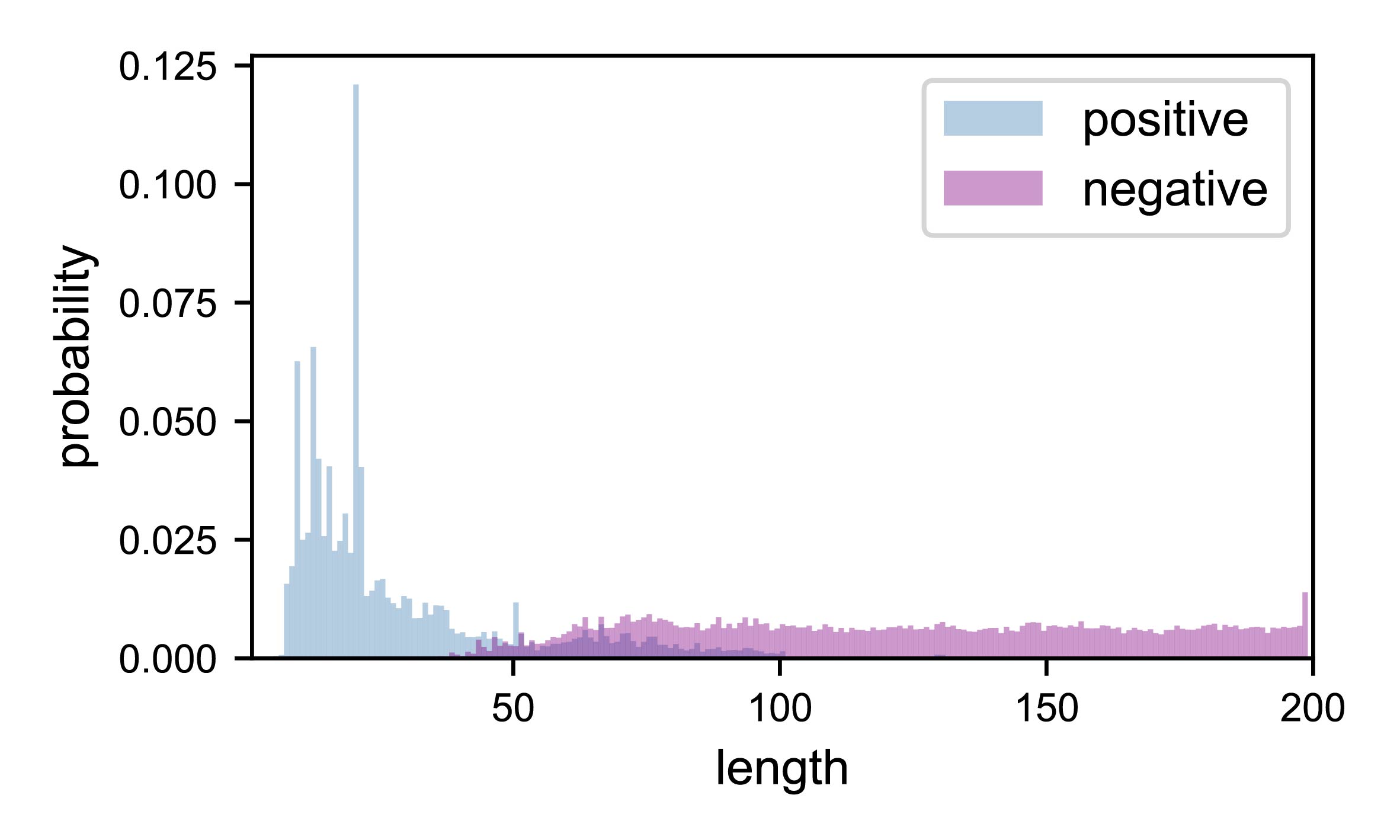
**

Supplementary Figure S2. **Sequence length distributions of AMPs and non-AMPs.**

**
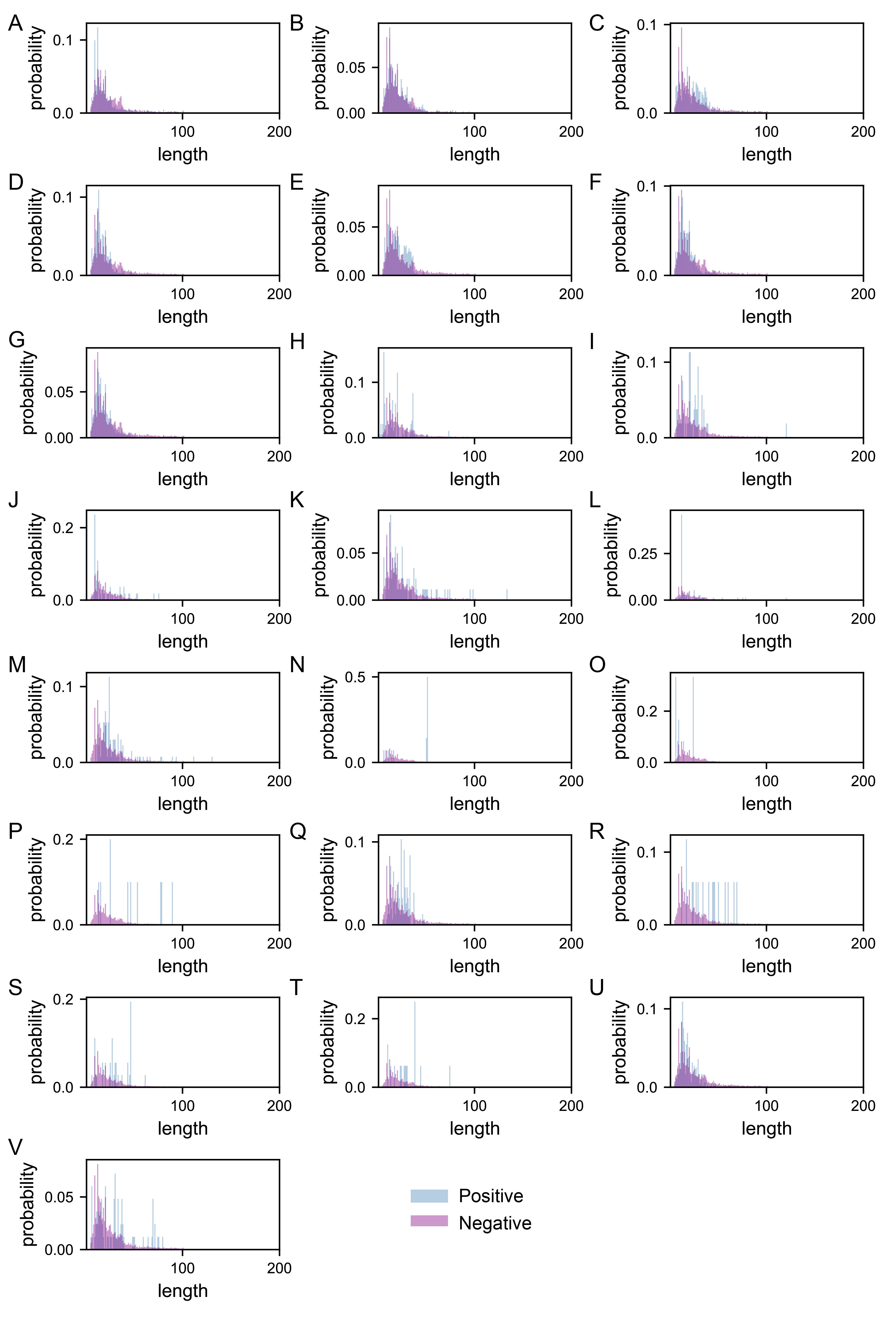
**

Supplementary Figure S3. **Sequence length distributions of positive and negative datasets for identifying different AMP functional activities.** These functional activities include: (A) antibacterial, (B) antifungal, (C) antiviral, (D) anti-mammalian cells, (E) anticancer, (F) anti-Gram-negative, (G) anti-Gram-positive, (H) anti-HIV, (I) anti-MRSA, (J) anti-TB, (K) antiparasitic, (L) antibiofilm, (M)anticandia, (N) antimalarial, (O) antiplasmodial, (P) antiprotozoal, (Q) anurandefense, (R) chemotactic, (S) cytotoxic, (T) endotoxin, (U) hemolytic, and (V) insecticidal.

**
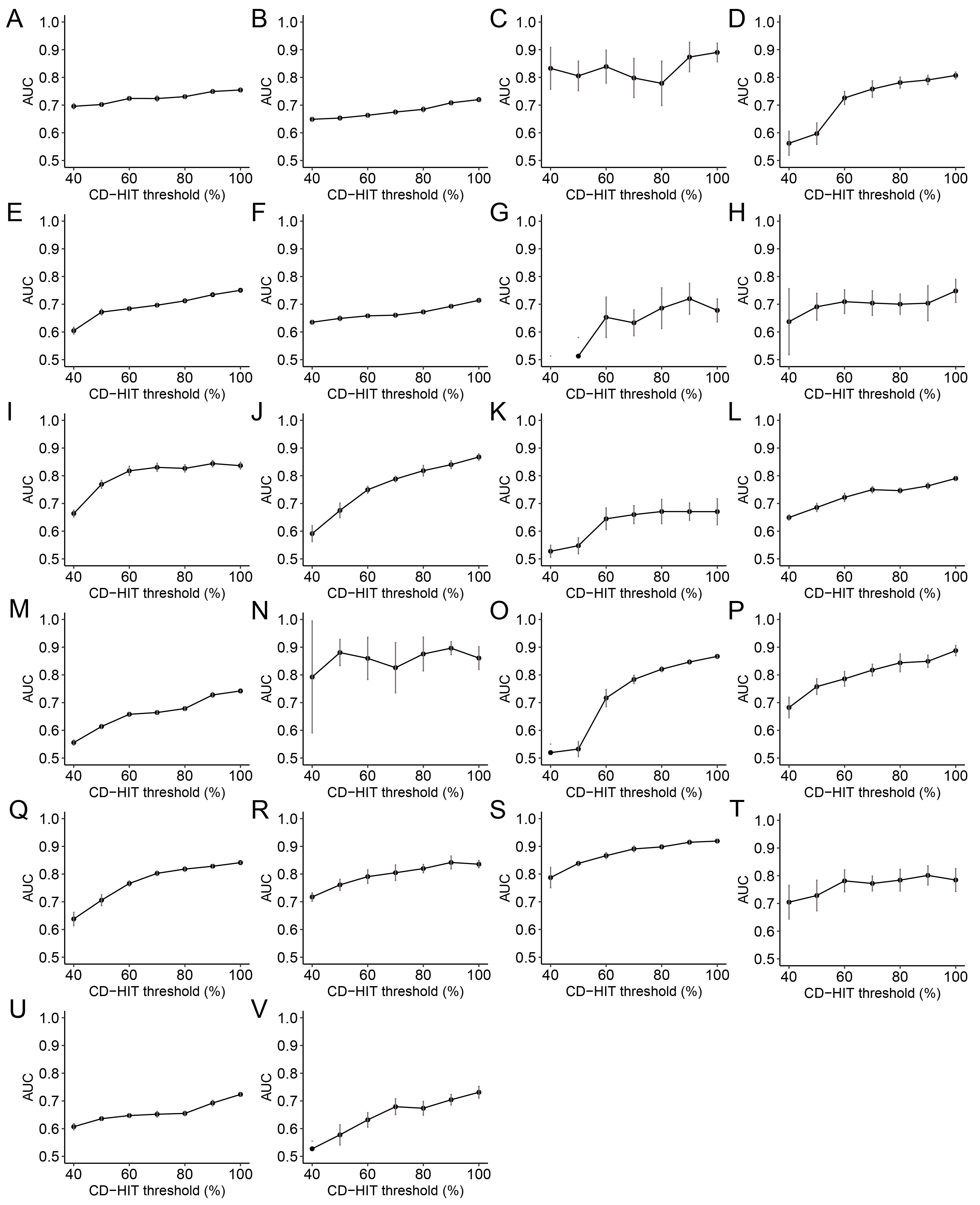
**

Supplementary Figure S4. **AUC values with different sequence similarity thresholds of different AMP functional activities based on test datasets.** These functional activities include: (A) anti-mammalian cells, (B) anti-Gram-negative, (C) antiplasmodial, (D) cytotoxic, (E) antibacterial. (F) anti-Gram-positive, (G) antiprotozoal, (H) endotoxin, (I) endotoxin, (J) anti-HIV, (K) anti-TB, (L) hemolytic, (N) anticancer, (N) antimalarial, (O) antiviral, (P) insecticidal, (Q) anticandidal, (R) anti-MRSA, (S) anuran defense, (T) chemotactic, (U) antifungal, and (V) antiparasitic.

**
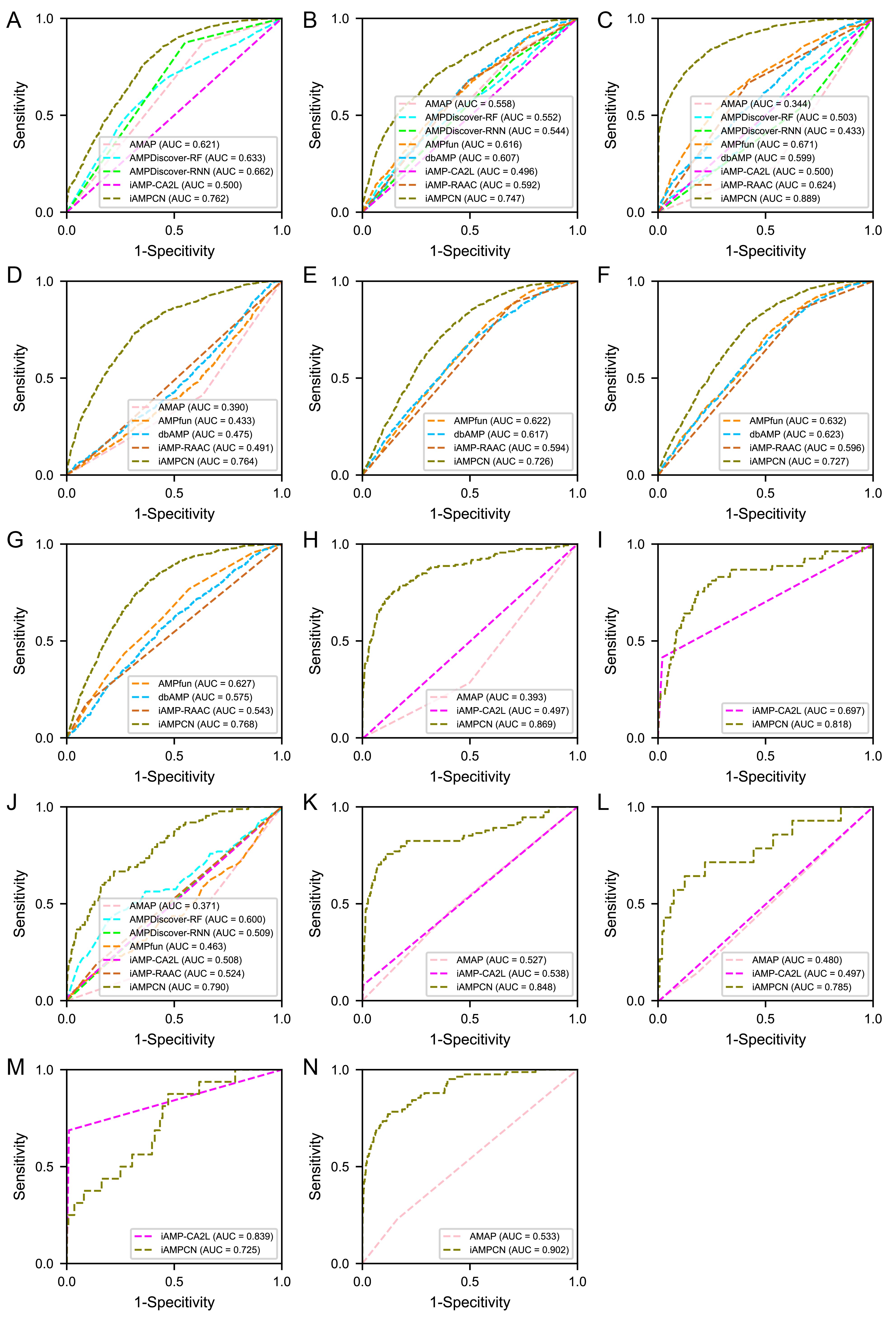
**

Supplementary Figure S5. **Comparison of ROC curves and AUC values of different AMP functional activities based on imbalanced independent test datasets.** These functional activities include: (A) antibacterial, (B) antifungal, (C) antiviral, (D) anticancer, (E) anti-Gram-positive, (F) anti-Gram-negative, (G) anti-mammalian cells, (H) anti-HIV, (I) anti-MRSA, (J) antiparasitic, (K) antibiofilm, (L) chemotactic, (M) endotoxin, and (N) insecticidal.


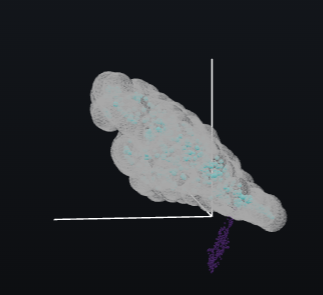


Supplementary Figure S6. **The screenshot of the points from the problematic cluster for predicting the antibacterial activity using the PHOENIX tool.**


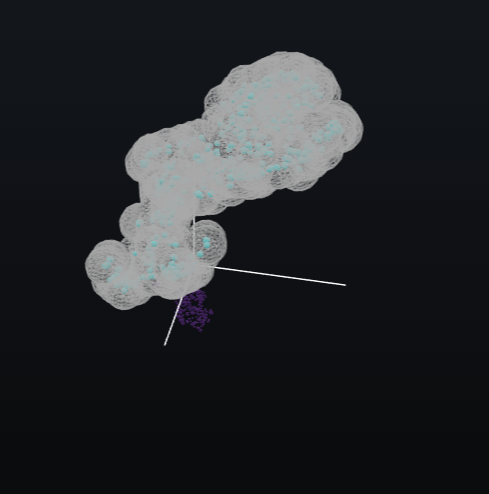


Supplementary Figure S7. **The screenshot of the points from the problematic cluster for predicting the antifungal activity using the PHOENIX tool.**


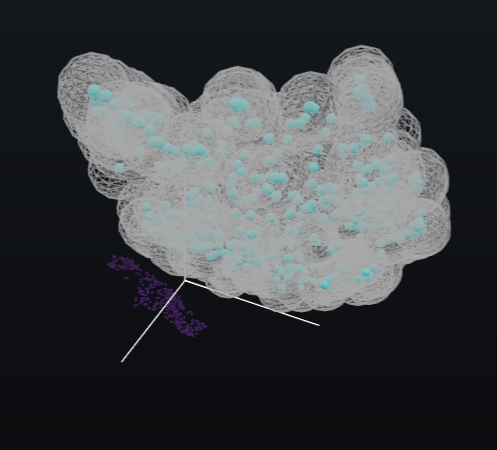


Supplementary Figure S8. **The screenshot of the points from the problematic cluster for predicting the activity of the anti-mammalian cells using the PHOENIX tool.**


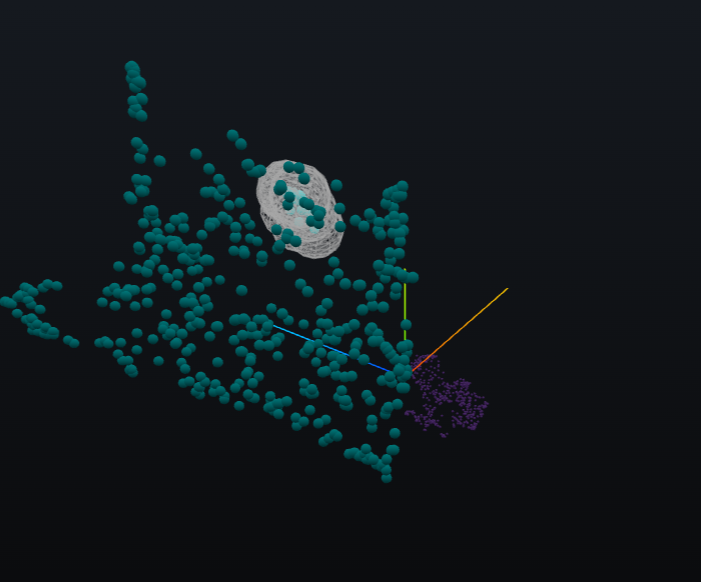


Supplementary Figure S9. **The screenshot of the points from the problematic cluster for predicting the anticancer activity using the PHOENIX tool.**


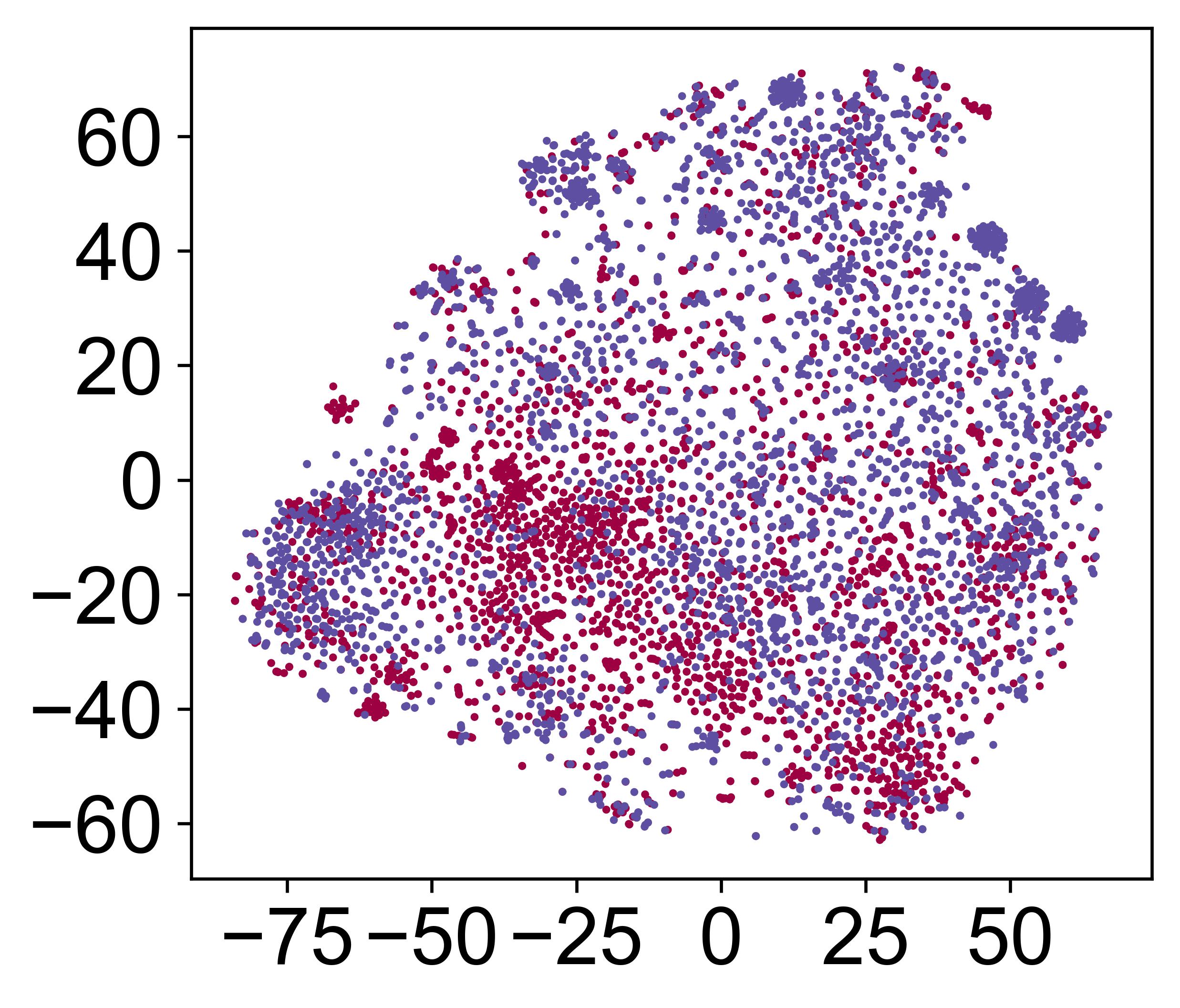


Supplementary Figure S10. **The *t*-distributed stochastic neighbor embedding (*t*-SNE) plot for the test dataset of the antibacterial activity.**

**
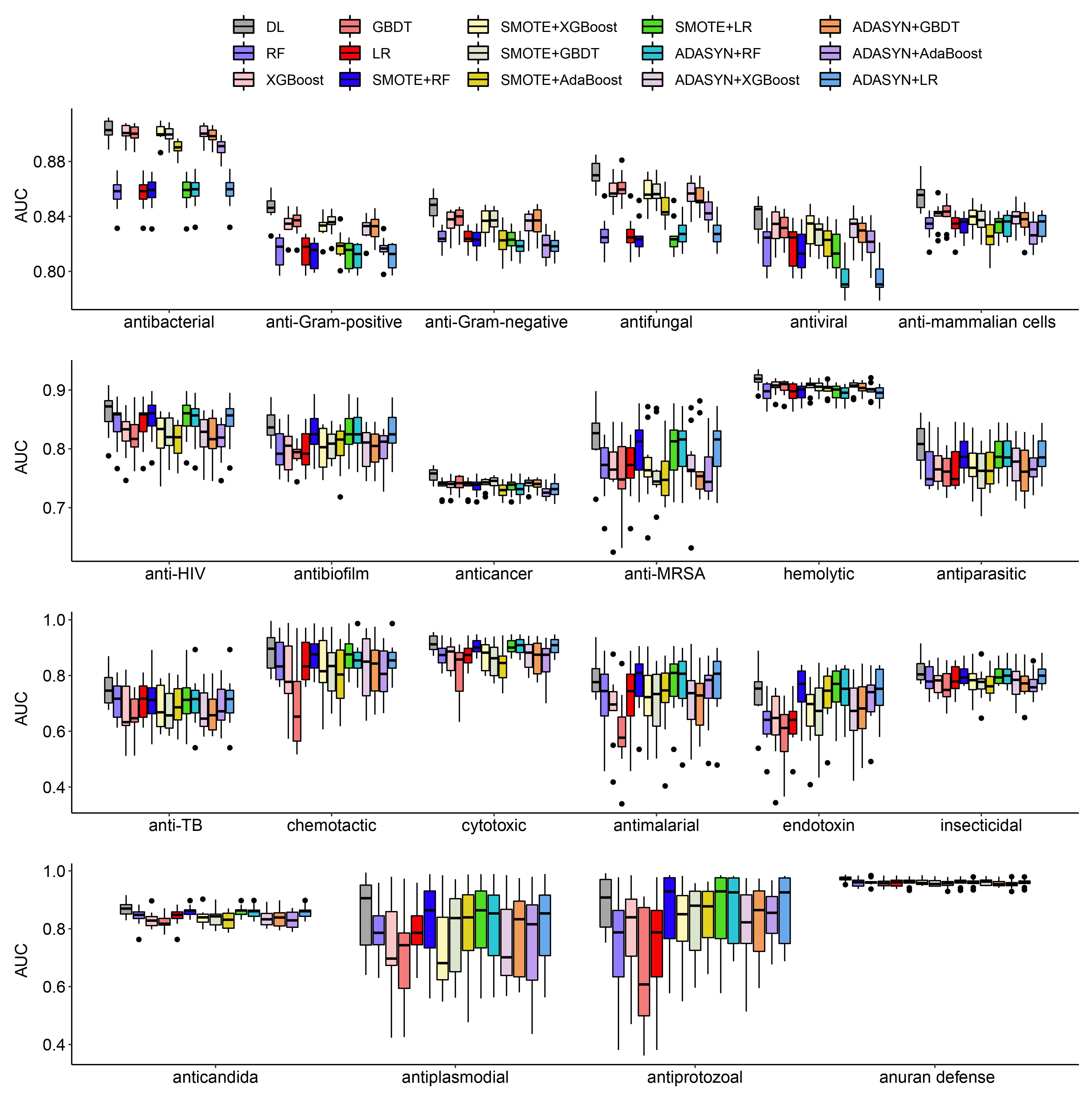
**

Supplementary Figure S11. **Comparison of AUC values of different AMP functional activities based on 10-fold cross validation test on training datasets.**

**
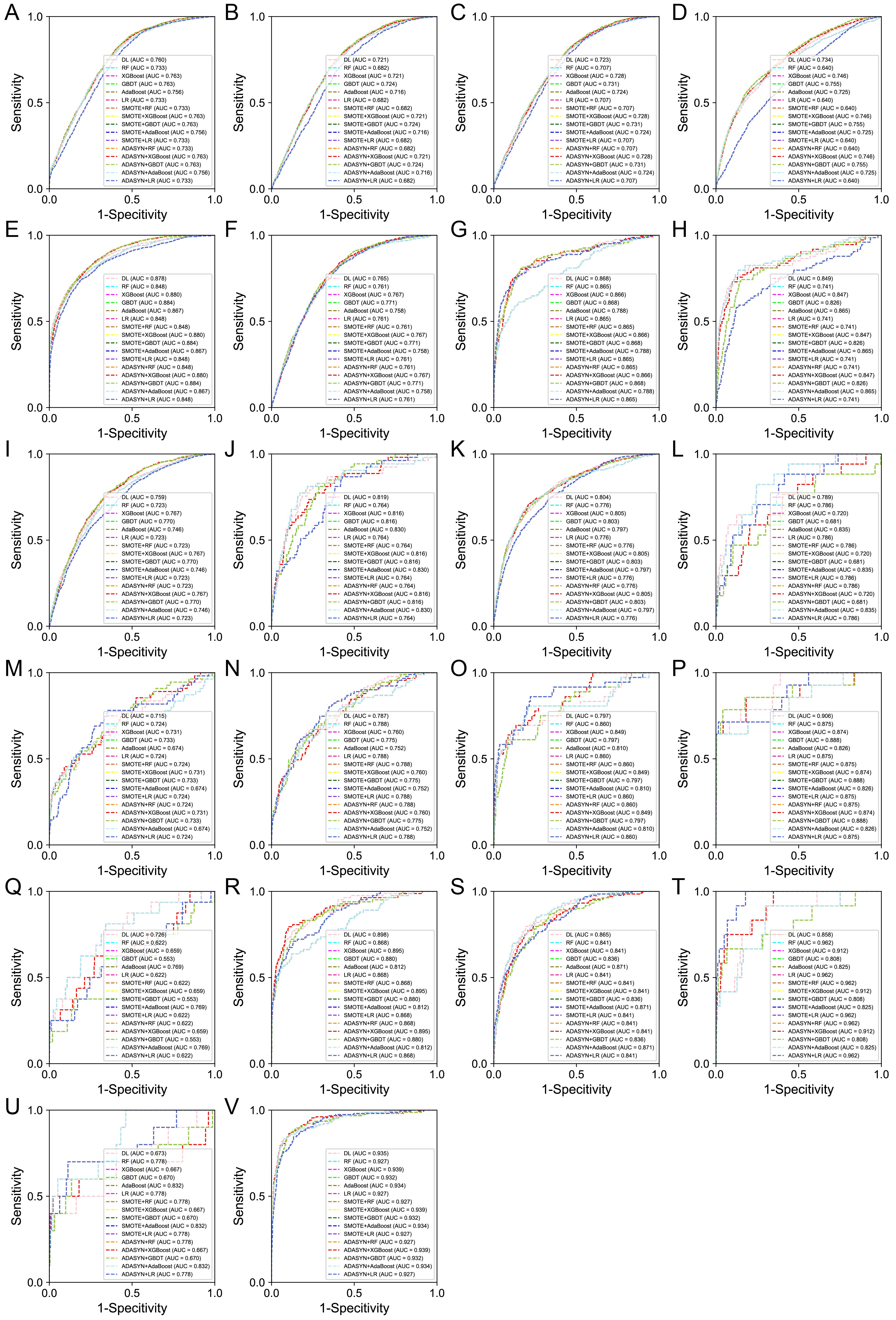
**

Supplementary Figure S12. **Comparison of ROC curves and AUC values of different machine learning algorithms based on different AMP functional activities test datasets.** These functional activities include: (A) antibacterial, (B) anti-Gram-positive, (C) anti-Gram-negative, (D) antifungal, (E) antiviral, (F) anti-mammalian cells, (G) anti-HIV, (H) antibiofilm, (I) anticancer, (J) anti-MRSA, (K) hemolytic, (L) chemotactic, (M) anti-TB, (N) antiparasitic, (O) cytotoxic, (P) antimalarial, (Q) endotoxin, (R) insecticidal, (S) anticandidal, (T) antiplasmodial, (U) antiprotozoal, and (V) anuran defense.
